# SHAP-Based Identification of Potential Acoustic Biomarkers in Patients with Post-Thyroidectomy Voice Disorder

**DOI:** 10.3390/diagnostics15162065

**Published:** 2025-08-18

**Authors:** Salih Celepli, Irem Bigat, Bilgi Karakas, Huseyin Mert Tezcan, Mehmet Dincay Yar, Pinar Celepli, Mehmet Feyzi Aksahin, Oguz Hancerliogullari, Yavuz Fuat Yilmaz, Osman Erogul

**Affiliations:** 1 Department of General Surgery, Gulhane Training and Research Hospital, Ankara 06010, Turkey; mdbilgikarakas@gmail.com (B.K.); hmert17@hotmail.com (H.M.T.); mehmetyar1997@gmail.com (M.D.Y.); oguzhancerli@gmail.com (O.H.); 2 Department of Biomedical Engineering, Graduate School of Science and Technology, TOBB University of Economics and Technology, Ankara 06510, Turkey; irembigat@etu.edu.tr (I.B.); erogul@etu.edu.tr (O.E.); 3 Department of Pathology, Gulhane Training and Research Hospital, Ankara 06010, Turkey; pcelepli@yahoo.com; 4 Department of Electrical and Electronic Engineering, Gazi University, Ankara 06560, Turkey; maksahin@gazi.edu.tr; 5 Department of Otorhinolaryngology, Gulhane Training and Research Hospital, Ankara 06010, Turkey; yfyilmaz@gmail.com

**Keywords:** post-thyroidectomy voice disorder (PTVD), acoustic signal processing, machine learning classification, Shapley Additive Explanations (SHAP), biomarker validation

## Abstract

**Objective:** The objective of this study was to identify potential robust acoustic biomarkers for functional post-thyroidectomy voice disorder (PTVD) that may support early diagnosis and personalized treatment strategies, using acoustic analysis and explainable machine learning methods. **Methods:** Spectral and cepstral features were extracted from /a/ and /i/ voice recordings collected preoperatively and 4–6 weeks postoperatively from a total of 126 patients. Various Support Vector Machine (SVM) and Boosting models were trained. SHapley Additive exPlanations (SHAP) analysis was applied to enhance interpretability. SHAP values from training and test sets were compared via scatter plots to identify stable candidate biomarkers with high consistency. **Results:** GentleBoost (AUC = 0.85) and LogitBoost (AUC = 0.81) demonstrated the highest classification performance. Performance metrics across all models were evaluated for statistical significance. DeLong’s test was conducted to assess differences between ROC curves. The features iCPP, aCPP, and aHNR were identified as stable candidate biomarkers, exhibiting consistent SHAP distributions in both training and test sets in terms of direction and magnitude. These features showed statistically significant correlations with PTVD (*p* < 0.05) and demonstrated strong effect sizes (Cohen’s d = −2.95, −1.13, −0.60). Their diagnostic relevance was further supported by post hoc power analyses (iCPP: 1.00; aCPP: 0.998). **Conclusions:** SHAP-supported machine learning models offer an objective and clinically meaningful approach for evaluating PTVD. The identified features may serve as potential biomarkers to guide individualized voice therapy decisions during the early postoperative period.

## 1. Introduction

Voice disorders following thyroid surgery in the absence of overt nerve injury are widely reported in the literature [1]. Post-thyroidectomy voice disorder (PTVD) is characterized by subjective symptoms such as difficulty in producing high-pitched sounds, vocal fatigue, and impaired voice quality [2]. However, a significant proportion of these patients present with normal findings on laryngoscopic examination, and current diagnostic methods are often inadequate in detecting subtle lesions, particularly those involving the external branch of the superior laryngeal nerve (EBSLN) [3,4]. In a prospective study evaluating 243 patients undergoing thyroidectomy, Šimić Prgomet et al. reported objective voice alterations in 82.3% of cases, even when neural pathways were preserved. This strongly supports the prevalence of functional PTVD among patients with laryngoscopically normal findings [5].

Acoustic parameters such as jitter, shimmer, the harmonic-to-noise ratio (HNR), and cepstral peak prominence (CPP) allow for objective and non-invasive evaluation of voice disorders [2,6,7]. However, the high-dimensional and nonlinear nature of voice signals limits the classification accuracy of these conventional methods.

Machine learning (ML) offers a powerful alternative approach in voice analysis due to its ability to learn nonlinear patterns in high-dimensional data [8,9,10]. Studies in the literature have reported over 85% accuracy in general dysphonia classification using ML algorithms [11,12,13]. Nevertheless, explainable and clinically applicable models specifically targeting PTVD remain scarce.

SHAP (SHapley Additive exPlanations) analysis enables interpretation of machine learning model decisions by quantifying the contribution of each feature. By visualizing individual feature impacts, SHAP promotes clinical transparency in model outputs. This approach has been successfully applied in sepsis prognosis models to help clinicians understand decision processes at the patient level [14].

Despite the increasing interest in AI-based voice diagnostics, the number of studies focusing specifically on functional PTVD using prospectively collected voice recordings remains extremely limited.

Furthermore, the current literature lacks SHAP-based explainability analyses applied to ML models for PTVD, as well as a systematic identification of acoustic biomarker candidates supported by statistical validity.

This gap hinders the clinical utility of ML models in distinguishing functional voice impairments in the absence of observable laryngeal pathology.

To address these shortcomings, in the present study, preoperative and postoperative voice recordings of patients diagnosed with PTVD—despite showing normal laryngoscopic findings and experiencing changes in VHI-10 scores—were analysed. Through ML classification and SHAP analysis, potential acoustic biomarker candidates associated with PTVD were identified.

Unlike previous work, this study integrates machine learning with explainability tools and effect size-based biomarker evaluation to provide a robust, multidimensional framework for functional PTVD classification.

This methodology enhances clinical interpretability, increases diagnostic sensitivity in subtle presentations, and promotes generalizability of the findings.

This study offers the following contributions:

This study enables early detection and referral for voice therapy in patients with functional voice impairment who appear laryngoscopically normal both before and after thyroidectomy.

This study also provides a data-driven, objective classification approach in the field of PTVD by proposing an explainable and clinically interpretable machine learning model for early diagnosis.

Multidimensional assessments using SHAP stability analysis and Cohen’s d effect size metrics further support the biomarker potential of the identified acoustic features by demonstrating their significant association with PTVD.

This study demonstrates the feasibility and utility of SHAP-based model interpretation in voice disorders, filling a critical gap in functional PTVD research and laying the groundwork for future clinical applications.

## 2. Materials and Methods

### 2.1. Study Design and Ethical Approval

This study was approved by the Clinical Research Ethics Committee of Gülhane Training and Research Hospital on 28 February 2023 (Approval No: 2022/42). Individuals aged between 18 and 60 who were scheduled for thyroid surgery were included. The inclusion criteria were as follows: no prior history of thyroid/parathyroid/cervical surgery, no history of head and neck radiotherapy, absence of known laryngeal pathology, normal findings on otorhinolaryngological examination, and no anatomical variations or defects in the orofacial region.

All data were anonymized, and no personally identifiable information was used in the analyses. In this context, original voice recordings were considered personal data under the Turkish Personal Data Protection Law No. 6698 (KVKK) and were not publicly shared. Instead, only acoustic features derived from the recordings—containing no identifiable information—were included in the analysis. Written informed consent was obtained from all participants. All procedures were conducted in accordance with the Declaration of Helsinki and national ethical guidelines [15].

#### Sample Size and Power Analysis

The required sample size for this study was determined using an a priori power analysis conducted with G*Power software (version 3.1.9.7). The analysis was designed to evaluate the difference between two paired groups (i.e., preoperative and postoperative data from the same participants). Assuming a medium effect size (dz = 0.5) based on Cohen’s definition [16], with a significance level of 5% (α = 0.05) and a statistical power of 95% (1 − β = 0.95), the minimum required sample size was calculated to be 54 individuals. A total of 126 cases were included in the study.

### 2.2. Timing of Voice Recordings

Preoperative voice recordings were obtained approximately one week prior to surgery in order to document baseline voice characteristics. Postoperative recordings were conducted between 4 and 6 weeks following thyroidectomy. This time frame was selected to capture both the optimal period for nerve regeneration and the peak manifestation of voice disorders. The timing was determined by considering the physiological healing process and the window in which voice assessment is most sensitive.

In addition, laryngeal electromyography (L-EMG), typically recommended beginning in the 3rd to 4th postoperative week, highlights the critical diagnostic window for detecting Wallerian degeneration and spontaneous denervation. During this period, L-EMG can be used with high accuracy to distinguish cricoarytenoid joint fixation from neural injury and to assess the risk of permanent paralysis [17]. Although L-EMG was not performed in this study, its relevance is discussed in reference to the existing literature.

According to clinical practice guidelines issued by the American Academy of Otolaryngology—Head and Neck Surgery (AAO-HNS), the optimal time frame for evaluating postoperative voice changes is between 2 weeks and 2 months. Early recognition of nerve injury not only enhances voice and overall quality of life but also enables timely interventions such as voice therapy and injection laryngoplasty. This period is considered critical for reducing both false positives and the risks associated with delayed intervention [18]. It has been demonstrated that the majority of laryngeal nerve regeneration occurs within the first 4 weeks postoperatively, and this process produces measurable effects on voice function [19].

Furthermore, by the fourth postoperative week, both subjective and objective voice impairments typically become more pronounced. Notably, Voice Handicap Index (VHI-10) scores have been reported to exceed the threshold for clinically significant deterioration in approximately 40–55% of patients at this stage [2,20].

In recent years, the use of cepstral analysis and subjective measures such as the Voice Handicap Index (VHI) has become increasingly common in the early diagnosis of postoperative voice disorders. These tools offer a comprehensive framework for evaluating voice changes associated with PTVD [2,21].

Collectively, these findings suggest that the fourth postoperative week presents a clinically appropriate window for the early identification of functional voice disorders such as post-thyroidectomy voice disorder (PTVD).

### 2.3. Voice Handicap Index (VHI-10) Assessment

To evaluate functional voice disorders, the Voice Handicap Index-10 (VHI-10), a widely used, time-efficient, and highly valid self-report instrument, was employed [22]. The VHI-10 was administered to all 126 patients at two time points: within 1 week prior to surgery and between 4 and 6 weeks postoperatively. The questionnaire consists of 10 items, each rated on a 5-point Likert scale ranging from 0 (never) to 4 (always), and was completed individually by patients based on self-assessment. Total scores (ranging from 0 to 40) were used in the analyses to quantitatively evaluate changes in the patients’ subjective perception of their voice. The reliability of the Turkish version of the VHI-10 has been previously validated by Kılıç et al. [21,23,24].

### 2.4. Videolaryngostroboscopic Evaluation

Videolaryngostroboscopy is a widely used diagnostic method that allows for structural and functional assessment of vocal fold vibrations and plays a key role in the evaluation of voice disorders [25]. All patients underwent videolaryngostroboscopic examination during the week preceding surgery and again between 4 and 6 weeks postoperatively. To minimize observer bias, all videolaryngostroboscopic assessments were performed by the same otolaryngologist, who was blinded to the patient group information. Imaging was conducted using a high-resolution endoscopic system (Karl Storz TELE PACK PAL, Model No: 200430 20, Karl Storz SE & Co. KG, Tuttlingen, Germany).

At both time points, vocal fold mobility and structural integrity were evaluated as being within normal limits in all cases. However, this finding underscores the fact that functional dysphonias may not always be detectable via VLS.

### 2.5. Voice Data Acquisition and Preprocessing

In this study, only the sustained /a/ and /i/ vowel sounds were recorded, and all acoustic analyses were limited to these two phonations. Recordings were conducted in an acoustically isolated environment with background noise levels maintained below 40 dBA. Voice samples were collected using a SHURE SV100 multipurpose microphone (Shure Inc., Niles, IL, USA) with a frequency range of 50–15,000 Hz, impedance of 600 Ω, and sensitivity of −52 dBV/Pa. The recordings were made at a sampling rate of 44.1 kHz and 16-bit resolution, with a fixed mouth-to-microphone distance of 15 cm. The microphone’s low sensitivity (−52 dBV/Pa) helped minimize environmental noise interference and enhanced the reliability of the target voice signal.

Prior to analysis, all recordings were preprocessed using the Praat software (version 6.3.10, Boersma & Weenink, University of Amsterdam) [26]. Data processing was carried out exclusively on a CPU-based system (Intel Core i7-6700HQ, 16 GB DDR4 RAM, Windows 10 64-bit).

### 2.6. Acoustic Feature Extraction

#### 2.6.1. Noise Filtering

To eliminate low-frequency environmental noise and 50 Hz electrical interference originating from the power supply, a fourth-order Butterworth high-pass filter with a cutoff frequency of 50 Hz was applied. Filtering was performed on the voice signals—recorded at a sampling rate of 44.1 kHz—using the filtfilt() function in MATLAB (R2023a, MathWorks Inc., Natick, MA, USA) to ensure zero-phase, bidirectional processing. This approach preserved the phase characteristics of the signal without distortion.

The Butterworth filter was chosen due to its minimal ripple in the passband, ensuring a smooth frequency response. Each voice recording was processed in its entirety, and the filtering procedure was uniformly applied across all samples. This preprocessing step provided a stable and reliable basis for subsequent acoustic feature extraction [27]. Given that the microphone’s lower limit is 50 Hz, this filtering step did not result in a loss of meaningful voice signal but effectively removed potential low-frequency noise components.

#### 2.6.2. Frequency Band-Based Acoustic Feature Extraction

The voice signals were segmented into three primary frequency bands: the low-frequency band (50–250 Hz), which carries information related to the fundamental frequency (*f*_0_) and plays a role in syllable structure and word boundary perception [28]; the mid-frequency band (250–4000 Hz), which encompasses the formant structures where the energy of speech is most concentrated [29,30]; and the high-frequency band (4000–15,000 Hz), which conveys speech naturalness, sharpness, and speaker-specific characteristics [31]. These frequency ranges were defined based on their established physiological and perceptual functions, as reported in previous studies [28,29,30,31].

#### 2.6.3. Power Spectral Density (PSD) and the Burg Algorithm

To analyse the frequency components of the voice signals, each recording was subjected to autoregressive (AR) modelling. AR model parameters were estimated using the arfit() function [32], which tests model orders within the range of [2,3,4,5,6,7,8,9,10,11,12,13,14,15,16,17,18,19,20,21,22,23,24,25,26,27,28,29,30,31,32,33,34,35,36,37,38,39,40] and selects the optimal order based on the Schwarz Bayesian Criterion (SBC). The optimal model order was determined individually for each signal and subsequently used for parametric power spectral density (PSD) estimation.

PSD computations based on the AR model parameters were performed using the pburg() function, which employs the Burg algorithm. The analysis was conducted on voice signals recorded at a sampling rate of 44.1 kHz, corresponding to approximately 88,200 data points over an average duration of 2 s. While the 44.1 kHz sampling rate theoretically enables analysis of frequency components up to 22.05 kHz in accordance with the Nyquist theorem, the effective upper limit of meaningful spectral content is constrained by the microphone’s physical response range (50–15,000 Hz). Therefore, spectral analyses were conducted within this valid frequency range, ensuring both theoretical and practical consistency.

The pburg() function, based on the Burg algorithm, was selected for its suitability in modelling short-duration vocal signals, offering high-frequency resolution and stable spectral estimates. Particularly in pitch-synchronous speech segments, the Burg method allows for accurate modelling of formant frequencies and spectral structures. The performance of the Burg algorithm and its role in parametric spectral estimation have been comprehensively discussed by Paliwal [33,34].

The PSD analysis was performed in MATLAB (R2023a, MathWorks Inc., Natick, MA, USA). Unlike conventional methods that rely on FFT-based spectral analysis and short-time windowing, the Burg algorithm provides high-frequency resolution without the need for temporal windowing, making it especially effective and stable for short vocal signals [34].

#### 2.6.4. Comprehensive Extraction of Acoustic, Spectral, and Cepstral Features

The acoustic features analysed in this study provide a multidimensional characterization across spectral, temporal, and cepstral domains. For each frequency band, energy- and power-related features were derived from the power spectral density (PSD), including total area (integration under the PSD curve), maximum peak, and mean peak values. In addition, standard acoustic features widely reported in the literature were also extracted.

In terms of the fundamental frequency and formant structure, the following features were computed: the fundamental frequency (*f*_0_) [35], first and second formant frequencies (F1, F2), F1/F2 ratio, and bandwidths of both formants (F1BW and F2BW) [26,36]. Noise-related parameters included measures of voice instability such as the harmonic-to-noise ratio (HNR) [26,37,38], shimmer local, shimmer APQ3, shimmer APQ5, jitter local, jitter RAP, and jitter PPQ5 [39,40].

Additionally, spectral features were computed, including the spectral centroid, spectral flux [41,42], spectral flatness [43], spectral skewness [41], and spectral entropy [44]. Finally, cepstral analysis was conducted to extract the cepstral peak prominence (CPP) value [26,45].

A detailed technical description of all features is provided in Supplementary File S1. Each feature was computed using standardized parameters and established software tools and functions (Praat v6.3.10, Boersma & Weenink, University of Amsterdam [26]; MATLAB R2023a, Troparion [39]). Both the Troparion library and the CPP analysis script developed by Heller Murray are open-source, thereby enhancing the reproducibility of the analysis and enabling cross-study comparisons.

The selected feature extraction methods offer a comprehensive acoustic spectrum by capturing the periodic structures, formant resonances, spectral energy distribution, and temporal instability components of the voice signal.

### 2.7. Classification Using Machine Learning

In this study, machine learning algorithms were employed to classify preoperative and postoperative voice samples. The dataset consisted of a total of 252 voice recordings, with an equal number of samples from each class (preoperative: *n* = 126; postoperative: *n* = 126), ensuring class balance and minimizing learning bias during model training.

All machine learning procedures were implemented using MATLAB (R2025a; MathWorks Inc., Natick, MA, USA). The following classification models were utilized and compared based on their performance: Cubic Support Vector Machine (SVM), Quadratic SVM, Radial Basis Function (RBF) SVM, GentleBoost, and LogitBoost. These algorithms were selected due to their strong theoretical foundations and proven ability to capture complex relationships among features.

Rather than adopting a traditional binary classification based solely on the presence or absence of PTVD (i.e., PTVD-negative vs. PTVD-positive), the primary objective of this study was to differentiate between preoperative and postoperative voice recordings obtained from the same individuals. Following classification, SHAP (SHapley Additive exPlanations) analysis was employed to identify the most influential acoustic features contributing to model performance.

Subsequently, the clinical relevance of these SHAP-prioritized features was assessed through statistical analyses (e.g., Cohen’s d), focusing on their association with patients clinically diagnosed with PTVD. Importantly, PTVD diagnosis was not based solely on acoustic data but was established through a multidimensional clinical evaluation involving videolaryngostroboscopy (VLS), VHI-10 scores, and patient-reported symptoms.

Therefore, the aim of this study extended beyond evaluating model performance; it sought to identify and clinically validate potential acoustic biomarkers associated with PTVD, thereby bridging the gap between machine learning-based acoustic analysis and clinically grounded diagnostic outcomes. The integration of SHAP-based explainability with clinical validation has precedents in the recent literature. For instance, SHAP and LIME have been used for identifying acoustically derived biomarkers in non-invasive voice-based diagnosis of adult asthma [46].

#### 2.7.1. Data Partitioning and 3-Fold Cross-Validation

In all modelling procedures, the dataset was split into 80% training and 20% testing sets. Within the training set, 3-fold cross-validation was performed to evaluate the training performance of the models. This entire process was repeated over 100 iterations. In each iteration, data partitioning, feature selection, and model outputs were randomly re-evaluated, and the final performance metrics were reported as the average across all repetitions.

To ensure reproducibility, randomization was controlled by fixing the random seed to 2 and using the “twister” generator in all analyses.

#### 2.7.2. Feature Selection and Normalization

To prevent data leakage and preserve the generalizability of the models to real-world data, feature selection was performed exclusively on the training set in each iteration. Initially, all features were normalized using the z-score method. Test set normalization was carried out using the mean and standard deviation values calculated from the training set.

Feature selection was performed using the Least Absolute Shrinkage and Selection Operator (LASSO) [47], applied only to the normalized training data. The selected features were then projected onto the test set. LASSO was implemented with a fixed L1 penalty (Alpha = 1), and the regularization parameter (Lambda) was optimized via 5-fold cross-validation to minimize the deviance error.

In SVM models, the normalized versions of the LASSO-selected features were used. In contrast, Boosting models were trained using the unnormalized values of the features selected by LASSO.

#### 2.7.3. Definition and Theoretical Foundations of the Models Used

Support Vector Machines (SVMs) are supervised learning algorithms that aim to identify the optimal hyperplane that maximizes the margin between classes. Given a training dataset $\left(x_{i},y_{i}\right),i=1,\ldots ,l$, where $x_{i}\in R^{n}$ and $y_{i}\in \{-1,1\}$, the SVM optimization problem is defined as in Equation (1).

(1) $$\underset{w,b,\xi }{min}\frac{1}{2}\|w{\|}^{2}+C\sum_{i=1}^{l}\xi_{i}\;s.t.\;y_{i}\left(w^{⊤}\phi \left(x_{i}\right)+b\right)\geq 1-\xi_{i},\xi_{i}\geq 0$$

Here, ϕ(⋅) represents a kernel function that maps data points into a higher-dimensional feature space. Commonly used kernel functions include the polynomial kernel shown in Equation (2) and the Radial Basis Function (RBF) kernel presented in Equation (3).

(2) $$K\left(x_{i},x_{j}\right)={\left(\gamma x_{i}^{⊤}x_{j}+r\right)}^{d}$$

(3) $$K\left(x_{i},x_{j}\right)=exp\left(-\gamma {\left\|x_{i}-x_{j}\right\|}^{2}\right)$$

Quadratic (*d* = 2) and Cubic (*d* = 3) kernel SVMs are considered specific cases of the polynomial kernel function [48].

Boosting algorithms aim to construct a strong classifier by sequentially combining multiple weak learners. GentleBoost and LogitBoost are variants of the AdaBoost method.

In GentleBoost, a regression tree is trained at each iteration to minimize the weighted least squares error, and sample weights are updated in a smooth manner. The fundamental formulation is defined by Equation (4).

(4) $$F_{m}(x)=F_{m-1}(x)+\nu h_{m}(x)$$

Here, ν denotes the learning rate, and $h_{m}(x)$ represents the weak learner (e.g., a decision tree) [49].

LogitBoost generates a probabilistic prediction by minimizing the logistic regression loss. At each iteration, the log odds are updated using weighted least squares regression. The fundamental formulation is defined in Equation (5) [49].

(5) $$p(x)=\frac{1}{1+exp(-F(x))},logit(p)=log\left(\frac{p}{1-p}\right)=F(x)$$

These formulations provide the mathematical foundation for the ability of SVMs to learn nonlinear boundaries in high-dimensional spaces and for the ensemble learning strategies employed by Boosting algorithms [48,49].

#### 2.7.4. Model Hyperparameters

SVM models were trained using the Sequential Minimal Optimization (SMO) algorithm. Kernel scaling was automatically determined for all models. In polynomial kernel SVMs, the degree parameter was set to 2 (Quadratic) and 3 (Cubic). In the RBF SVM model, the Gaussian kernel was used as the kernel function.

For the GentleBoost and LogitBoost models, the templateTree function was utilized to control the structural properties of the weak learners. Each decision tree was constrained to a minimum of 6 parent nodes, a minimum of 3 leaf nodes, and a maximum of 10 splits. Both pruning (Prune = “off”) and leaf merging (MergeLeaves = “off”) were disabled. This configuration allowed each weak learner to capture meaningful patterns in the training data with greater detail, thereby enhancing its information-carrying capacity. Disabling MergeLeaves enables sharper decision boundaries and increases model flexibility, while disabling Prune ensures that each learner contributes to the ensemble without being simplified. This comprehensive setup supports the development of highly expressive models with fewer learning cycles while limiting overfitting through restricted tree depth, ultimately offering improved overall performance. Details regarding the number of learning cycles and learning rates are provided in Table 1, while all other parameters were left at their default values. The Reproducible option was set to “true” for all analyses.

Hyperparameter optimization was conducted experimentally by balancing classification performance and overfitting risk. In SVM models, the Box Constraint (C) and kernel parameters were systematically tested over a range of values, and model validation performance was compared. Similarly, for the GentleBoost and LogitBoost models, various combinations of learning rate, number of learning cycles, and weak learner configurations were evaluated to identify the best-performing setup. This entire process was carried out using an experimental grid search approach based on user-defined hyperparameter ranges. The objective was to enhance both model accuracy and generalizability.

#### 2.7.5. Performance Evaluation Metrics

Model performance was assessed based on the average of 100 iterations using the following metrics: accuracy, precision, sensitivity (recall), specificity, F1-score, and the area under the receiver operating characteristic curve (AUC). These metrics were computed separately for the 3-fold cross-validation on the training set and for the test set.

The formulas used for these performance metrics are provided below:

(6) $$\mathrm{Accuracy}\;=\frac{TP+TN}{TP+TN+FN+FP}$$

(7) $$\mathrm{Precision}\;=\frac{TP}{TP+FP}$$

(8) $$\mathrm{Recall}\;=\frac{TP}{TP+FN}$$

(9) $$\mathrm{Specificity}\;=\frac{TN}{TN+FP}$$

(10) $$F1\text{-}\mathrm{score}\;=2\times \frac{\;\mathrm{precision}\;\times \;\mathrm{recall}\;}{\;\mathrm{precision}\;+\;\mathrm{recall}\;}$$

### 2.8. Model Explainability via SHAP Analysis

To enhance the interpretability of machine learning model decisions and identify influential features, SHAP (SHapley Additive exPlanations) analysis was performed. For SVM-based models (Cubic, Quadratic, and RBF), kernel SHAP was applied, while tree SHAP was used for Boosting-based models (GentleBoost and LogitBoost) [50]. All computations were conducted separately for the training and test sets using the shapley() function in MATLAB. As SHAP explanations are inherently model-specific, the interpretability outputs were tailored to each model’s unique decision mechanism [51].

SHAP analysis has been successfully applied in various recent clinical modelling studies to improve both individual- and population-level interpretability. For example, Hu et al. utilized SHAP values in their early prognosis models for sepsis to interpret the contributions of specific features [14].

In each iteration, SHAP values for features selected via LASSO were calculated separately for the training and test sets. The mean SHAP values were then used to analyse overall model behaviour. This process was repeated across 100 iterations for each model.

To incorporate both local (instance-level) and global (average-level) interpretability, model behaviour was assessed using the mean SHAP values across the training and test sets. Features that were selected in more than 75% of the iterations were visualized in SHAP heatmaps to illustrate general contribution patterns. However, in the identification of stable biomarker candidates, only high-performing models (AUC ≥ 0.80) were considered. In these models, features exhibiting both high and directionally consistent SHAP values in both sets were evaluated via scatter plot analyses and defined as “stable.”

In the literature, AUC values are typically interpreted as follows: 0.5–0.7 as “poor discrimination,” 0.7–0.8 as “acceptable,” 0.8–0.9 as “excellent,” and >0.9 as “outstanding” [52,53].

Stability assessment was conducted not only via correlation coefficients but also through scatter plots comparing mean SHAP values from the training and test sets. Notably, features located near the diagonal line (x = y) and positioned in the upper-right quadrant—indicating high and consistent SHAP contributions in both datasets—were considered stable. This approach aligns with SHAP-based stability assessments in the literature; for instance, Scheda and Diciotti [54] demonstrated how feature stability can be established by comparing training and test SHAP means derived from repeated nested cross-validation. This method reinforces not only frequently selected but also highly impactful and behaviourally consistent features as potential biomarkers.

### 2.9. Evaluation of the Clinical Relevance of Stable Features Identified via SHAP Analysis

Machine learning models were trained to classify preoperative and postoperative voice samples, and SHAP values were used to interpret the contribution of individual features to the model’s classification decisions. Although SHAP-based analysis identified features with high stability in the classification task, the clinical significance of these features was further assessed using effect size metrics calculated from their raw (original) values. In the literature, SHAP values have been utilized not only to interpret model contributions but also to evaluate clinical differences between groups, as demonstrated by Hu et al. [14]. Similarly, Leite et al. used clinical relevance metrics to assess the discriminative power of features in machine learning-based voice disorder classification [8].

To evaluate the clinical relevance of highly stable features, the Cohen’s d effect size was calculated based on the original values of those features—rather than their SHAP values—by comparing the PTVD (+) and PTVD (−) groups.

Before computing Cohen’s d, the assumption of normality was tested using the Shapiro–Wilk test. Since none of the features followed a normal distribution (*p* < 0.05), Cohen’s d was used solely as a descriptive indicator of effect size, while statistical significance was assessed using the non-parametric Mann–Whitney U test. The formula for Cohen’s d is provided in Equation (11).

(11) $$\mathrm{Cohen}\prime s\;d=\frac{\mu_{1}-\mu_{2}}{SD_{\mathrm{pooled}\;}}$$

Here, $\mu_{1}$ and $\mu_{2}$ represent the mean feature values in the PTVD (+) and PTVD (−) groups, respectively, while $SD_{\mathrm{pooled}\;}$ denotes the pooled standard deviation of the two groups.

As commonly accepted in behavioural and biomedical sciences, a threshold of Cohen’s d ≥ 0.5 was adopted to identify features with at least a moderate effect size [55].

Through this multidimensional approach:

(1) Features that demonstrated high contribution and stability in the classification model were identified via SHAP analysis;
(2) The degree to which these features produced clinically meaningful group differences in PTVD diagnosis was assessed using Cohen’s d.

This comprehensive evaluation strategy enabled the identification of features that not only significantly contributed to model performance (via SHAP analysis) but also exhibited clinically meaningful differentiation between PTVD groups (via Cohen’s d) [55,56,57].

### 2.10. Determining Clinical Discriminative Power via ROC Analysis

Following the computation of effect sizes (Cohen’s d) based on the original values of stable features identified in SHAP analysis, a Mann–Whitney U test (*p* < 0.05) was performed to determine statistical significance. ROC analysis was subsequently applied to the features found to be statistically significant, in order to assess their diagnostic performance as potential clinical biomarkers for distinguishing PTVD (+) individuals.

This analysis aimed to evaluate the clinical discriminative power of these features and to determine optimal decision thresholds that could be used in practice. The assessment was based on the area under the ROC curve (AUC), sensitivity, specificity, and the Youden index [14,52,58].

### 2.11. TRIPOD-AI Compliance and Reporting

**Figure 1:**
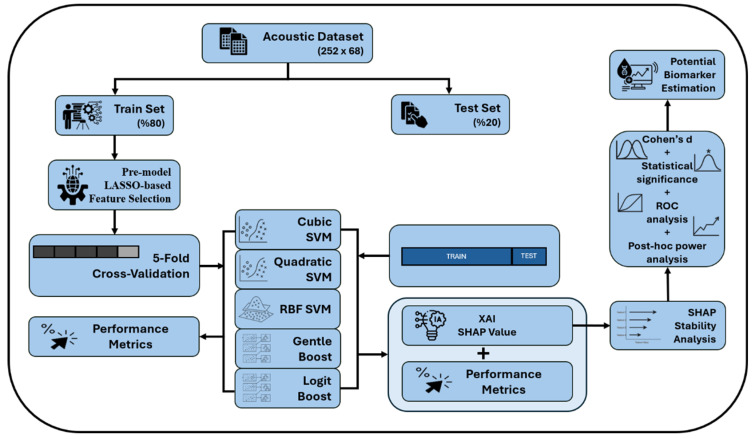
Machine learning workflow diagram (LASSO: Least Absolute Shrinkage and Selection Operator; SVM: Support Vector Machine; RBF: Radial Basis Function; XAI: explainable artificial intelligence; SHAP: SHapley Additive exPlanations; ROC: receiver operating characteristic).

This study was conducted in accordance with the TRIPOD-AI (Transparent Reporting of a Multivariable Prediction Model for Individual Prognosis or Diagnosis—Artificial Intelligence extension) guidelines, which aim to ensure transparent reporting of multivariable prediction models developed for individual prognosis or diagnosis using artificial intelligence. The completed TRIPOD-AI checklist is provided in Supplementary File S2. The machine learning workflow is illustrated in Figure 1 [59].

#### 2.11.1. Stability and Confidence Interval Analysis

To assess model stability, the average, standard deviation, and 95% confidence intervals of key performance metrics—accuracy, precision, sensitivity, specificity, F1-score, and AUC—were calculated based on the results of 100 iterations for the best-performing models, across both validation and test sets. This approach was used to quantitatively evaluate the robustness of each model to sample variability and to compare their generalizability.

To calculate the 95% confidence intervals, the distribution of each metric was first assessed using the Shapiro–Wilk test. For metrics that followed a normal distribution, classical parametric confidence intervals were computed using the formula in Equation (12). For non-normally distributed metrics, a bootstrap-based confidence interval estimation method was employed.

(12) $${CI}_{95\%}=\bar{x}\pm 1.96\times \frac{s}{\sqrt{n}}$$

Here, $\bar{x}$ represents the mean, s the standard deviation, and n the number of iterations. Based on this approach, models with narrower confidence intervals were considered to exhibit more stable classification performance [60].

#### 2.11.2. Statistical Analysis

All statistical analyses were conducted using IBM SPSS Statistics (Version 22.0, 64-bit). The distribution characteristics of continuous variables were evaluated using the Shapiro–Wilk test [61]. The performance metrics of the machine learning models on the test set—including accuracy, precision, recall, specificity, F1-score, and AUC—were analysed based on vectors obtained over 100 iterations. For normally distributed metrics, paired-sample *t*-tests were applied; for non-normally distributed metrics, the Wilcoxon signed-rank test was used. Parametric or non-parametric tests were selected for other continuous variables according to their distributional properties.

To assess differences between two independent groups, the Mann–Whitney U test was used for variables that did not show a normal distribution. In all analyses, a *p*-value < 0.05 was considered statistically significant.

To evaluate not only statistical significance but also the clinical effect size of group differences, Cohen’s d coefficient was calculated. This coefficient served as a descriptive measure to quantify the magnitude of observed differences [55].

Additionally, for features that showed high SHAP contribution and stability, those that also demonstrated statistically significant differences (*p* < 0.05) based on both Cohen’s d and the Mann–Whitney U test were further analysed using receiver operating characteristic (ROC) analysis [52,58]. This analysis aimed to assess the diagnostic performance of potential biomarkers for distinguishing PTVD (+) individuals. The area under the ROC curve (AUC), sensitivity, specificity, and the Youden index were calculated to determine clinically applicable optimal cutoff values.

### 2.12. Computational Environment

All data preprocessing steps, machine learning model training, and evaluation procedures were carried out using MATLAB R2023a and R2025a. The computations were performed on a personal workstation running a 64-bit Windows 11 (Education Edition) operating system, equipped with a 13th-generation Intel^®^ Core™ i9-13980HX processor (24 cores, 32 threads) and 32 GB of DDR5 RAM. Only the central processing unit (CPU) was used throughout the model training and evaluation phases; no graphics processing unit (GPU) was utilized.

### 2.13. Model Deployment Considerations

The machine learning model developed in this study was not tested within the integration framework of clinical Electronic Health Record (EHR) systems. However, considering the model architecture and processing time, no technical limitations were identified that would prevent its application in real-world clinical settings.

Based on the observed computational time and processing load during model training and testing, the following minimum system requirements are recommended for basic-level deployment:

- Quad-core CPU;
- 8 GB RAM;
- 256 GB SSD storage;
- 64-bit Windows 10 or 11 operating system.

For systems that do not meet these hardware specifications, cloud-based solutions (e.g., Microsoft Azure ML, Google Cloud AI Platform) may serve as viable alternatives. Due to its lightweight structure, the model can be further optimized for embedded use in healthcare information systems such as EHR platforms.

### 2.14. Data and Code Availability

The anonymized acoustic feature matrix used in this study (Dataset.xlsx, also provided as Supplementary File S29) and all associated analysis codes have been made publicly available on GitHub to ensure reproducibility: https://github.com/acousticStudy/Potential_Biomarkers_PTVD (created on 9 July 2025).

The shared dataset has been anonymized in accordance with the Turkish Personal Data Protection Law No. 6698 (KVKK) and does not contain any personally identifiable information.

### 2.15. Use of AI Tools

A generative AI tool (ChatGPT, GPT-4, OpenAI) was consulted during the preparation of the Materials and Methods Section to clarify methodological terminology and improve structural organization. Additionally, the same tool was used for language editing to enhance grammar, clarity, and readability throughout the manuscript. All content was critically reviewed and independently finalized by the authors.

## 3. Results

### 3.1. Patient Characteristics and PTVD Group Definition

A total of 126 patients who underwent thyroidectomy were included in this study. The mean age of the patients was 48.48 ± 11.66 years (47.85 ± 11.80 for females and 50.21 ± 11.10 for males). Among the participants, 26.98% were active smokers. Based on the type of surgical procedure, 11.11% of patients underwent Unilateral Total Thyroidectomy (UTT), while 88.89% underwent Bilateral Total Thyroidectomy (BTT) (Table 2).

To evaluate functional voice changes in patients, the Voice Handicap Index-10 (VHI-10) questionnaire was administered in both the preoperative and postoperative periods. The findings were analysed by sex and for the total sample.

As shown in Table 3, among female patients (*n* = 92), the mean preoperative VHI-10 score was 10.61 ± 5.19, which increased to 13.41 ± 5.13 in the postoperative period (*p* < 0.001). The proportion of female patients with a VHI-10 score ≥11 rose from 30.43% preoperatively to 43.48% postoperatively. Among male patients (*n* = 34), the mean preoperative VHI-10 score was 8.82 ± 3.82, which increased to 12.00 ± 4.63 postoperatively (*p* < 0.001). In this group, the proportion with a VHI-10 ≥ 11 rose from 20.59% to 29.41%.

When all cases were considered together (*n* = 126), the mean VHI-10 score increased from 10.11 ± 4.91 preoperatively to 13.02 ± 5.01 postoperatively (*p* < 0.001). Additionally, the proportion of patients with a VHI-10 ≥11 increased from 26.98% to 39.68%. However, correlation analysis revealed no statistically significant relationship between age and the increase in postoperative VHI-10 scores (Spearman’s rho = −0.016, *p* = 0.857).

Videolaryngostroboscopic evaluations conducted in both the preoperative and early postoperative period (4–6 weeks) did not identify any cases of recurrent laryngeal nerve (RLN) paralysis, cricothyroid dysfunction, or structural laryngeal pathology. Despite anatomically and neurologically normal laryngoscopic findings, the statistically significant increase in VHI scores supports the classification of this patient cohort as having functional voice disorders consistent with post-thyroidectomy voice disorder (PTVD). A VHI-10 cutoff score of ≥11 has previously been recommended for the diagnosis of functional dysphonia [22].

Despite anatomically and neurologically normal videolaryngostroboscopic findings, the significant increase observed in postoperative VHI-10 scores underscores the functional nature of PTVD in this cohort.

### 3.2. Outlier Analysis

To investigate potential outliers within the dataset, a z-score-based outlier analysis was conducted across all acoustic features. Data points exceeding ±3 standard deviations (|Z| > 3) were statistically considered outliers. Among the total of 17,136 data points, 274 entries (1.60%) met this criterion. However, considering that such values may reflect inter-individual physiological differences and potential pathological variations in voice data, it was decided not to exclude them from the analysis. This approach aimed to preserve the natural structure of the dataset and enhance the ability of machine learning models to recognize and generalize clinical heterogeneity. Accordingly, including outliers in the modelling process increased the representational power of real-world data variability.

### 3.3. Machine Learning Findings

The classification performance of the models was evaluated using the ROC AUC, accuracy, F1-score, precision, recall, and specificity metrics, calculated for both the validation set during training and a separate, unseen test set. Since the validation assessments were conducted using 3-fold cross-validation after feature selection, they reflect only the general trends observed during the training phase and are not intended for direct generalization. In this context, the results obtained from the test set are considered the primary indicators of the model’s actual performance.

#### 3.3.1. ROC-Based Model Comparisons

Among the SVM family, the Cubic SVM model with C = 0.1 achieved the highest mean AUC value on the test set (AUC: 78.07 ± 6.30; 95% CI: 76.79–79.19). However, this model exhibited poor performance on the validation set (AUC = 55.67 ± 7.02; 95% CI: 54.30–57.05). This discrepancy should be interpreted within the framework of the previously described validation strategy. In contrast, the Quadratic SVM (C = 0.1) model demonstrated higher AUC performance than other Quadratic SVM variants on both the validation and test sets (AUC: 75.29 ± 5.38 and 77.50 ± 6.40, respectively). Overall, SVM models with RBF kernels yielded lower AUC values compared to Cubic and Quadratic kernels (Supplementary Files S3 and S4).

The ROC curves for all three SVM kernels are presented in Supplementary File S5. The visual proximity and variance differences across the curves illustrate the performance inconsistencies between SVM models. Nevertheless, DeLong’s test did not reveal any statistically significant differences among the ROC curves (*p* > 0.05) (Supplementary Files S6–S8).

Among the Boosting models, the highest AUC on the test set was achieved by the GentleBoost model with NLC = 500 and LR = 0.01 (AUC: 81.23 ± 6.24; 95% CI: 79.98–82.40). This model also demonstrated consistently high performance on the validation set (AUC: 80.34 ± 2.51; 95% CI: 79.85–80.83) (Supplementary Files S3 and S4). Across all statistical comparisons with other GentleBoost and LogitBoost configurations, this model yielded significantly higher AUC values (*p* < 0.01) (Supplementary Files S9–S11). Although no statistically significant differences were found among the ROC curves of different GentleBoost configurations (*p* > 0.05), the ROC curve of the NLC = 500, LR = 0.01 configuration slightly diverged, visually highlighting its superior performance (Supplementary File S12).

Among the LogitBoost models, the highest AUC was observed in the LogitBoost model with NLC = 200 and LR = 1 (AUC: 80.40 ± 6.50) (Supplementary File S4), although this performance was statistically significantly lower than that of the best GentleBoost variant (*p* < 0.001) (Supplementary Files S9–S11). The ROC curves presented in Supplementary File S13 indicate considerable variation among LogitBoost models; however, DeLong’s test did not identify these differences as statistically significant (Supplementary Files S6–S8). Notably, the NLC = 500, LR = 0.01 LogitBoost variant exhibited a lower AUC value (77.58 ± 6.95) (Supplementary File S4), suggesting more complex interactions between the learning rate and the number of learning cycles in this model family.

In conclusion, among all evaluated models, the GentleBoost model with NLC = 500 and LR = 0.01 achieved the highest AUC values in both the validation and test sets, with a narrow confidence interval. The large area under the ROC curve highlights the model’s strong discriminative ability. Therefore, this model stands out as the most stable and generalizable classifier.

#### 3.3.2. Accuracy-Based Model Comparisons

Among the Boosting models, the highest test accuracy was observed with the GentleBoost model with NLC = 500 and LR = 0.01 (accuracy: 73.24 ± 6.43; 95% CI: 71.96–74.48) (Supplementary File S4). This model also achieved high performance on the validation set (accuracy: 73.21 ± 2.51; 95% CI: 72.65–73.77) (Supplementary File S3). The consistency between the validation and test results indicates that the model exhibits balanced and stable classification performance not only on the training data but also on unseen data. According to statistical analyses, this model achieved significantly higher accuracy compared to several other Boosting configurations (e.g., LogitBoost with NLC = 200 and LR = 0.05, *p* < 0.001) (Supplementary Files S14–S16).

Within the SVM family, the Cubic SVM model with C = 1 showed the highest test set accuracy (accuracy: 70.84 ± 6.24; 95% CI: 69.62–72.06). However, its validation accuracy was substantially lower (52.83 ± 11.59; 95% CI: 51.56–54.12), indicating a noticeable gap between training and generalization performance. Similar discrepancies between the validation and test scores were observed across RBF and Quadratic SVM variants as well (Supplementary Files S3 and S4). As stated earlier, these differences are attributed to the characteristics of the validation procedure, and thus, the test set results are prioritized when evaluating true generalization performance.

In summary, the GentleBoost model with NLC = 500 and LR = 0.01 emerged as the strongest classifier in terms of accuracy, achieving consistently high performance across both validation and test sets. The model’s generalizability and training stability are clearly supported by its accuracy metrics.

#### 3.3.3. F1-Score-Based Model Comparisons

Among the Boosting models, the highest test F1-score was achieved by the GentleBoost model with NLC = 500 and LR = 0.01 (F1-score: 72.97 ± 6.79; 95% CI: 71.62–74.33). This model also yielded the highest mean F1-score on the validation set (F1-score: 72.79 ± 5.57; 95% CI: 72.12–73.40) (Supplementary Files S3 and S4). The consistency between the validation and test data highlights the model’s generalizability and stability. Statistical comparisons across different GentleBoost and LogitBoost configurations revealed that this model demonstrated a significantly superior F1-score performance (*p* < 0.01) (Supplementary Files S17–S19).

Within the SVM family, the highest F1-score on the test set was observed with the RBF SVM model with C = 1 (F1-score: 70.53 ± 6.15; 95% CI: 69.32–71.73). However, this model performed more poorly on the validation set with an average F1-score of only 56.61 ± 7.74 (95% CI: 55.66–57.52). Cubic SVM models exhibited moderate performance on the test set (e.g., Cubic SVM with C = 1 yielded an F1-score of 70.03 ± 6.65) but generally produced low scores on the validation set (e.g., Cubic SVM with C = 1 yielded 49.27 ± 18.29) (Supplementary Files S3 and S4). As previously stated, these differences are attributed to the validation methodology and do not reflect the models’ actual generalization capability, which is primarily assessed based on the test set results.

Overall, the GentleBoost model with NLC = 500 and LR = 0.01 provided consistently high F1-scores on both the validation and test sets, emerging as the most robust and effective solution in the presence of class imbalance. This model demonstrated superior performance not only in terms of accuracy and the AUC but also in its ability to balance sensitivity and specificity, thereby enhancing class separability.

#### 3.3.4. Precision-Based Model Comparisons

Among the Boosting models, the highest test precision was observed with the GentleBoost model with NLC = 500 and LR = 0.01 (precision: 73.90 ± 7.27; 95% CI: 72.47–75.32). This model also demonstrated similarly high performance on the validation set (precision: 73.99 ± 6.30; 95% CI: 73.28–74.70) (Supplementary Files S3 and S4). The consistency between the training and test datasets indicates the model’s strong generalizability. Statistical comparisons revealed that this model had significantly higher precision than some other Boosting configurations (e.g., LogitBoost with NLC = 200 and LR = 0.05, *p* < 0.001) (Supplementary Files S20–S22).

Within the SVM family, the model with the highest test precision was the Quadratic SVM with C = 0.01 (precision: 86.00 ± 12.71; 95% CI: 83.44–88.49). However, its precision on the validation set was considerably lower at 68.08 ± 7.22 (95% CI: 67.26–68.90). Similarly, while the Cubic SVM (C = 0.01) model achieved high precision on the test set (83.87 ± 10.11), it performed substantially worse on the validation set (55.21 ± 15.34) (Supplementary Files S3 and S4). These discrepancies should be interpreted within the context of the validation methodology outlined earlier.

Overall, in terms of precision, the GentleBoost model with NLC = 500 and LR = 0.01 produced stable and balanced results across both the training and test datasets. Moreover, it emerged as the most successful Boosting algorithm with statistically significant superiority (Supplementary Files S20–S22).

#### 3.3.5. Recall-Based Model Comparisons

Among the Boosting models, the highest average recall on the test set was observed with the LogitBoost model (NLC = 200, LR = 1), achieving a recall of 73.80 ± 9.57 (95% CI: 71.92–75.74). This was followed by the GentleBoost model (NLC = 500, LR = 0.01), which yielded a recall of 72.76 ± 9.53 (95% CI: 70.86–74.72). The corresponding recall values on the validation set were 81.72 ± 7.06 and 72.32 ± 8.39, respectively. A notable consistency was observed between the training and test set performances (Supplementary Files S3 and S4), highlighting the strong generalizability of the Boosting models. Specifically, the LogitBoost (NLC = 200, LR = 1) configuration demonstrated statistically significantly higher recall compared to certain other Boosting configurations (e.g., LogitBoost NLC = 200, LR = 0.1; *p* < 0.01) (Supplementary Files S23–S25).

Within the SVM family, the RBF SVM model (C = 0.1) achieved the highest recall on the test set (recall: 79.92 ± 8.26; 95% CI: 78.44–81.56). However, it exhibited a markedly lower recall on the validation set (17.46 ± 27.73; 95% CI: 14.50–20.79). Similarly, both Cubic and Quadratic SVM models yielded lower recall scores on the validation set compared to the test set (e.g., Cubic SVM C = 1: validation recall = 51.06 ± 23.30 vs. test recall = 68.52 ± 8.82) (Supplementary Files S3 and S4). These discrepancies are attributed to the 3-fold cross-validation structure applied after feature selection and are not indicative of true model generalization, which is more accurately assessed using the test set.

In conclusion, the LogitBoost model (NLC = 200, LR = 1) demonstrated consistently high recall on both the validation and test sets, making it the most robust model in terms of identifying positive cases without omission.

#### 3.3.6. Specificity-Based Model Comparisons

Within the SVM family, the highest specificity was observed with the Quadratic SVM model (C = 0.01), with a test set specificity of 95.04 ± 4.41 (95% CI: 94.16–95.90). However, this model showed a decrease in performance on the validation set, yielding a specificity of 70.50 ± 10.03 (95% CI: 69.34–71.67). Similarly, the Cubic SVM model (C = 0.01) achieved a high specificity on the test set (92.40 ± 5.67; 95% CI: 91.20–93.52), yet its performance declined on the validation set (55.63 ± 24.15). The high specificity and narrow confidence intervals observed for the test set indicate that these models were effective in correctly identifying the negative class. However, such high specificity often came at the cost of reduced recall. For instance, the Quadratic SVM (C = 0.01) model had a test set recall of only 30.88 ± 10.65, indicating limited ability to detect positive cases (Supplementary Files S3 and S4).

Among the Boosting models, the highest specificity was achieved by the GentleBoost model (NLC = 500, LR = 0.01), with a test set specificity of 73.72 ± 9.38 (95% CI: 71.88–75.32). The model also demonstrated high specificity on the validation set (73.97 ± 8.57; 95% CI: 72.99–74.97) (Supplementary Files S3 and S4). Statistically significant differences were observed when compared to certain other Boosting configurations (e.g., compared to LogitBoost NLC = 200, LR = 1; *p* < 0.001) (Supplementary Files S26–S28). Notably, GentleBoost (NLC = 500, LR = 0.01) emerged as the only configuration that achieved both high recall and high specificity simultaneously.

In summary, while the Quadratic SVM (C = 0.01) model stands out for maximizing specificity alone, the GentleBoost (NLC = 500, LR = 0.01) model is the most suitable choice for a balanced classification system. Its ability to provide both high specificity and high recall, along with consistent performance across validation and test sets, makes it the most reliable model for accurate identification of the negative class.

#### 3.3.7. Confusion Matrix-Based Model Comparisons

Within the SVM family, the RBF SVM models with C = 0.1 and C = 0.01 achieved the highest number of true positives (TP = 20). However, these models also exhibited relatively high numbers of false negatives (FN = 12 and FN = 11, respectively) and false positives (FP = 5). The RBF SVM model with C = 1 presented a more balanced structure, yielding TP = 18 and FP = 7. In contrast, the Cubic SVM (C = 0.01) and Quadratic SVM (C = 0.01) models showed notably high false positive rates (FP = 15 and FP = 17, respectively), indicating imbalanced classification between the classes (Supplementary File S4).

Among Boosting-based models, all configurations of the GentleBoost family produced similar results. Notably, the GentleBoost model (NLC = 500, LR = 0.01) achieved a balanced performance with TP = 18, FP = 7, TN = 18, and FN = 7. The LogitBoost model (NLC = 200, LR = 1) yielded identical values, highlighting its competitive performance (Supplementary File S4).

In summary, while the RBF SVM models with C = 0.1 and C = 0.01 delivered the highest number of true positives, the GentleBoost and LogitBoost models exhibited more balanced and consistent confusion matrix profiles. This indicates that some models are more sensitive to the positive class, whereas others provide a more even performance across both classes (Supplementary File S4).

#### 3.3.8. Model Explainability: SHAP-Based Feature Evaluation

To enhance model interpretability and identify the features that contribute most to classification performance, SHAP (SHapley Additive exPlanations) analysis was applied, following the unified framework introduced by Lundberg and Lee [50,51]. Kernel SHAP was used for SVM-based models, whereas tree SHAP was implemented for Boosting-based models.

**Figure 2:**
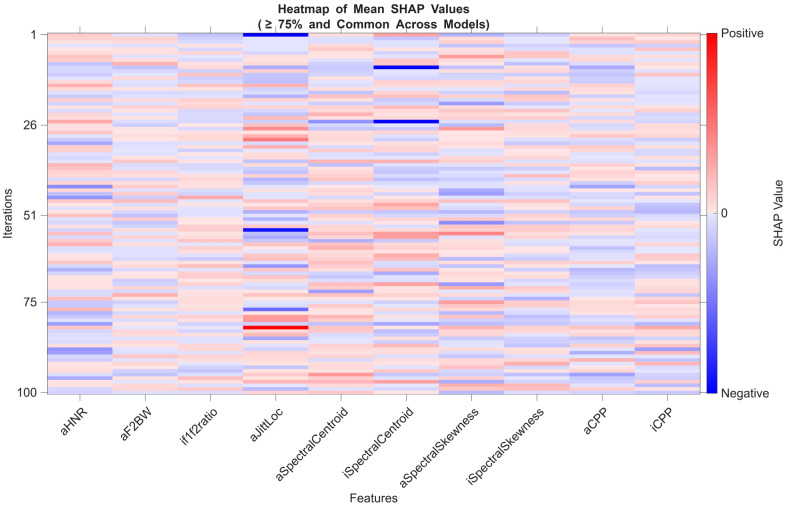
Heatmap of most frequently contributing features across all configurations.

The heatmap constructed using the average SHAP values of features selected in at least 75% of all models offers a comprehensive view of the most frequently contributing features across all configurations (Figure 2).

However, to identify robust biomarker candidates, the analysis was restricted to high-performing models (AUC ≥ 0.80). Among these, features that demonstrated consistent SHAP values in both direction and magnitude across training and test sets were defined as “stable biomarkers.” In this context, the features iCPP, aCPP, and aHNR exhibited reproducible contribution patterns in high-performing models. This suggests that these variables are reliable indicators, independent of both the model type and data partitioning.

**Figure 3:**
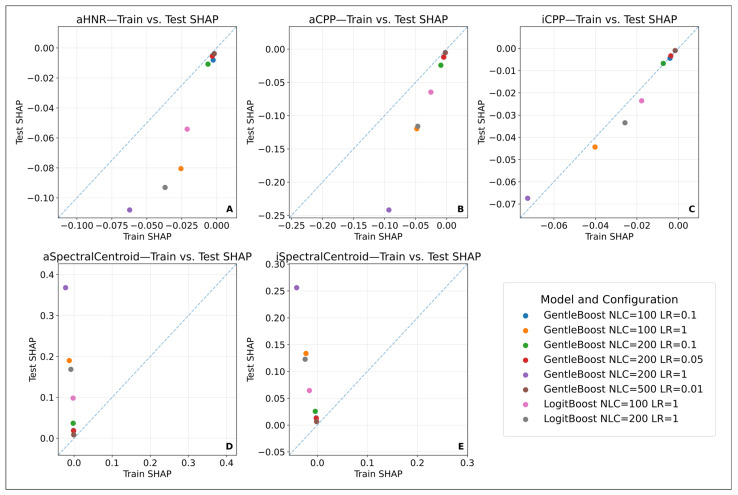
Comparison of SHAP contributions between training and test sets for five prominent acoustic features. (**A**) SHAP value distribution scatter plot for aHNR showing close alignment of points to the diagonal, indicating similar contribution levels in both training and test sets; (**B**) SHAP value distribution scatter plot for aCPP showing close alignment of points to the diagonal, indicating similar contribution levels in both training and test sets; (**C**) SHAP value distribution scatter plot for iCPP showing close alignment of points to the diagonal, indicating similar contribution levels in both training and test sets; (**D**) SHAP value distribution scatter plot for aSpectralCentroid showing noticeable variance in the direction and magnitude of SHAP values, suggesting limited generalizability; (**E**) SHAP value distribution scatter plot for iSpectralCentroid showing noticeable variance in the direction and magnitude of SHAP values, suggesting limited generalizability.

This finding is also visually supported by the SHAP value distribution scatter plots comparing the training and test sets. Specifically, the close alignment of points to the diagonal for iCPP (Panel C), aCPP (Panel B), and aHNR (Panel A) indicates similar contribution levels in both datasets. In contrast, variables such as aSpectralCentroid (Panel D) and iSpectralCentroid (Panel E) showed less consistency, with noticeable variance in the direction and magnitude of their SHAP values—suggesting limited generalizability (Figure 3).

In conclusion, SHAP analysis was conducted based on both the frequency of feature contributions across all models and the stability of these contributions in high-performance models. The features iCPP, aCPP, and aHNR were identified as potential stable biomarker candidates. This approach highlights the importance of evaluating not only feature frequency but also model consistency and independence from data splits in machine learning-based biomarker discovery.

#### 3.3.9. Diagnostic Utility of SHAP-Based Features in PTVD Identification

The most stable features identified through SHAP analysis—iCPP, aCPP, and aHNR—were evaluated for their statistical and diagnostic relevance between individuals with and without a PTVD diagnosis (Table 4).

Firstly, all three features exhibited negative Cohen’s d values, indicating that individuals with PTVD systematically demonstrated lower values for these acoustic parameters. The largest effect size was observed for iCPP (Cohen’s d = −2.95), which corresponds to a “very large” effect. aCPP showed a “large” effect (d = −1.13), while aHNR displayed a “medium” effect size (d = −0.60).

To complement the descriptive statistics and test group-level differences in a non-parametric manner, the Mann–Whitney U test was applied. The analysis revealed that individuals with PTVD had significantly lower values for iCPP (U = 189.000, Z = −6.137, *p* < 0.001), aCPP (U = 630.000, Z = −3.303, *p* = 0.001), and aHNR (U = 784.000, Z = −2.313, *p* = 0.021).

The mean rank values further supported these differences: individuals without PTVD had higher mean ranks—72.68 for iCPP, 68.44 for aCPP, and 66.96 for aHNR—while the PTVD group showed substantially lower mean ranks (20.09, 40.14, and 47.14, respectively).

These findings demonstrate that the discriminative power of these features is not only statistically significant in terms of effect size but also in distributional terms, reinforcing their diagnostic value in distinguishing PTVD from non-PTVD cases.

**Figure 4:**
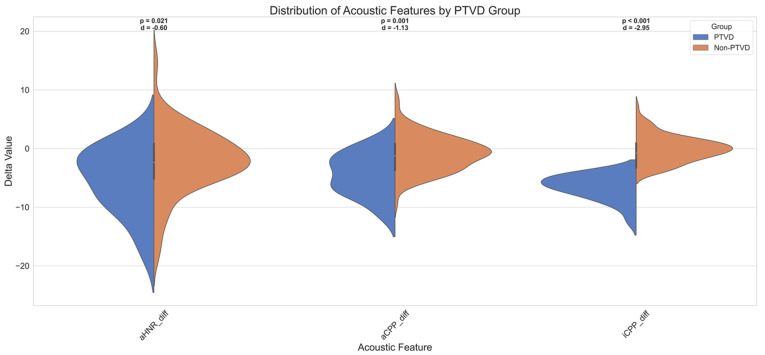
Violin plots of key SHAP-based acoustic features (iCPP, aCPP, aHNR) by PTVD group. PTVD participants show notably reduced delta values in iCPP and aCPP compared to non-PTVD participants. Each plot includes the *p*-value and Cohen’s d effect size annotation above the violins.

Figure 4 further visualizes these distributional differences using violin plots for the three prominent acoustic features—iCPP, aCPP, and aHNR. Each violin plot displays the data split by PTVD status, along with the corresponding *p*-value and Cohen’s d annotated above. These visuals clearly illustrate that participants with PTVD exhibited markedly lower delta values in iCPP and aCPP, further validating the statistical results shown in Table 4.

During the ROC analyses, considering these negative directional differences, all features were reverse-scored (i.e., modelled as y_score = −X), and AUC, optimal cutoff, sensitivity, and specificity values were calculated accordingly. According to the obtained findings, iCPP, with an AUC of 0.66, has a moderate discriminative power and stands out particularly with its high specificity (98%). This indicates high accuracy in excluding individuals without PTVD. On the other hand, although aCPP has a similar AUC value (0.64), it plays a strong role in correctly diagnosing individuals with PTVD due to its high sensitivity (89%). aHNR, with an AUC of 0.59, shows limited discriminative power.

These results reveal that iCPP and aCPP may be complementary diagnostic biomarkers, as one provides high specificity and the other provides high sensitivity. These findings support studies in the literature suggesting that the CPP and HNR parameters may show significant changes in functional voice disorders. In addition, the fact that these features, determined by the SHAP-based explainability approach, show meaningful performance not only in models but also in direct clinical diagnosis strengthens the methodological integrity.

When evaluated together with the defined effect sizes, post hoc power analyses support the reliability of the statistical significances. For the iCPP feature, the achieved 100% power (1.00), when considered with the very large observed effect size (Cohen’s d = 2.95), shows that the difference was detected with high confidence. Similarly, the 99.8% test power (power = 0.998) calculated for the aCPP feature, in line with the large effect size (Cohen’s d = 1.13), reveals that the statistical difference is significant and reliable. On the other hand, the 71.8% test power obtained for aHNR, with a moderate effect size (Cohen’s d = 0.60), is largely sufficient for detecting the difference but suggests that increasing the sample size in the future would be necessary for stronger confirmation of this finding.

## 4. Discussion

### 4.1. Diagnostic Challenges of PTVD and Acoustic–Machine Learning Approaches

Thyroid surgery is one of the most commonly performed endocrine procedures worldwide. Although voice changes are reported in a significant proportion of patients, in some cases, no apparent symptoms are observed. This situation can be explained by physiological mechanisms such as contralateral cord compensation. Post-thyroidectomy voice disorder (PTVD) is a functional dysphonia that occurs without recurrent laryngeal nerve (RLN) or external branch of the superior laryngeal nerve (EBSLN) injury and is laryngoscopically normal but presents with subjective voice complaints. Kim et al. [62] drew attention to the presence of patients reporting persistent voice symptoms for two years following thyroidectomy and emphasized that this condition should be evaluated at a functional rather than structural level.

Similarly, Sahoo et al. [63] reported significant changes in acoustic parameters such as *f*_0_ and shimmer in early voice analysis of cases in which the integrity of the RLN and EBSLN was preserved. In addition, Choi et al. [2] demonstrated in their cepstral analysis-based evaluations that changes in the CPP may be a sensitive marker for early diagnosis of PTVD. In our study as well, similar acoustic changes were observed in cases where anatomical nerve integrity was preserved, and these findings support that PTVD should be addressed not only structurally but also functionally and at a subclinical level.

Singh et al. [64] reported a significant decrease in *f*_0_ and an increase in pitch sigma on postoperative day 1 in patients who underwent thyroidectomy due to benign thyroid nodules; these changes were shown to resolve by day 7. No differences were observed in the jitter, shimmer, or HNR parameters. The findings indicate that temporary functional impairments may occur despite the anatomically preserved EBSLN. Similarly, in our study, acoustic changes were observed in cases with preserved nerve integrity, supporting the functional aspect of PTVD.

PTVD is typically diagnosed using self-reported questionnaires such as the VHI-10, which reflect the perception of voice but are limited in detecting subtle changes at the biophysical level [23,65]. The systematic review by Lang et al. [66] revealed that significant deterioration in acoustic parameters can occur even after uncomplicated thyroidectomy. These findings support the need for objective, reproducible, and non-invasive methods for early diagnosis.

Preoperative and postoperative systematic laryngeal examination is important in this context and is recommended by international guidelines such as those of the ATA, AAO-HNS, INMSG, and BTA [18,67,68,69]. However, laryngoscopy may be insufficient in detecting subclinical EBSLN injuries; videostroboscopy, on the other hand, has limited use due to cost and the requirement for specialized expertise. Although fibre-optic nasolaryngoscopy offers a more feasible alternative [70], the complementary role of objective methods such as acoustic analysis is increasingly emphasized due to the limitations of classical approaches in the evaluation of functional dysphonias [65].

The diagnostic challenge of PTVD arises not only from its reliance on subjective symptoms but also from the inability of these classical methods to adequately detect subclinical variants [65]. In particular, EBSLN injuries are often overlooked due to compensatory mechanisms [3], while laryngeal electromyography (L-EMG) has limited clinical utility due to its invasive nature and its value being restricted to late-stage diagnosis [4,71]. Therefore, in cases where no structural pathology is detected, a postoperative increase in VHI-10 scores may be considered a clinically meaningful indicator of functional PTVD.

PTVD is characterized by symptoms such as an inability to produce high-pitched sounds, vocal fatigue, throat discomfort, and deterioration in voice quality [72]. During the diagnostic process, a postoperative increase in VHI scores despite normal laryngoscopic findings plays a decisive role, and the diagnostic value of this increase is clearly defined in the literature [2,23,73]. The study by Vicente et al. [74] demonstrated that significant deterioration in voice quality may be observed after thyroidectomy even in patients without nerve injury. This supports the notion that PTVD may be associated with non-structural, functionally based changes. Additionally, in our study, the more pronounced increase in VHI scores among female participants highlights the possibility of gender-based sensitivity to postoperative voice changes (Table 3) [2].

In statistical analyses, no significant association was found between PTVD diagnosis and age, sex, pathological diagnosis, or type of surgery (*p* > 0.05). However, active smoking was found to be significantly associated with PTVD diagnosis (*p* = 0.032) (Table 2). This finding can be explained by the irritative effects of smoking on the vocal fold mucosa and phonation, as also noted in previous studies. Furthermore, it has been reported that chronic effects of tobacco products have notable impacts on voice quality in cases of functional dysphonia [75]. Accordingly, cigarette smoking may be considered an environmental risk factor contributing to the development of PTVD.

In the correlation analysis, no statistically significant relationship was found between age and the increase in the VHI-10 score (postoperative–preoperative difference) (Spearman’s rho = −0.016, *p* = 0.857). This finding indicates that perceived voice changes in the postoperative period are independent of age and that the development of PTVD cannot be explained solely by chronological age. This supports the notion that PTVD has a multifactorial structure arising from multiple factors such as individual vocal sensitivity, environmental influences, and compensatory mechanisms.

The limitations of classical methods highlight the need for advanced analytical techniques in the diagnosis of multifactorial functional disorders such as PTVD. In this context, machine learning models capable of evaluating high-dimensional and nonlinear patterns offer significant contributions not only in terms of classification performance but also in the early detection of subclinical changes and the support of individualized treatment decisions [8,9].

### 4.2. Classification Performance of Machine Learning Models

In this study, two main families of models—Support Vector Machines (SVMs) and Boosting algorithms—were evaluated for PTVD classification using acoustic features extracted from preoperative and postoperative voice recordings. Within the SVM family, Cubic, Quadratic, and RBF kernel structures were tested, while the Boosting family included GentleBoost and LogitBoost algorithms, each examined under various parameter combinations. Model performances were assessed in two stages: on the validation set obtained after feature selection and on a completely independent test set that the model had not previously seen (Supplementary Files S3 and S4). This distinction is important, as the validation set reflects trends related only to the training process, whereas only the test set scores should be considered when assessing true generalizability [76,77]. This approach also aligns with model reporting standards such as TRIPOD-AI (Supplementary File S2).

In terms of the ROC AUC, the GentleBoost model (NLC = 500, LR = 0.01) provided the highest and most consistent AUC values across both the validation set (80.34 ± 2.51) and the test set (81.23 ± 6.24). This indicates not only a high discriminative capacity but also strong generalizability between the training and testing phases. While graphical differences between ROC curves visually supported distinctions among models, the DeLong test assessed the statistical significance of these differences, with no significant difference observed (*p* > 0.05) (Supplementary Files S6–S8). Nonetheless, the narrow confidence intervals and high stability of the GentleBoost model were found to be noteworthy.

Overall, the combination of a small learning rate with a high number of learning cycles in Boosting algorithms stabilizes the decision boundary and controls model variance, thereby enhancing biological interpretability. This configuration offers a critical advantage, particularly in cases involving subclinical and complex acoustic patterns such as PTVD.

Other performance metrics such as accuracy, F1-score, precision, and specificity showed similar trends. The GentleBoost model provided high generalizability by achieving comparable accuracy scores in the validation and test sets (73.21% and 73.24%, respectively) while also yielding the highest F1-score (72.97 ± 6.79) and precision (73.90 ± 7.27). Moreover, it maintained a balance between recall (72.76 ± 9.53) and specificity (73.72 ± 9.38), demonstrating bilateral performance, which is crucial for clinical applications (Supplementary Files S3 and S4). This indicates that the model possesses consistent discriminative power across all classes, surpassing the imbalanced performances that focus solely on a single class.

Considering that, in a rare clinical condition such as PTVD, minimizing not only false positives but also false negatives is essential, high F1-score values reveal the overall effectiveness of the model. The high F1-score achieved by the GentleBoost model on the test set (72.97 ± 6.79) indicates that it accurately identifies positive cases while maintaining a low rate of misclassification. Furthermore, the consistency of the F1-score across the validation and test sets shows that the model offers generalizable performance not only on the training data but also on previously unseen data. From this perspective, the F1-score is not merely a performance metric but also an indicator of the model’s stability in practical clinical decision support processes.

Statistical comparisons of the F1-scores among the models are presented in Supplementary Files S17–S19. Notably, the GentleBoost model (NLC = 500, LR = 0.01) achieved a significantly higher F1-score compared to models such as Cubic SVM (C = 0.1) (*p* < 0.05), with a difference exceeding 5%. These results indicate that the model is superior not only in terms of overall accuracy but also in terms of class balance.

Considering that false positives in PTVD diagnosis may lead to unnecessary interventions, the high precision score of GentleBoost (73.90 ± 7.27) is particularly noteworthy. This demonstrates that the model is highly selective in its positive class predictions and minimizes the false positive rate. A high precision value can enhance clinical effectiveness by reducing misdirection in further assessments or treatment protocols following initial screening tests. Moreover, the consistency of the precision value between the test and validation sets indicates that this performance is not limited to the training data but can be generalized to the broader patient population. Thus, the GentleBoost model provides a balanced classification approach with both high sensitivity and high specificity.

Statistical comparisons of the precision values among the models are presented in Supplementary Files S20–S22. The GentleBoost model (NLC = 500, LR = 0.01) achieved significantly higher precision scores compared to models such as Quadratic SVM (C = 0.01) (*p* < 0.05), with the difference exceeding 10% in some comparisons. This finding indicates that the model provides high accuracy in positive classifications and minimizes the false positive rate.

In clinically subjective conditions such as PTVD, the overall accuracy of the model serves as an important initial evaluation criterion. The high accuracy score obtained by GentleBoost on the test set (73.24%) is noteworthy in this regard. The model’s similar success rates in both positive and negative classes go beyond misleading performances focused on a single class, ensuring overall classification balance. Furthermore, the close alignment of accuracy values between the validation and test sets (73.14% vs. 73.24%) demonstrates the model’s applicability to the general patient population. This consistency is a key indicator of the model’s reliability, especially in clinical decision support systems.

The statistical significance of the differences between model accuracy values is presented in Supplementary File S15; this analysis shows that GentleBoost algorithms demonstrate a statistically significant superiority over certain SVM models (*p* < 0.05). The magnitude of these differences is detailed in Supplementary File S16, where it is observed that GentleBoost provides over 5% higher accuracy compared to models such as Cubic SVM (C = 0.1). All detailed statistical comparisons are provided in Supplementary File S14.

From the perspective of SVM models, although some kernel structures showed high scores in the test set, discrepancies between the validation and test sets are noteworthy. For instance, while the Cubic SVM (C = 0.1) model achieved a high ROC AUC on the test set (78.07 ± 6.30), its low AUC value on the validation set (55.67 ± 7.02) indicates an unstable performance during the training process. Similarly, although the Cubic SVM (C = 1) model demonstrated high test accuracy (70.84 ± 6.24), its validation accuracy was low (52.83 ± 11.59). Such discrepancies once again emphasize the importance of evaluating models primarily based on test set performance.

Additionally, the observation that validation scores are lower than test scores in some models (particularly within the SVM family) does not represent a classic case of overfitting; rather, it may be associated with limitations of the 3-fold cross-validation structure within the training set and sample variance. In this study, feature selection was performed solely on the training set, followed by stratified 3-fold cross-validation within the same training set. While this preserves class ratios, the resampling of the training data into subsets may further reduce sample size and diversity, potentially leading to lower performance for some models during validation. In contrast, the independent test set, which includes a more heterogeneous and larger sample, may better reveal the model’s overall learning capacity. Especially in margin-based algorithms such as SVM, small variations can excessively affect the decision boundary. Therefore, higher test scores compared to validation scores are considered a positive finding in terms of generalizability, and validation scores alone should not guide final conclusions [78].

The LogitBoost (NLC = 200, LR = 1) model achieved the highest average recall value in the test set (recall: 73.80 ± 9.57), standing out in terms of capturing the positive class. However, the specificity of this model was relatively low, indicating a limited capacity to distinguish negative classes. In contrast, the GentleBoost (NLC = 500, LR = 0.01) model provided both high recall and high specificity, offering a more balanced and reliable structure in terms of clinical validity (Supplementary Files S3 and S4). These findings are supported by bidirectional statistical tests related to recall and specificity metrics (Supplementary Files S23 and S26). Furthermore, the statistical significance of differences between models is visualized in Supplementary Files S24 and S27, and the effect sizes are detailed in the heatmaps presented in Supplementary Files S25 and S28. These additional findings further highlight the balanced performance of GentleBoost in terms of both sensitivity and specificity.

In the detection of rare but clinically significant conditions such as PTVD, models with high sensitivity (e.g., LogitBoost) may be preferred; however, when class balance is considered, GentleBoost appears to offer a more consistent approach. The confusion matrix analyses also support these results (Supplementary File S4). Although RBF SVM models achieved high TP values, relatively high FP and FN rates indicate a tendency toward imbalanced classification between classes. In contrast, GentleBoost and LogitBoost models demonstrated a more stable performance by achieving a balanced distribution in both TP and TN values. In particular, the GentleBoost model exhibited similar success rates in both positive and negative classes, increasing its suitability for use in clinical decision support systems. While some SVM models (e.g., Quadratic SVM C = 0.01) yielded high specificity values, their low recall suggests a risk of missing positive cases, which can be a limiting factor in scenarios where diagnostic sensitivity is critical.

In conclusion, based on multi-faceted evaluations including the ROC AUC, accuracy, F1-score, precision, recall, and specificity, the GentleBoost algorithm emerged as the most successful model in the study with its balanced performance across classes, generalizability, and high explainability potential. As noted in the literature [79], the sequential learning capacity of Boosting algorithms enhances classification performance, particularly in high-dimensional and imbalanced datasets. Moreover, this model was supported by SHAP contribution analyses, offering not only performance advantages but also strong explainability features that may contribute to clinical decision-making processes. Studies such as those by Molnar [80] and Ponce-Bobadilla et al. [81] emphasize the importance of SHAP-based explainability for clinical reliability and user acceptance.

In line with this approach, Uloza et al. [82] reported that their artificial intelligence algorithm named ASVI, developed for patients with a substitution voice, showed a strong correlation with expert-based IINFVo scores (r_s_ = 0.863, *p* < 0.001). This finding indicates that AI-based acoustic indices can potentially be integrated into clinical decision support systems, particularly for the objective classification of subclinical vocal changes.

In this context, our study can be considered one of the pioneering works integrating an explainable machine learning approach supported by SHAP visualizations into PTVD classification.

#### 4.2.1. The Effect of Small Sample Size and Modelling Strategies

The dataset used in this study consisted of a total of 252 voice recordings, with an equal distribution between the preoperative and postoperative groups (*n* = 126/126). However, this sample size is considered relatively small in the context of machine learning applications and may pose limiting effects on the generalizability of the model. In small sample sets, the risk of overfitting increases, particularly in high-parameter models; at the same time, model variance and statistical reliability may also be adversely affected. Raudys and Jain [83] emphasize that in small samples, classifier selection, feature dimensionality, and model complexity must be carefully optimized. In this context, LASSO-based feature reduction was applied in each iteration of the study, ensuring that models trained with limited data focused only on features with high information-carrying potential.

Another challenge posed by small sample size is the reliable evaluation of model performance. Therefore, a 3-fold cross-validation and a 100-iteration repetition strategy were employed in the study to reduce variance and to ensure that results were not affected by random sample splits. As emphasized by Kourou et al. [84], in small and imbalanced datasets, feature selection and appropriate model configuration play a critical role in classification success.

Accordingly, taking the dataset size into account, the classification algorithms used were also carefully configured. Support Vector Machines (SVMs), based on the principle of structural risk minimization, offer an advantage in generating generalizable decision boundaries in small datasets [85]. The Gaussian RBF and polynomial kernel functions used in this study were capable of modelling complex separations in the data space; automatic kernel scaling and constrained Box Constraint values were applied to reduce the risk of overfitting.

On the other hand, Boosting algorithms (GentleBoost, LogitBoost), while capable of producing low-bias and high-performance models, tend to exhibit high variance due to their sequential learning nature. In this study, specific configurations were applied to balance this variance tendency and to prevent model overfitting. The maximum number of splits for weak learners was limited to 10; pruning and leaf merging operations were disabled. Additionally, to maintain balance during learning, the Minimum Parent Size was set to 6 and the Minimum Leaf Size to 3. These values aimed to prevent the decision tree from branching without sufficient sampling, thereby enhancing model generalizability. In addition to these configurations, different learning rates were also systematically tested to optimize the adaptability of Boosting algorithms to small sample sets [83,84].

In conclusion, despite the small sample size, the models were configured in a data-sensitive manner, and generalizable and reliable classification performance was achieved through feature selection, cross-validation, and hyperparameter control.

#### 4.2.2. Model Configurations and the Bias–Variance Trade-Off

The configuration of classification algorithms used in this study was carefully implemented, taking into account the dataset size, structure, and the nature of the classification problem (Table 1). In particular, the effect of selected hyperparameter settings on the bias–variance trade-off of the models deserves discussion in this section.

Hyperparameter configurations not only define the model architecture but also play a critical role in controlling the bias–variance balance of classification models.

The SVM models employed in this study (with Quadratic, Cubic, and Gaussian RBF kernels) are generally characterized by low variance and moderate bias in high-dimensional and small-sample datasets. Specifically, automatic scaling of the kernel function, systematic exploration of Box Constraint (C) values, and careful selection of kernel types (polynomial d = 2, 3, and Gaussian RBF) are key determinants of model flexibility. Polynomial kernel SVMs can reduce bias by defining more complex decision boundaries, whereas high C values may increase variance. Gaussian RBF kernel SVMs typically offer low bias and controlled variance, thus providing strong generalizability. This behaviour is directly associated with Vapnik’s structural risk minimization theory [85] and is further supported by recent empirical comparisons [86].

On the other hand, Boosting-based algorithms (GentleBoost and LogitBoost) tend to achieve low bias by minimizing errors through sequential learners; however, this may increase the risk of overfitting, especially with limited sample sizes. To balance this risk, the maximum number of splits for each weak learner was limited to 10, and pruning and leaf merging operations were disabled. This approach allowed the learners to flexibly capture patterns while keeping overall complexity under control. In addition, systematic testing of different learning rates (1, 0.1, 0.05, 0.01) was performed to further reinforce variance control. This configuration aligns with Friedman’s theoretical framework on Boosting [87] and has been successfully demonstrated in modern bias–variance analyses [86].

In conclusion, the hyperparameter adjustments made in both SVM and Boosting models were configured in line with the characteristic bias–variance tendencies of each algorithm family, thereby maintaining a balance between generalizability and the risk of overfitting.

#### 4.2.3. Eliminating Randomness and Ensuring Model Consistency

In this study, all models were trained and tested using a fixed random seed. This approach enabled the evaluation of different hyperparameter configurations independent of randomness stemming from data splitting. In some models, despite changes in hyperparameters, the AUC values remained the same, which may be attributed to the model’s learning capacity reaching early saturation or the limited impact of the parameter changes. This is not the result of a coding or methodological error but rather a natural consequence of the structural behavioural characteristics of the model.

Furthermore, when explainable artificial intelligence (XAI) techniques such as SHAP are employed, it has been observed that even models with identical AUC values can exhibit different feature contribution profiles. This finding indicates that the assumption that models with similar classification performance possess similar decision-making mechanisms does not always hold true. Therefore, model evaluation should not rely solely on summary metrics (e.g., AUC) but should also be conducted in a multidimensional manner that considers the internal decision structure of the model.

#### 4.2.4. Areas of Agreement and Discrepancy Between the Findings and the Existing Literature

The performance metrics used in this study—such as the ROC AUC, F1-score, and accuracy—have enabled an objective evaluation of classification performance for PTVD. However, how these metrics should be interpreted within a clinical context is of particular importance in the literature. Recent studies propose appropriate methodological approaches for interpreting concepts such as threshold sensitivity and optimal decision points in ROC AUC analysis [52,58]. These references are especially instructive for interpreting biomarker accuracy and determining optimal cutoff values.

In our study, classification performance was presented using summary statistics such as the ROC AUC and F1-score. However, in diagnostic modelling studies, graphical representation of ROC curves is also essential, as it allows for a more accurate evaluation of the trade-off between sensitivity and specificity [80]. This approach particularly enhances visual intuitiveness in threshold-setting processes within clinical decision support systems. Additionally, methods such as the DeLong test are recommended for determining whether the differences in ROC AUC values between models are statistically significant [52,58].

In SHAP analyses, the contribution values can sometimes be extremely small (e.g., <10^−13^), which may hinder visual interpretation. Therefore, not only the magnitude of contributions but also the likelihood of features being repeatedly selected under different sampling conditions should be considered. Nogueira et al. [56] emphasized that stability should be assessed not only in terms of model contribution but also in terms of consistency across different data partitions.

In the literature, there are numerous machine learning-based modelling studies for the classification of dysphonic individuals. For example, Dankovičová et al. [9] reported 85% accuracy in general dysphonia diagnosis; Hadjaidji et al. [10] achieved 95% accuracy in classifying spasmodic dysphonia using the Random Forest algorithm and identified features such as jitter, the HNR, and MFCC as discriminative. Costantini et al. [12] reached an accuracy of 91.3%, with CPP and jitter being prominent across different vocal tasks. Similarly, Van der Woerd et al. [13] developed models that predicted GRBAS scores with high correlation levels (r = 0.78–0.84) using acoustic features such as shimmer and spectral slope.

While many dysphonia classification studies in the literature target prominent vocal pathologies or neurogenic disorders, the present study analyses a subclinical and microperturbative condition such as functional PTVD using preoperative and postoperative recordings of the same individual. This leads to the preservation of biometric constants that constitute vocal identity—such as *f*_0_, formant structure, and articulatory resonance—to a large extent [88]. This stability in the phonation system narrows the learning domain of classification algorithms and complicates diagnostic discrimination. Therefore, the observed accuracy rates stem not from a deficiency of the algorithms but from the inherently limited biological separability of PTVD [89,90]. This information from the literature explains the unique methodological challenges of classifying PTVD cases that develop after thyroidectomy using machine learning methods. Accordingly, the present study, which uses preoperative and postoperative recordings of the same patient, demonstrates lower classification performance compared to studies that classify clearly healthy versus dysphonic cases [9,10,12].

Most of these previous studies have focused on neurogenic or organic dysphonias. Machine learning applications specific to functional PTVD are quite limited. For example, Low et al. [11] reported 85% accuracy in UVFP classification using SHAP-based explainability analysis, yet functional PTVD cases were outside the scope of that model.

In this context, our study constitutes one of the pioneering works aimed at systematically classifying functional PTVD cases—developing without anatomical or neurological lesions—through machine learning. Compared to previous studies in the literature focusing on UVFP, spasmodic dysphonia, and neurogenic pathologies, this study addresses a significant gap by examining a patient group that is laryngoscopically normal yet reports subjective complaints [8,11,12].

Moreover, this study not only evaluates traditional acoustic features such as jitter, shimmer, and CPP but also comprehensively analyses advanced acoustic parameters including power distributions based on frequency bands, spectral density indicators (e.g., spectral centroid, skewness), and harmonic structure parameters (e.g., HNR, F2BW, f1f2ratio). Through SHAP analysis, the contributions of features commonly selected in over 75% of iterations and across multiple models to classification decisions were visually demonstrated. The resulting heatmap revealed that certain features—particularly iCPP, aCPP, aJittLoc, and iSpectralSkewness—provided stable and significant contributions to classification outcomes. Thus, the study ensured not only classification performance but also biological explainability and interpretability of potential biomarkers.

In conclusion, this study offers an original and multidimensional contribution to the literature on functional PTVD cases in terms of both model performance and explainability.

### 4.3. VHI-10 Correlation and Clinical Validation

The integration of subjective scales and objective acoustic parameters in the diagnostic evaluation of PTVD is of critical importance in terms of both clinical reliability and biomarker validation. The Voice Handicap Index-10 (VHI-10) is a widely used instrument that assesses patients’ self-perceived voice-related quality of life and has shown significant correlations with acoustic laboratory measures in various studies [91]. The validity and reliability of the Turkish version used in this study were previously confirmed by Kiliç et al. [24].

The studies by Choi et al. [2] and Kim et al. [92] have demonstrated that patients can perceive voice-related changes in the early period following thyroidectomy. These findings support that patients with functional PTVD may be able to detect subclinical changes in their voice and that such perceptions may align with objective measurements.

In our study, the acoustic features that stood out were not only decisive in model predictions but also consistent with patient-reported symptomatology. This suggests that these parameters can be considered as valid biomarker candidates for PTVD at both mathematical and clinical levels.

Moreover, the literature has shown that acoustic features play a crucial role not only in diagnostic validation but also in treatment planning. In particular, targeted voice therapies initiated within the first 2–8 weeks after thyroidectomy have been reported to enhance phonatory control, shorten the duration of dysphonia, and improve quality of life [2,71,92]. In this context, early acoustic evaluations may provide an objective reference point for determining which patients should be referred for voice therapy during the postoperative period. This approach offers advantages in strengthening clinical decision support systems and optimizing healthcare resource utilization.

In conclusion, the acoustic analysis and machine learning-based classification models developed in this study may serve as complementary diagnostic tools for identifying functional PTVD cases that cannot be detected through classical examination methods. Specifically, the GentleBoost and LogitBoost models may contribute to the timely referral of patients to voice therapy by objectively detecting subclinical voice changes in the early period [2,92]. Furthermore, prominent features identified through SHAP-based explainability analysis, such as CPP and the HNR, not only contribute to classification performance but also enhance the understanding of the biophysical mechanisms underlying PTVD.

Additionally, Yousef et al. [93] reported that CPPs provided more balanced sensitivity (65%) and specificity (78%) than AVQI-3 in screening for voice disorders and demonstrated a performance level comparable to some machine learning models. These results support the prominence of CPP in our study’s PTVD classification and suggest that acoustic biomarkers may offer a practical tool for clinical screening.

### 4.4. SHAP-Based Explainability and Potential Stable Acoustic Biomarker Candidates

For machine learning models to be adopted in clinical practice, it is not sufficient for them to offer high accuracy alone; their decision-making processes must also be interpretable. In this context, the SHAP methodology was applied in our study to ensure model explainability, enabling the identification of features contributing to classification performance as well as the assessment of their stability and clinical relevance [50,80].

SHAP analyses were performed using kernel SHAP for Support Vector Machines (SVMs) and tree SHAP for Boosting algorithms. A heatmap (Figure 2) was generated by considering only the features selected in at least 75% of all models, visually presenting the intensity of contribution from acoustic parameters. However, to define potential biomarker candidates, the analysis was limited to models with high discriminative power (AUC ≥ 0.80). This strategy focuses not only on the frequency of SHAP contributions but also on their reproducibility independent of data splitting and model configuration.

In evaluations conducted using this approach, certain features were observed to exhibit similar SHAP distribution patterns in both the training and test sets in terms of direction and magnitude. Notably, the features iCPP, aCPP, and aHNR demonstrated consistent SHAP values and were thus identified as “stable biomarkers.” In the corresponding distribution plots (Figure 3), data points densely aligned near the diagonal indicate model-independent consistency. In contrast, parameters such as aSpectralCentroid and iSpectralCentroid exhibited higher variance and were considered limited in terms of generalizability.

In SHAP analyses, some features in high-performing models (AUC ≥ 0.80) showed similar distribution patterns across training and test sets. However, SHAP correlation analyses conducted across all models and features revealed no statistically significant relationship between SHAP values in the training and test sets. This indicates that a generalized SHAP contribution pattern could not be established across all model structures. Similarly, in the study by Scheda and Diciotti [54], it was emphasized that the generalizability of SHAP explanations is only achievable through repeated validation strategies and high model performance; feature contributions may exhibit inconsistencies across different data splits. Therefore, stable biomarker identification was based solely on high-performing models and features that demonstrated visually consistent SHAP distributions.

The diagnostic validity of these three features identified via SHAP (iCPP, aCPP, aHNR) was further confirmed through separate statistical analyses. According to Cohen’s d effect size, a very large effect was observed for iCPP (–2.95), a large effect for aCPP (–1.13), and a moderate effect for aHNR (–0.60). The fact that these features consistently exhibited lower values in individuals diagnosed with PTVD indicates that they reflect the biophysical alterations specific to functional voice disorders. In addition, intergroup comparisons conducted using the Mann–Whitney U test also supported that these differences were statistically significant (*p* < 0.05) (Table 4).

Based on ROC analyses, the iCPP parameter stood out with 98% specificity (AUC = 0.66), while aCPP showed 89% sensitivity (AUC = 0.64). This distinct profile suggests that these two variables may serve as complementary diagnostic biomarkers. Although aHNR demonstrated more limited discriminative ability (74% specificity, AUC = 0.59), it may still play a supportive role in PTVD assessment. Post hoc power analyses indicated that the statistical differences, particularly for iCPP (power = 1.00) and aCPP (power = 0.998), were detected with high confidence. For example, a patient with a high-specificity iCPP value detected postoperatively (>−10) may be considered at low risk for PTVD, potentially avoiding unnecessary voice therapy or follow-up. Conversely, a patient with a low aCPP value (<−16.5) may be prioritized for proactive voice therapy due to its high sensitivity. Such explainability-based threshold values may be utilized in clinical decision support systems to develop personalized monitoring strategies.

Sampaio et al. [94] reported that CPP and spectral measurements in dysphonic individuals may be influenced by vocal intensity, fundamental frequency, and sample duration; however, they emphasized that CPP exhibits high sensitivity in detecting vocal fold dysfunction. Similarly, Low et al. [11] demonstrated through SHAP analysis that features such as shimmer and CPP provided systematic and clinically interpretable contributions to model decisions in the detection of vocal fold paralysis. These studies reveal that acoustic parameters contribute not only to classification performance but also to clinical reliability.

Biomarker studies using SHAP-based explainability analyses in functional dysphonias remain quite limited. The study by Gillespie et al. [91], which reported significant correlations between shimmer, jitter, and spectral features and the VHI-10 score, serves as a pioneering reference in this field. However, our present study, which evaluates functional PTVD using SHAP, offers a novel contribution to the literature by demonstrating that explainable artificial intelligence approaches may also be valid for subclinical dysphonias.

In conclusion, SHAP analysis not only explains the within-model contribution of features but also provides a powerful tool for identifying stable biomarkers and validating these features in terms of clinical relevance. The contribution of acoustic parameters such as iCPP, aCPP, and aHNR to both classification performance and diagnostic discriminability demonstrates the potential of explainable artificial intelligence-based approaches in functional voice disorders such as PTVD.

### 4.5. Clinical Implications of Stable Acoustic Biomarkers: Personalized Therapy and Early Monitoring

This study not only reveals the diagnostic potential of acoustic biomarkers but also emphasizes their clinical applicability in therapy planning and patient monitoring. Particularly in professions where voice plays a central role—such as teaching, broadcasting, and performing arts—even minor changes in voice quality can significantly impact professional performance and quality of life.

Recent studies have shown that post-thyroidectomy functional voice disorders can be effectively managed with structured therapy protocols, especially when initiated early in the postoperative period [20,92]. In addition, the Korean Society of Laryngology, Phoniatrics and Logopedics emphasizes the importance of early detection and intervention in their clinical practice guideline, recommending a comprehensive voice evaluation even in the absence of overt laryngeal pathology after thyroid surgery [95].

Therefore, biomarkers that reflect the biophysical characteristics of the voice and can objectively track change over time may play a critical role in early intervention and treatment customization. In this context, the stable biomarkers identified in our SHAP analysis—iCPP, aCPP, and aHNR—not only supported model performance but also exhibited directionally consistent differences in PTVD cases, consistently presenting with lower values.

Monitoring these features longitudinally may provide clinicians with insight into therapy responsiveness and inform decisions regarding therapy duration, intensity, and frequency. For instance, early improvement in CPP values may indicate good response and enable early cessation of therapy, whereas persistently low HNR levels might suggest the need for prolonged or more intensive intervention.

In current clinical practice, diagnosis of functional voice disorders still heavily relies on subjective reports and laryngoscopic evaluations, which can be prone to variability. By incorporating SHAP-based objective acoustic features into clinical workflows, a more standardized, reproducible, and personalized voice management strategy can be achieved. Zeng et al. [96] highlighted that patients often face delays in diagnosis and treatment due to the subtle nature of postoperative voice complaints—underscoring the need for accessible and objective assessment tools.

In summary, explainable acoustic biomarkers not only enhance diagnostic precision but also have the potential to revolutionize voice therapy by enabling early, individualized, and dynamic intervention planning. Future multi-centre studies are warranted to validate these findings and facilitate their integration into routine clinical guidelines.

## 5. Conclusions

This study fills a critical gap in the literature by integrating machine learning models—which offer high diagnostic accuracy in post-thyroidectomy vocal dysfunction (PTVD) but often remain “black boxes”—with an explainability-based approach. Functionally dysphonic cases that are laryngoscopically normal yet present with subjective vocal complaints are frequently overlooked in clinical practice, thereby limiting opportunities for early diagnosis and intervention. In this study, high-resolution acoustic analysis was combined with SHAP-based explainable machine learning algorithms to achieve not only high classification performance but also clinically interpretable model decisions. In particular, the features iCPP, aCPP, and aHNR consistently contributed across both training and test sets and were thus identified as stable biomarker candidates, aiding in both the diagnostic and biophysical understanding of PTVD. These features, which showed significant correlations with patient-reported outcomes such as the VHI-10, may serve as guidance not only for classification accuracy but also for personalized voice therapy strategies. Supported by robust methodological elements such as multiple algorithms, repeated cross-validation, and SHAP-based feature stability selection, the framework developed in this study constitutes a clinically valid, reproducible, and generalizable example of explainable AI-based diagnosis in voice disorders.

## 6. Strengths, Generalizability, Reproducibility, and Limitations

### 6.1. Strengths

In this study, to bridge the gap between data science and clinical accuracy, the prevention of data leakage, the establishment of statistically sound comparisons, explainability analyses, and the multi-layered validation of biomarkers were integratively structured from an engineering perspective.

All feature selection procedures were performed solely on the training set, while the test set was kept completely independent from this process. Thus, the model’s performance on test data reflects a data-leakage-free, genuinely generalizable evaluation.

For all classification models, metrics such as accuracy, sensitivity, specificity, the F1-score, and the ROC AUC were reported with 95% confidence intervals, thereby transparently revealing the statistical uncertainty of model predictions.

Prior to comparing performances across different model architectures, the Shapiro–Wilk test was applied to assess normality. For normally distributed metrics, the paired *t*-test was used, whereas the Wilcoxon signed-rank test was applied for non-normally distributed ones. The DeLong test was employed to evaluate the statistical significance between ROC curves, thereby maintaining the methodological validity of the statistical analyses.

Stable biomarker candidates were defined not only based on SHAP frequency but also as features (iCPP, aCPP, aHNR) that exhibited similar SHAP distribution patterns in terms of direction and magnitude across both training and test sets. This approach provides a consistency analysis independent of model architecture and data splitting.

Multi-layered tests were performed for diagnostic validation:

(1) After assessing normality, Cohen’s d was calculated to evaluate the effect size of the features;
(2) Between-group differences were analysed using the Mann–Whitney U test;
(3) Post hoc power analysis was conducted using G*Power software to assess the power of these tests.

This systematic, engineering-driven approach not only ensured a transparent and reproducible modelling process but also provided multi-level support for the clinical and mathematical validity of diagnostic biomarkers. This framework holds promise as a pioneering step toward the development of explainable AI-based decision support systems in functional voice disorders such as PTVD.

### 6.2. Generalizability

#### 6.2.1. Factors Supporting Generalizability

In this study, a multi-layered strategy was followed to maximize the generalizability of the developed machine learning models to different patient groups, clinical settings, and data conditions. The sample, consisting of 126 individuals aged between 18 and 60, exceeded the minimum sample size calculated via G*Power and provided high statistical power. The recording protocol was defined in detail (sampling frequency, microphone type, distance, type of vocal sample), and hardware information was shared, thus making the recording conditions reproducible across different centres.

Class balance (preop/postop = 1:1) was maintained, and model diversity was ensured by using different SVM kernels and Boosting algorithms. Feature selection was performed only on the training set, and the test set was completely isolated from this process to prevent data leakage and ensure realistic generalizability. The performance metrics of the models were reported with 95% confidence intervals, emphasizing the robustness of the results against sample variation.

SHAP analyses were conducted separately for each model over 100 iterations; only features showing consistent SHAP distribution in high-performance models (AUC ≥ 0.80) were considered biomarker candidates. Scatter plot analyses based on the literature presented model-independent patterns in the stability assessments. Full compliance with the TRIPOD-AI checklist further strengthened the potential of the study results in terms of transparency, clinical integration, and broad applicability.

#### 6.2.2. Limitations of Generalizability

Although various engineering and methodological precautions were taken in this study to enhance generalizability, certain limitations should be considered:

- The fact that data were collected solely from Gülhane Training and Research Hospital limits direct generalization to patient groups from different geographic regions or clinical practices.
- Only /a/ and /i/ sounds were used in the study, and spontaneous speech or sentence-based recordings were not included. This may restrict model performance in natural speech scenarios.
- The dataset, consisting of a total of 252 recordings, may have limited generalizability to voice data collected using different devices, microphones, or clinical settings.
- The participant group consisted of Turkish-speaking individuals with similar clinical profiles, which may reduce the applicability of the identified biomarkers to other linguistic or cultural populations.
- The use of self-report scales such as the VHI-10 and single-observer videolaryngostroboscopic evaluations introduces the limitation of the reference standard due to potential inter-individual interpretation differences.
- Some features, such as aSpectralCentroid and iSpectralCentroid, exhibited high variance between training and test sets, which was found to be limiting in terms of consistency and generalizability.
- Since the models were trained on the MATLAB platform and their integration with Electronic Health Record (EHR) systems has not yet been tested, there may be practical limitations in transitioning to clinical applicability.
- Although the model was proposed to be compatible with clinical decision support systems, this has not yet been validated in real-world environments and remains only a theoretical suggestion.

These limitations can be addressed in future studies through multi-centre designs, linguistic diversity, spontaneous speech data, and real-time integration testing.

### 6.3. Reproducibility

In this study, a high level of reproducibility was achieved through the structuring of acoustic analysis and machine learning processes based on scientific transparency and methodological consistency principles.

The voice recording protocol was described in detail, with technical variables such as the microphone type, sampling frequency, resolution, and microphone-to-mouth distance explicitly reported. This enables data generation under the same conditions.

The Butterworth filter (fourth order, 50 Hz cutoff) was applied using the filtfilt() function, and power spectral density calculations were performed using MATLAB functions pburg() and arfit(). These steps were shared with function names and parameters.

All models were trained using a fixed randomness seed (random_state = 2, generator = “twister”); the algorithms used (e.g., fitcsvm, fitcensemble), MATLAB R2025a version, CPU specifications, and operating system information were thoroughly documented.

All features (CPP, HNR, shimmer, etc.) and their computation sources (Praat, Troparion, Heller Murray script) were specified with version details. The shapley() function used for SHAP analysis was defined separately for kernel and tree SHAP algorithms.

The same modelling process was repeated over 100 iterations; in each iteration, z-score normalization, feature selection via LASSO, and SHAP calculation were performed. This strategy provided a reproducible analysis cycle by reducing the effect of random data splits.

SHAP values calculated with the training and test sets were compared using scatter plots, and features positioned close to the diagonal (e.g., iCPP, aCPP, aHNR) were identified as candidate biomarkers, indicating model-independent stability.

Model performance metrics were reported per iteration with the mean, the standard deviation, and 95% confidence intervals, thus presenting statistical uncertainties transparently.

Cohen’s d, the Mann–Whitney U test, and post hoc power analyses were defined with explicit formulas and performed using SPSS and G*Power software. This strengthened the reproducibility of clinical analyses.

All analysis scripts and sample code were made openly accessible on GitHub. Additionally, the TRIPOD-AI checklist is provided as a Supplementary File to document methodological reproducibility.

In this study, all methodological steps were described in a detailed and transparent manner to ensure reproducibility. The algorithms used, software versions, data processing steps, hyperparameter settings, and randomization strategies were systematically reported; analysis codes and scripts were made publicly available on GitHub. Furthermore, Python (version 3.11.7, Anaconda distribution) scripts included in the analysis flow and the originality of the feature matrix were shared in a verifiable manner via hash values generated with the SHA-256 algorithm in the ash_hash_report.json file. This approach ensures that the entire study can be replicated and validated step by step by independent researchers.

On the other hand, variations due to software incompatibility across platforms or randomization processes are methodologically possible. However, these risks have been largely minimized through the use of fixed random seeds, detailed algorithm documentation, and the open-source publication of all analysis steps.

### 6.4. Limitations

Although this study possesses numerous methodological strengths, it also has several limitations.

First, the voice recordings were evaluated using only the /a/ and /i/ vowels; natural speech, sentence-based, or dialogue-based samples were not analysed. This may limit the generalizability of the model to everyday speech dynamics. Additionally, all data were collected in a single centre (Gülhane Training and Research Hospital) using fixed equipment, and the system’s sensitivity to different equipment or environmental conditions in other centres has not been tested.

The fact that postoperative recordings were obtained only within the 4–6-week interval prevents the evaluation of late-stage PTVD effects. On the other hand, since the classification models were developed using CPU-based systems and SHAP analyses were performed over 100 iterations, a substantial computational load was generated; this may restrict the applicability of the approach in large datasets or real-time systems. Speed improvements could be achieved with GPU-supported systems.

The videolaryngostroboscopic evaluation was performed by a single observer (although blinded); inter-rater reliability analyses were not provided. Subjective scales like the VHI-10 may not always sensitively reflect the biophysical changes in voice.

Although the sample size (126 preop + 126 postop) is sufficient based on power analysis, it may still be considered relatively small from a machine learning perspective. This could limit model stability, particularly in features with high variance (e.g., aSpectralCentroid, iSpectralCentroid). Additionally, the post hoc power for the aHNR variable was calculated as 71.8%; thus, validation of this feature in larger samples is recommended.

Another limitation pertains to the potential influence of emotional state on vocal characteristics. Although the emotional background, particularly preoperative anxiety, may affect voice production, psychological evaluations such as the State–Trait Anxiety Inventory (STAI) were not administered in this study. This decision was partly due to the absence of a specific recommendation in clinical guidelines such as those of the American Thyroid Association (ATA) regarding the routine use of psychometric assessments in the pre- and postoperative evaluation of thyroidectomy patients. Nevertheless, the possible impact of anxiety on acoustic features is acknowledged. Future studies will take this limitation into account and consider incorporating standardized anxiety scales to better control for emotional confounding in voice analysis.

Although SHAP analyses were applied in all models and across all iterations, a generalized SHAP contribution pattern could not be obtained. This reflects the influence of model structures and data splitting on SHAP contributions, thereby producing model-dependent explainability.

While the model architectures are lightweight and technically compatible with clinical integration, they have not yet been tested in a real-time EHR system. Therefore, technical validation is still required during the clinical transition phase.

The clinical significance of features was examined not only based on SHAP contribution levels but also through their raw values. This multi-layered approach required the combined use of Cohen’s d, Mann–Whitney U, and post hoc power analyses. Although this adds complexity to the decision-making process, it enhances the methodological strength and clinical reliability.

Finally, since the study design is based on comparing preoperative and postoperative voices of the same patients, it is methodologically limited in terms of direct comparison with the literature based on healthy vs. dysphonic individuals. The subclinical and low-biological-separability nature of PTVD may reduce classification performance; the constancy of voice identity can make differentiation challenging for machine learning models.

## Figures and Tables

**Table 1:** Hyperparameter settings for all ML models.

Model	Hyperparameter	Value
Cubic SVM	Kernel Function	Polynomial
	Polynomial Degree	3
	Box Constraint (C)	0.1/1/0.01
	Kernel Scale	Auto
Quadratic SVM	Kernel Function	Polynomial
	Polynomial Degree	2
	Box Constraint (C)	0.1/1/0.01
	Kernel Scale	Auto
RBF SVM	Kernel Function	Gaussian
	Box Constraint (C)	0.1/1/0.01
	Kernel Scale	Auto
GentleBoost	Number of Learning Cycles	100/200/500
	Learning Rate	1/0.1/0.05/0.01
	Weak Learner Type	Decision Tree
	Max Number of Splits	10
	Min Parent Size	6
	Min Leaf Size	3
	Pruning	Off
	Merge Leaves	Off
LogitBoost	Number of Learning Cycles	100/200/500
	Learning Rate	1/0.1/0.05/0.01
	Weak Learner Type	Decision Tree
	Max Number of Splits	10
	Min Parent Size	6
	Min Leaf Size	3
	Pruning	Off
	Merge Leaves	Off

SVM: Support Vector Machine; RBF: Radial Basis Function.

**Table 2:** Demographic and clinical data of the cases.

	Female: 92	Male: 34	Total: 126	*p*-Value *
Mean Age	47.85 ± 11.80	50.21 ± 11.10	48.48 ± 11.66	0.238
Smoker (+)	20 (21.74%)	14 (41.18%)	34 (26.98%)	0.032
Pathological Diagnosis				
Malign	33 (35.87%)	13 (38.24%)	46 (36.51%)	0.713
Benign	59 (64.13%)	21 (61.76%)	80 (63.49%)	
Thyroid Surgery Type				
UTT	10 (10.87%)	4 (11.76%)	14 (11.11%)	0.710
BTT	82 (89.13%)	30 (88.24%)	112 (88.89%)	

Percentages are presented per column. * *p*-values were analysed in relation to the PTVD diagnosis. UTT: Unilateral Total Thyroidectomy; BTT: Bilateral Total Thyroidectomy.

**Table 3:** Distribution of preoperative and postoperative VHI-10 scores by gender.

Group	Preop VHI-10 (Mean ± SD)	Postop VHI-10 (Mean ± SD)	Preop VHI ≥ 11 *n* (%)	Postop VHI ≥ 11 *n* (%)	*p*-Value	PTVD *n* (%)
Female (*n* = 92)	10.37 ± 5.10	13.29 ± 5.00	24 (26.09%)	43 (46.74%)	0.000 *	19 (20.65%)
Male (*n* = 34)	9.44 ± 4.04	12.68 ± 5.05	9 (26.47%)	12 (35.29%)	0.000 *	3 (8.82%)
Total (*n* = 126)	10.12 ± 4.87	13.13 ± 5.04	33 (26.98%)	55 (39.68%)	0.000 *	22 (17.46%)

* *p* < 0.001. SD: standard deviation; VHI-10: Voice Handicap Index-10.

**Table 4:** Effect of prominent acoustic features in the diagnosis of PTVD.

Feature	Cohen’s d	Effect Size	AUC	Optimal Cutoff	Sensitivity	Specificity	Youden Index
iCPP	−2.95	Very Large	0.66	−10.10	0.39	0.98	0.37
aCPP	−1.13	Large	0.64	−16.45	0.89	0.34	0.22
aHNR	−0.60	Medium	0.59	−18.42	0.48	0.74	0.22

AUC: Area Under Curve; CPP: cepstral peak prominence; HNR: harmonic-to-noise ratio.

## Data Availability

The anonymized acoustic feature matrix used in this study (Supplementary File S29), along with all codes employed in the analysis, has been made openly available on the GitHub platform to ensure reproducibility: https://github.com/acousticStudy/Potential_Biomarkers_PTVD (created on 9 July 2025). The shared dataset has been anonymized to exclude any personally identifiable information in accordance with the Turkish Personal Data Protection Law No. 6698 (KVKK).

## Supplementary Materials

The following supporting information can be downloaded at https://www.mdpi.com/article/10.3390/diagnostics15162065/s1, Supplementary File S1—Acoustic Features (docx): List and descriptions of all extracted acoustic features used in the study. Supplementary File S2—TRIPOD-AI checklist (pdf): Reporting checklist ensuring compliance with TRIPOD-AI guidelines. Supplementary File S3—Model Performance Validation Results (docx): Detailed validation metrics for each classification model. Supplementary File S4—Model Performance Test Results (docx): Test set performance metrics for all models. Supplementary File S5—ROC SVM Models (png): ROC curves for Support Vector Machine models. Supplementary File S6—DeLong Full Results (xlsx): Complete statistical comparison results from DeLong’s test. Supplementary File S7—DeLong P Values Heatmap (png): Visualization of p-values from DeLong’s test across model comparisons. Supplementary File S8—DeLong AUC Differences Heatmap (png): Heatmap showing differences in AUC values from DeLong’s test. Supplementary File S9—AUC Statistical Test Results (xlsx): Full statistical output for AUC comparisons. Supplementary File S10—AUC P Values Heatmap (png): Heatmap visualization of p-values for AUC comparisons. Supplementary File S11—AUC Diff Heatmap (png): Heatmap of AUC differences between models. Supplementary File S12—ROC GentleBoost Models (png): ROC curves for GentleBoost models. Supplementary File S13—ROC LogitBoost Models (png): ROC curves for LogitBoost models. Supplementary File S14—Accuracy Statistical Test Results (xlsx): Statistical analysis results for accuracy comparisons. Supplementary File S15—Accuracy P Values Heatmap (png): Heatmap of p-values for accuracy comparisons. Supplementary File S16—Accuracy Diff Heatmap (png): Heatmap showing accuracy differences between models. Supplementary File S17—F1 Score Statistical Test Results (xlsx): Statistical test results for F1 score comparisons. Supplementary File S18—F1 Score P Values Heatmap (png): Heatmap of p-values for F1 score comparisons. Supplementary File S19—F1 Score Diff Heatmap (png): Heatmap showing F1 score differences between models. Supplementary File S20—Precision Statistical Test Results (xlsx): Statistical analysis of precision differences between models. Supplementary File S21—Precision P Values Heatmap (png): Heatmap of p-values for precision comparisons. Supplementary File S22—Precision Diff Heatmap (png): Heatmap showing precision differences between models. Supplementary File S23—Recall Statistical Test Results (xlsx): Statistical test results for recall comparisons. Supplementary File S24—Recall P Values Heatmap (png): Heatmap of p-values for recall comparisons. Supplementary File S25—Recall Diff Heatmap (png): Heatmap showing recall differences between models. Supplementary File S26—Specificity Statistical Test Results (xlsx): Statistical analysis of specificity comparisons. Supplementary File S27—Specificity P Values Heatmap (png): Heatmap of p-values for specificity comparisons. Supplementary File S28—Specificity Diff Heatmap (png): Heatmap showing specificity differences between models. Supplementary File S29—Dataset (xlsx): Anonymized acoustic feature dataset used in the study.

## Abbreviations

The following abbreviations are used in this manuscript:

AAO-HNS	American Academy of Otolaryngology—Head and Neck Surgery
aCPP	/a/ phonation cepstral peak prominence
aHNR	/a/ phonation harmonic-to-noise ratio
APQ3	Amplitude Perturbation Quotient (3 cycles)
APQ5	Amplitude Perturbation Quotient (5 cycles)
ATA	American Thyroid Association
AUC	Area Under the Curve
AVQI	Acoustic Voice Quality Index
BTA	British Thyroid Association
BTT	Bilateral Total Thyroidectomy
CI	Confidence interval
CPP	Cepstral peak prominence
CPU	Central processing unit
EBSLN	External branch of the superior laryngeal nerve
EHR	Electronic Health Record
f_0_	Fundamental frequency
F1, F2	Formant 1, formant 2
F1BW, F2BW	Bandwidth of formant 1, 2
FN	False negative
FP	False positive
GPU	Graphical processing unit
HNR	Harmonic-to-noise ratio
INMSG	International Neural Monitoring Study Group
iCPP	/i/ phonation cepstral peak prominence
KVKK	Kişisel Verilerin Korunması Kanunu (Turkish Personal Data Protection Law)
LASSO	Least Absolute Shrinkage and Selection Operator
L-EMG	Laryngeal electromyography
ML	Machine learning
PPQ5	Pitch Perturbation Quotient (5 cycles)
PSD	Power spectral density
PTVD	Post-thyroidectomy vocal dysfunction
RAP	Relative Average Perturbation
RBF	Radial Basis Function
RLN	Recurrent laryngeal nerve
ROC	Receiver operating characteristic
SD	Standard deviation
SHAP	SHapley Additive exPlanations
SMO	Sequential Minimal Optimization
SSD	Solid-State Drive
SVM	Support Vector Machine
TN	True negative
TP	True positive
TRIPOD-AI	Transparent Reporting of a Multivariable Prediction Model for Individual Prognosis or Diagnosis—Artificial Intelligence extension
UTT	Unilateral Total Thyroidectomy
VHI-10	Voice Handicap Index-10
VLS	Videolaryngostroboscopy
