# Supplementary material for: SHAP-Based Identification of Potential Acoustic Biomarkers in Patients with Post-Thyroidectomy Voice Disorder
Source: Diagnostics (Basel). 2025 Aug 18;15(16):2065. doi: 10.3390/diagnostics15162065 (PMC12385800; doi:10.3390/diagnostics15162065)
Supplement: Supplementary file 1 [file diagnostics-15-02065-s001.zip › Supplementary_File_1_Acoustic_Features.pdf]

Acoustic Features Extracted in the Study

Feature Name	Description	Software/Function Used	Reference
**Energy (Low, Mid, High)**	Total spectral energy within defined frequency bands	MATLAB – fft, abs, sum, log10	[Current study]
**Power (Low, Mid, High)**	Total spectral power within defined frequency bands	MATLAB – fft, duration-based division	[Current study]
**Area (Low, Mid, High)**	Total power spectral density within defined frequency bands	MATLAB (pburg)	[Current study]
**Max Peak (Low, Mid, High)**	Maximum spectral peak within each frequency band	MATLAB (pburg)	[Current study]
**Mean Peak (Low, Mid, High)**	Average peak value within each frequency band	MATLAB (pburg)	[Current study]
**Fundamental Frequency (f_0)**	Lowest frequency of the periodic component of the voice signal	MATLAB (pitch)	[35]
**Formants (F1, F2)**	First and second resonance frequencies of the vocal tract	Praat (To Formant - Burg)	[26, 36]
**F1/F2 Ratio**	Ratio between F1 and F2; reflects articulatory characteristics	Derived from formant values	[36]
**Formant Bandwidths (F1BW, F2BW)**	Bandwidth around F1 and F2; reflects sharpness of resonances	Praat	[26, 36]
**Harmonics-to-Noise Ratio (HNR)**	Ratio of harmonic to noise components	Praat (To Harmonicity)	[37,38]
**Jitter (Local, RAP, PPQ5)**	Cycle-to-cycle variation in fundamental frequency	Troparion (irapt1)	[39,40]
**Shimmer (Local, APQ3, APQ5)**	Cycle-to-cycle variation in amplitude	Troparion (irapt1)	[39,40]
**Spectral Centroid**	Center of mass of the frequency spectrum	MATLAB (spectralCentroid)	[41,42]
**Spectral Flux**	Rate of spectral change over time	MATLAB (spectralFlux)	[41,42]
**Spectral Flatness**	Measure of spectral smoothness	MATLAB (spectralFlatness)	[43]
**Spectral Skewness**	Asymmetry of the spectral distribution	MATLAB (spectralSkewness)	[41]
**Spectral Entropy**	Spectral irregularity based on Shannon entropy	MATLAB (spectralEntropy)	[44]
**Cepstral Peak Prominence (CPP)**	Prominence of the cepstral peak, related to voice quality	Praat (Heller-Murray script)	[26, 45]
