# Supplementary material for: SHAP-Based Identification of Potential Acoustic Biomarkers in Patients with Post-Thyroidectomy Voice Disorder
Source: Diagnostics (Basel). 2025 Aug 18;15(16):2065. doi: 10.3390/diagnostics15162065 (PMC12385800; doi:10.3390/diagnostics15162065)
Supplement: Supplementary file 1 [file diagnostics-15-02065-s001.zip › Supplementary_File_3_Model_Performance_Validation_Results.pdf]

Table Val:

Model	Metric	Mean ± SD	95% CI (Lower)	95% CI (Upper)
Cubic SVM C=1	Accuracy	52.83 ± 11.59	51.56	54.12
	Precision	54.94 ± 15.36	53.33	56.66
	Recall	51.06 ± 23.30	48.40	53.87
	Specificity	54.61 ± 24.01	51.89	57.45
	F1-score	49.27 ± 18.29	47.23	51.39
	AUC	55.66 ± 7.20	54.25	57.08
Cubic SVM C=0.1	Accuracy	52.93 ± 11.59	51.65	54.19
	Precision	55.09 ± 15.42	53.45	56.83
	Recall	51.60 ± 23.72	48.93	54.35
	Specificity	54.26 ± 24.10	51.54	57.25
	F1-score	49.46 ± 18.60	47.33	51.50
	AUC	55.67 ± 7.02	54.30	57.05
Cubic SVM C=0.01	Accuracy	53.29 ± 11.89	51.97	54.61
	Precision	55.21 ± 15.34	53.53	56.88
	Recall	50.93 ± 24.38	48.18	53.80
	Specificity	55.63 ± 24.15	53.01	58.41
	F1-score	49.17 ± 19.08	47.00	51.29
	AUC	55.74 ± 6.97	54.37	57.10
Quadratic SVM C=1	Accuracy	68.14 ± 6.86	67.41	68.91
	Precision	70.34 ± 8.26	69.46	71.30
	Recall	64.80 ± 12.52	63.37	66.14
	Specificity	71.48 ± 13.42	69.98	72.99
	F1-score	66.57 ± 8.83	65.64	67.53
	AUC	74.14 ± 6.55	72.89	75.37
Quadratic SVM C=0.1	Accuracy	68.38 ± 6.53	67.69	69.10
	Precision	70.94 ± 8.09	70.02	71.85
	Recall	63.84 ± 11.54	62.47	65.14
	Specificity	72.95 ± 11.35	71.70	74.28
	F1-score	66.48 ± 8.36	65.48	67.36
	AUC	75.29 ± 5.38	74.32	76.25
Quadratic SVM C=0.01	Accuracy	66.08 ± 6.16	65.39	66.78
	Precision	68.08 ± 7.22	67.26	68.90
	Recall	61.68 ± 12.11	60.27	63.09
	Specificity	70.50 ± 10.03	69.34	71.67
	F1-score	64.01 ± 8.37	63.08	64.96
	AUC	72.92 ± 5.37	71.92	73.89
RBF SVM C=1	Accuracy	56.62 ± 6.22	55.85	57.33
	Precision	56.74 ± 6.57	56.00	57.48

	Recall	57.58 ± 11.46	56.28	58.87
	Specificity	55.69 ± 11.51	54.32	57.07
	F1-score	56.61 ± 7.74	55.66	57.52
	AUC	58.61 ± 6.31	57.37	59.84
RBF SVM C=0.1	Accuracy	18.16 ± 25.94	15.37	21.20
	Precision	18.24 ± 26.29	15.49	21.25
	Recall	17.46 ± 27.73	14.50	20.79
	Specificity	18.85 ± 28.86	15.77	22.19
	F1-score	17.06 ± 25.39	14.35	20.07
	AUC	57.19 ± 5.80	56.05	58.33
RBF SVM C=0.01	Accuracy	18.16 ± 25.94	15.37	21.20
	Precision	18.24 ± 26.29	15.49	21.25
	Recall	17.46 ± 27.73	14.50	20.79
	Specificity	18.85 ± 28.86	15.77	22.19
	F1-score	17.06 ± 25.39	14.35	20.07
	AUC	57.19 ± 5.80	56.05	58.33
GentleBoost NLC=100 LR=0.1	Accuracy	72.48 ± 5.06	71.91	73.05
	Precision	73.30 ± 6.01	72.62	73.98
	Recall	71.60 ± 8.35	70.64	72.51
	Specificity	73.36 ± 8.36	72.39	74.25
	F1-score	72.11 ± 5.51	71.52	72.70
	AUC	79.74 ± 2.56	79.23	80.24
GentleBoost NLC=100 LR=1	Accuracy	72.48 ± 5.06	71.91	73.05
	Precision	73.30 ± 6.01	72.62	73.98
	Recall	71.60 ± 8.35	70.64	72.51
	Specificity	73.36 ± 8.36	72.39	74.25
	F1-score	72.11 ± 5.51	71.52	72.70
	AUC	79.74 ± 2.56	79.23	80.24
GentleBoost NLC=200 LR=0.1	Accuracy	72.83 ± 5.18	72.24	73.41
	Precision	73.68 ± 6.17	72.98	74.37
	Recall	71.96 ± 8.58	70.91	72.91
	Specificity	73.70 ± 8.52	72.75	74.64
	F1-score	72.45 ± 5.67	71.83	73.04
	AUC	80.10 ± 2.53	79.60	80.59
GentleBoost NLC=200 LR=0.05	Accuracy	72.83 ± 5.18	72.24	73.41
	Precision	73.68 ± 6.17	72.98	74.37
	Recall	71.96 ± 8.58	70.91	72.91
	Specificity	73.70 ± 8.52	72.75	74.64
	F1-score	72.45 ± 5.67	71.83	73.04
	AUC	80.10 ± 2.53	79.60	80.59
	Accuracy	72.83 ± 5.18	72.24	73.41

GentleBoost NLC=200 LR=1	Precision	73.68 ± 6.17	72.98	74.37
	Recall	71.96 ± 8.58	70.91	72.91
	Specificity	73.70 ± 8.52	72.75	74.64
	F1-score	72.45 ± 5.67	71.83	73.04
	AUC	80.10 ± 2.53	79.60	80.59
GentleBoost NLC=500 LR=0.01	Accuracy	73.14 ± 5.17	72.54	73.70
	Precision	73.99 ± 6.30	73.28	74.70
	Recall	72.32 ± 8.39	71.28	73.29
	Specificity	73.97 ± 8.57	72.99	74.97
	F1-score	72.79 ± 5.57	72.12	73.40
	AUC	80.34 ± 2.51	79.85	80.83
LogitBoost NLC=100 LR=0.1	Accuracy	70.64 ± 5.37	70.04	71.18
	Precision	71.57 ± 6.34	70.85	72.29
	Recall	69.45 ± 8.58	68.46	70.43
	Specificity	71.84 ± 8.67	70.91	72.81
	F1-score	70.15 ± 5.84	69.49	70.81
	AUC	77.87 ± 2.84	77.31	78.42
LogitBoost NLC=100 LR=1	Accuracy	71.83 ± 5.15	71.24	72.41
	Precision	72.74 ± 6.23	72.03	73.44
	Recall	70.79 ± 8.00	69.87	71.69
	Specificity	72.87 ± 8.58	71.93	73.86
	F1-score	71.43 ± 5.45	70.81	72.05
	AUC	79.23 ± 2.60	78.72	79.74
LogitBoost NLC=200 LR=0.1	Accuracy	70.97 ± 5.19	70.36	71.50
	Precision	71.87 ± 6.23	71.17	72.58
	Recall	69.81 ± 8.18	68.84	70.72
	Specificity	72.14 ± 8.39	71.19	73.10
	F1-score	70.51 ± 5.64	69.83	71.15
	AUC	78.39 ± 2.77	77.85	78.94
LogitBoost NLC=200 LR=0.05	Accuracy	70.59 ± 5.41	69.98	71.20
	Precision	71.51 ± 6.46	70.81	72.22
	Recall	69.48 ± 8.57	68.52	70.46
	Specificity	71.70 ± 8.90	70.72	72.68
	F1-score	70.13 ± 5.84	69.47	70.79
	AUC	77.93 ± 2.76	77.39	78.47
LogitBoost NLC=200 LR=1	Accuracy	71.01 ± 5.38	70.40	71.61
	Precision	67.68 ± 5.45	67.07	68.30
	Recall	81.72 ± 7.06	80.92	82.50
	Specificity	60.29 ± 9.99	59.16	61.42
	F1-score	73.81 ± 4.64	73.29	74.34
	AUC	79.75 ± 2.41	79.28	80.22

LogitBoost NLC=500 LR=0.01	Accuracy	69.74 ± 5.58	69.11	70.37
	Precision	70.97 ± 6.86	70.22	71.69
	Recall	67.96 ± 8.62	66.99	68.94
	Specificity	71.52 ± 9.22	70.51	72.53
	F1-score	69.06 ± 6.00	68.38	69.74
	AUC	76.77 ± 2.95	76.19	77.35

All values are reported as the mean ± standard deviation (SD), calculated over 100 independent iterations, along with the 95% confidence interval (CI). Metrics such as accuracy, precision, recall, specificity, F1-score, and AUC (area under the curve) are expressed as percentages. AUC values were derived from the receiver operating characteristic (ROC) analysis.
