# Supplementary material for: SHAP-Based Identification of Potential Acoustic Biomarkers in Patients with Post-Thyroidectomy Voice Disorder
Source: Diagnostics (Basel). 2025 Aug 18;15(16):2065. doi: 10.3390/diagnostics15162065 (PMC12385800; doi:10.3390/diagnostics15162065)
Supplement: Supplementary file 1 [file diagnostics-15-02065-s001.zip › Supplementary_File_4_Model_Performance_Test_Results.pdf]

Table T:

Model	Metric	Mean ± SD	95% CI (Lower)	95% CI (Upper)	Confusion Matrix
**Cubic SVM C=1**	Accuracy	70.84 ± 6.24	69.62	72.06	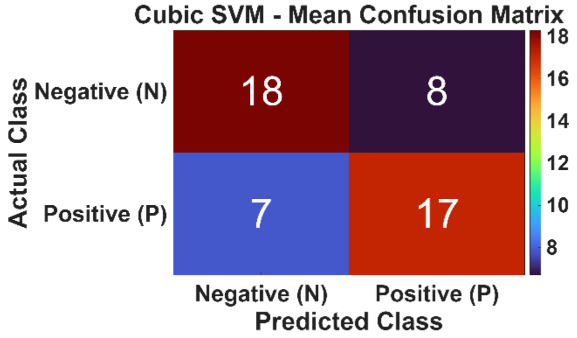
	Precision	72.31 ± 7.44	70.86	73.77	
	Recall	68.52 ± 8.82	66.79	70.25	
	Specificity	73.16 ± 9.35	71.28	74.88	
	F1-score	70.03 ± 6.65	68.73	71.34	
	AUC	75.94 ± 6.59	74.65	77.23	
**Cubic SVM C=0.1**	Accuracy	70.62 ± 5.83	69.48	71.70	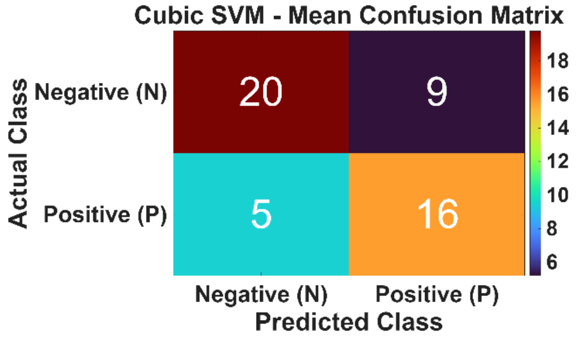
	Precision	75.30 ± 7.37	73.85	76.74	
	Recall	62.12 ± 10.29	60.10	64.14	
	Specificity	79.12 ± 8.03	77.46	80.74	
	F1-score	67.56 ± 7.54	66.08	68.95	
	AUC	78.07 ± 6.30	76.79	79.19	

**Cubic SVM C=0.01**	Accuracy	65.46 ± 5.86	64.31	66.61	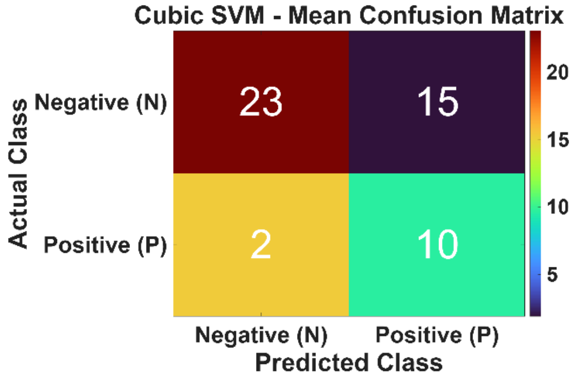
	Precision	83.87 ± 10.11	81.77	85.94	
	Recall	38.52 ± 11.00	36.36	40.68	
	Specificity	92.40 ± 5.67	91.20	93.52	
	F1-score	51.93 ± 11.22	49.73	54.13	
	AUC	77.88 ± 6.27	76.61	79.07	
**Quadratic SVM C=1**	Accuracy	69.76 ± 6.45	68.45	71.05	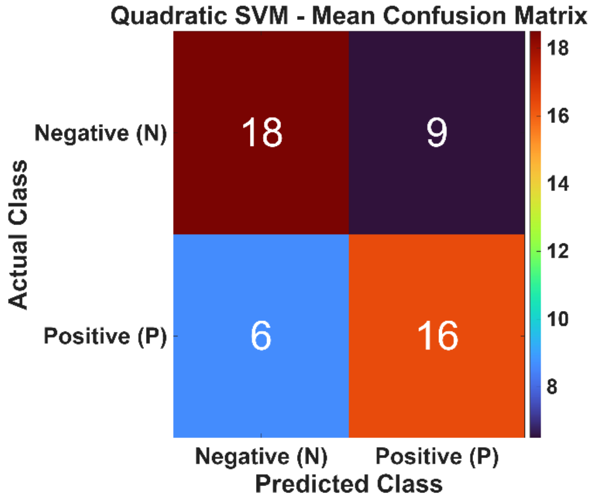
	Precision	72.19 ± 7.68	70.69	73.70	
	Recall	65.52 ± 9.89	63.64	67.36	
	Specificity	74.00 ± 9.79	72.12	75.84	
	F1-score	68.22 ± 7.02	66.87	69.61	
	AUC	76.07 ± 6.33	74.82	77.31	

**Quadratic SVM C=0.1**	Accuracy	68.98 ± 5.82	67.80	70.13	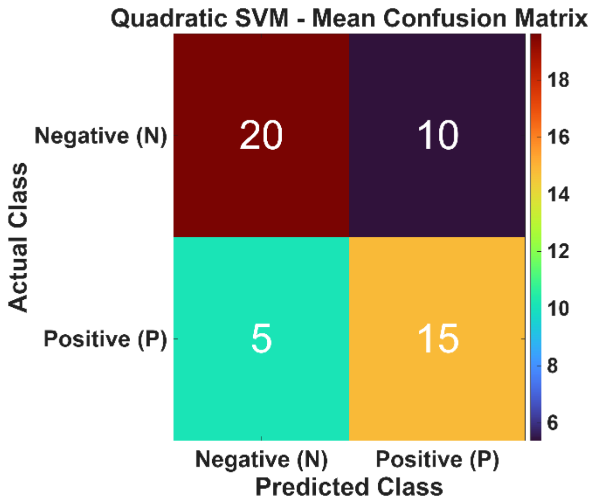
	Precision	74.10 ± 8.20	72.49	75.71	
	Recall	59.48 ± 9.73	57.57	61.39	
	Specificity	78.48 ± 8.84	76.70	80.04	
	F1-score	65.45 ± 7.34	63.99	66.90	
	AUC	77.50 ± 6.40	76.25	78.76	
**Quadratic SVM C=0.01**	Accuracy	62.96 ± 5.62	61.86	64.06	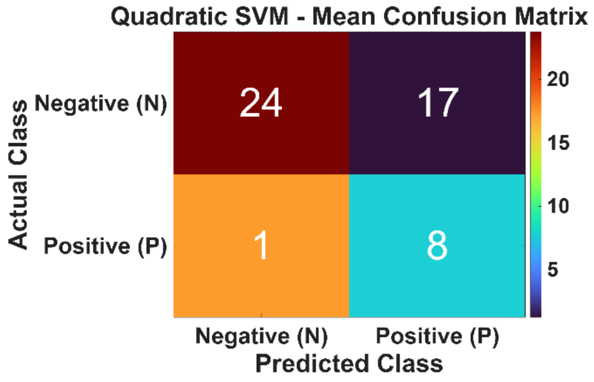
	Precision	86.00 ± 12.71	83.44	88.49	
	Recall	30.88 ± 10.65	28.90	33.08	
	Specificity	95.04 ± 4.41	94.16	95.90	
	F1-score	44.53 ± 12.47	42.01	46.97	
	AUC	76.55 ± 6.45	75.29	77.82	

**RBF SVM C=1**	Accuracy	70.34 ± 6.18	69.13	71.55	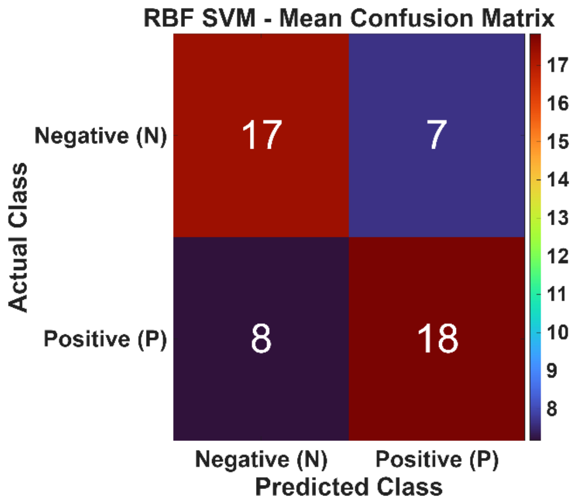
	Precision	70.50 ± 7.13	69.10	71.89	
	Recall	71.28 ± 8.85	69.58	72.98	
	Specificity	69.40 ± 10.22	67.40	71.40	
	F1-score	70.53 ± 6.15	69.32	71.73	
	AUC	77.31 ± 5.76	76.18	78.44	
**RBF SVM C=0.1**	Accuracy	66.56 ± 6.31	65.37	67.80	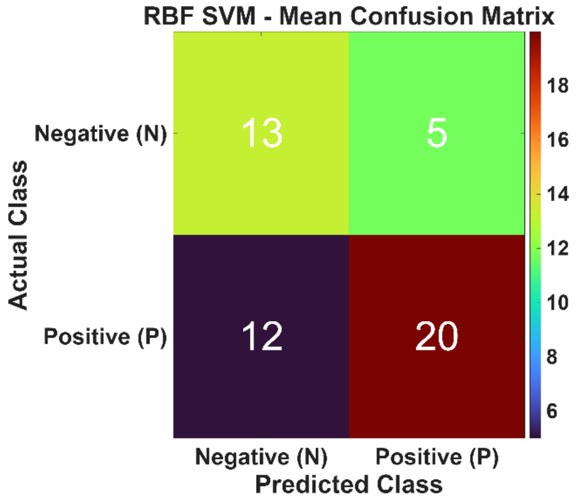
	Precision	63.45 ± 5.97	62.28	64.62	
	Recall	79.92 ± 8.26	78.44	81.56	
	Specificity	53.20 ± 11.19	50.96	55.36	
	F1-score	70.48 ± 5.39	69.53	71.55	
	AUC	75.27 ± 6.59	74.12	76.57	

**RBF SVM C=0.01**	Accuracy	66.70 ± 6.40	65.47	67.93
	Precision	63.72 ± 6.08	62.53	64.91
	Recall	79.36 ± 8.65	77.76	81.06
	Specificity	54.04 ± 11.26	51.84	56.20
	F1-score	70.40 ± 5.61	69.39	71.49
	AUC	75.21 ± 6.61	74.06	76.50
**GentleBoost NLC=100 LR=0.1**	Accuracy	72.44 ± 7.11	70.99	73.84
	Precision	73.19 ± 7.63	71.72	74.54
	Recall	71.60 ± 10.69	69.48	73.82
	Specificity	73.28 ± 9.58	71.22	75.02
	F1-score	71.99 ± 7.77	70.45	73.60
	AUC	80.37 ± 6.57	79.05	81.64

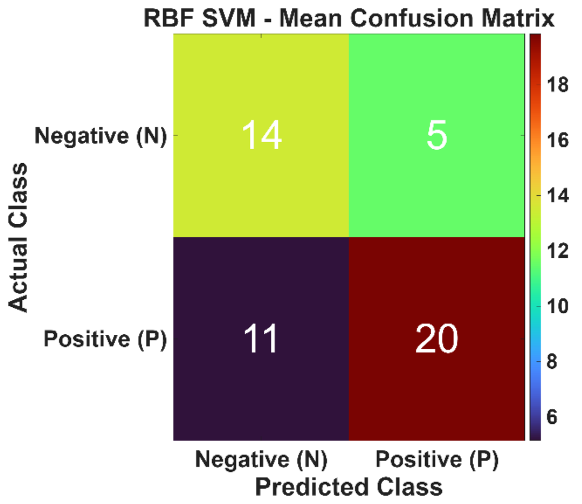

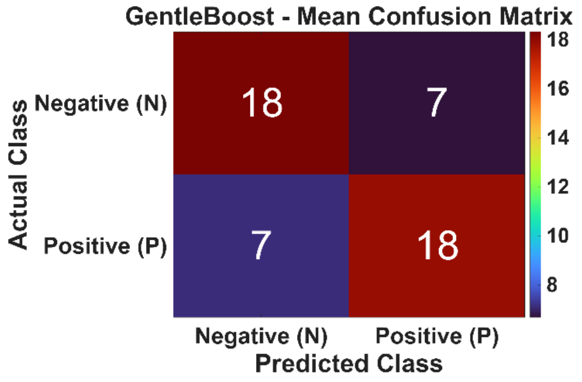

**GentleBoost NLC=100 LR=1**	Accuracy	72.44 ± 7.11	70.99	73.84	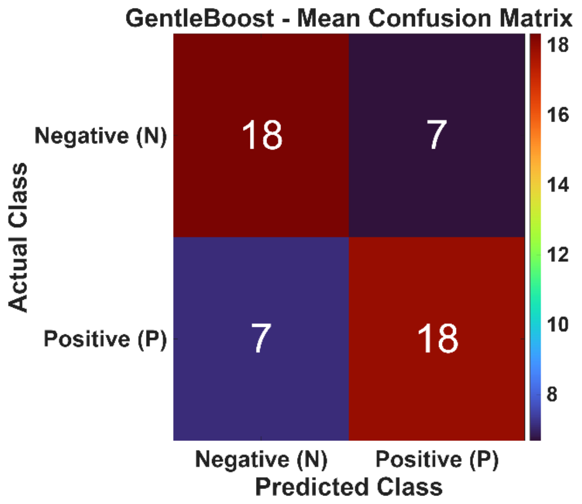
	Precision	73.19 ± 7.63	71.72	74.54	
	Recall	71.60 ± 10.69	69.48	73.82	
	Specificity	73.28 ± 9.58	71.22	75.02	
	F1-score	71.99 ± 7.77	70.45	73.60	
	AUC	80.37 ± 6.57	79.05	81.64	
**GentleBoost NLC=200 LR=0.1**	Accuracy	72.92 ± 6.93	71.51	74.24	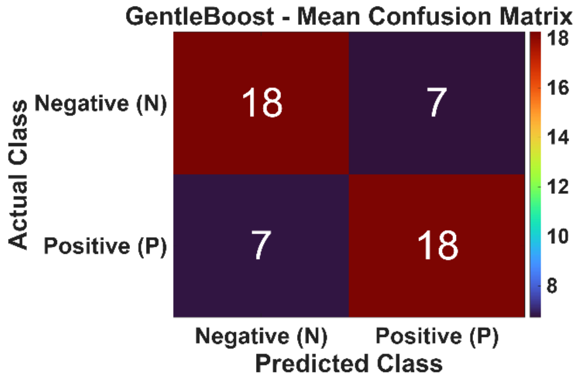
	Precision	73.36 ± 7.41	71.90	74.72	
	Recall	72.76 ± 10.09	70.82	74.90	
	Specificity	73.08 ± 9.41	71.26	74.82	
	F1-score	72.71 ± 7.31	71.22	74.19	
	AUC	80.87 ± 6.31	79.59	82.06	

<table>
<tr><td rowspan="6">GentleBoost NLC=200 LR=0.05</td><td>Accuracy</td><td>72.92 ± 6.93</td><td>71.51</td><td>74.24</td><td rowspan="6">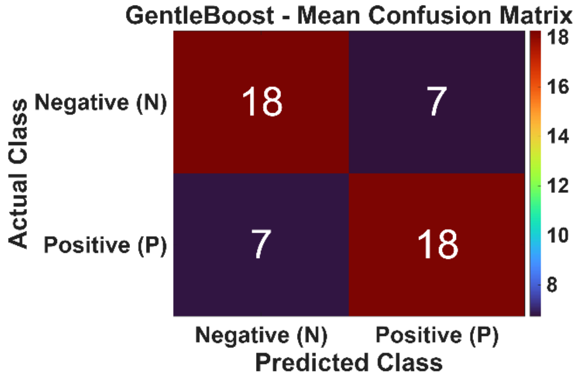
</td></tr>
<tr><td>Precision</td><td>73.36 ± 7.41</td><td>71.90</td><td>74.72</td></tr>
<tr><td>Recall</td><td>72.76 ± 10.09</td><td>70.82</td><td>74.90</td></tr>
<tr><td>Specificity</td><td>73.08 ± 9.41</td><td>71.26</td><td>74.82</td></tr>
<tr><td>F1-score</td><td>72.71 ± 7.31</td><td>71.22</td><td>74.19</td></tr>
<tr><td>AUC</td><td>80.87 ± 6.31</td><td>79.59</td><td>82.06</td></tr>
<tr><td rowspan="6">GentleBoost NLC=200 LR=1</td><td>Accuracy</td><td>72.92 ± 6.93</td><td>71.51</td><td>74.24</td><td rowspan="6">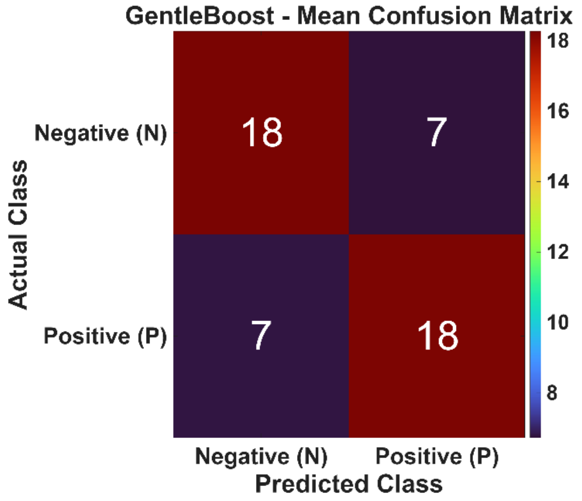
</td></tr>
<tr><td>Precision</td><td>73.36 ± 7.41</td><td>71.90</td><td>74.72</td></tr>
<tr><td>Recall</td><td>72.76 ± 10.09</td><td>70.82</td><td>74.90</td></tr>
<tr><td>Specificity</td><td>73.08 ± 9.41</td><td>71.26</td><td>74.82</td></tr>
<tr><td>F1-score</td><td>72.71 ± 7.31</td><td>71.22</td><td>74.19</td></tr>
<tr><td>AUC</td><td>80.87 ± 6.31</td><td>79.59</td><td>82.06</td></tr>
</table>

**GentleBoost NLC=500 LR=0.01**	Accuracy	73.24 ± 6.43	71.96	74.48	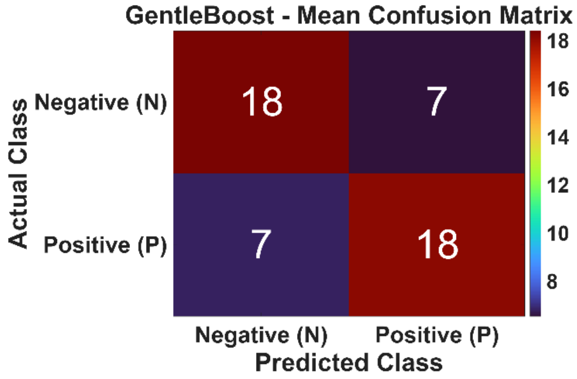
	Precision	73.90 ± 7.27	72.47	75.32	
	Recall	72.76 ± 9.53	70.86	74.72	
	Specificity	73.72 ± 9.38	71.88	75.32	
	F1-score	72.97 ± 6.79	71.62	74.33	
	AUC	81.23 ± 6.24	79.98	82.40	
**LogitBoost NLC=100 LR=0.1**	Accuracy	71.10 ± 6.78	69.73	72.38	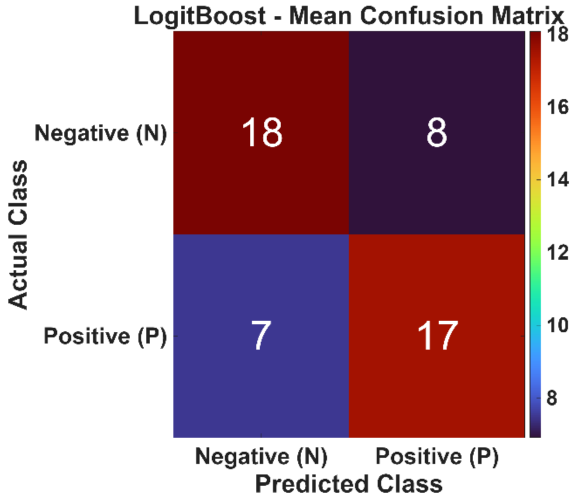
	Precision	72.24 ± 8.02	70.67	73.81	
	Recall	69.88 ± 9.84	67.92	71.82	
	Specificity	72.32 ± 10.63	70.12	74.28	
	F1-score	70.60 ± 7.12	69.20	71.88	
	AUC	79.18 ± 6.87	77.69	80.37	

**LogitBoost NLC=100 LR=1**	Accuracy	72.76 ± 7.21	71.35	74.17	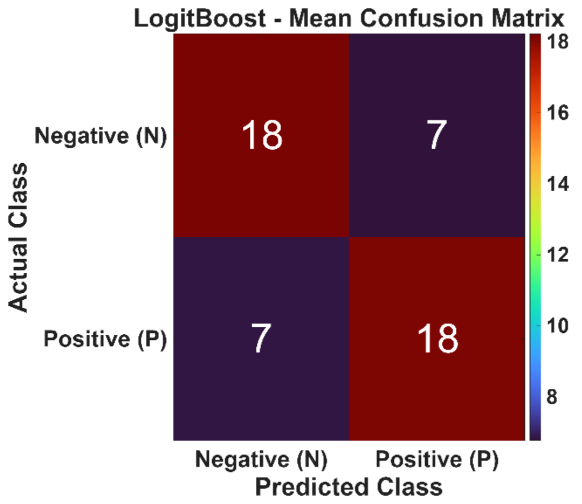
	Precision	73.29 ± 8.12	71.69	74.88	
	Recall	72.64 ± 10.20	70.62	74.76	
	Specificity	72.88 ± 10.20	70.80	74.74	
	F1-score	72.57 ± 7.64	71.08	74.07	
	AUC	80.07 ± 6.44	78.82	81.28	
**LogitBoost NLC=200 LR=0.1**	Accuracy	72.22 ± 6.96	70.86	73.58	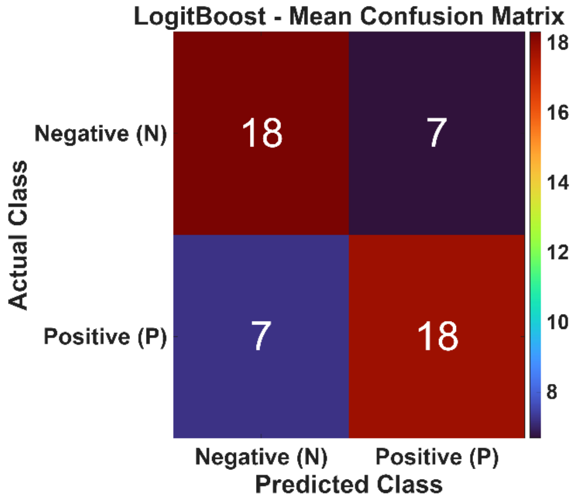
	Precision	73.15 ± 7.59	71.66	74.64	
	Recall	71.20 ± 10.09	69.16	73.24	
	Specificity	73.24 ± 10.04	71.16	75.00	
	F1-score	71.77 ± 7.31	70.34	73.20	
	AUC	79.65 ± 7.06	78.21	80.89	

**LogitBoost NLC=200 LR=0.05**	Accuracy	70.54 ± 6.90	69.12	71.83	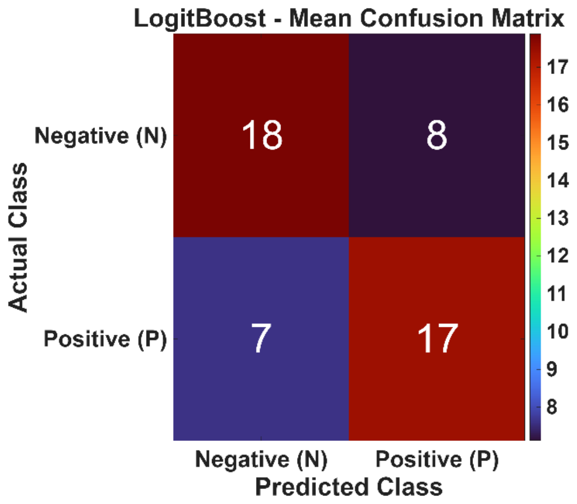
	Precision	71.47 ± 7.91	69.92	73.02	
	Recall	69.56 ± 10.01	67.60	71.52	
	Specificity	71.52 ± 10.44	69.28	73.46	
	F1-score	70.09 ± 7.33	68.60	71.44	
	AUC	79.00 ± 6.91	77.54	80.23	
**LogitBoost NLC=200 LR=1**	Accuracy	72.76 ± 6.23	71.54	73.98	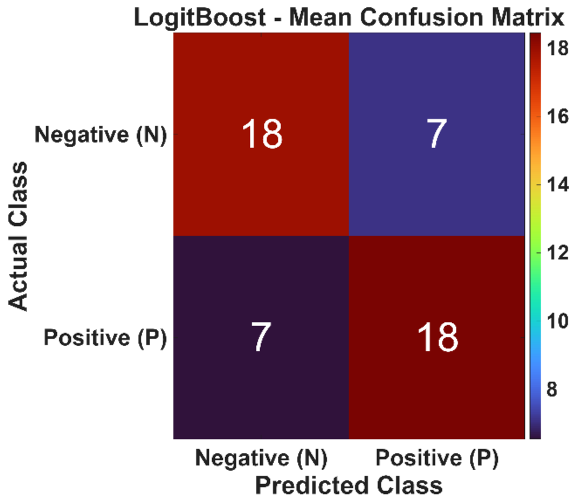
	Precision	72.77 ± 6.91	71.42	74.13	
	Recall	73.80 ± 9.57	71.92	75.74	
	Specificity	71.72 ± 9.82	69.60	73.44	
	F1-score	72.90 ± 6.49	71.63	74.17	
	AUC	80.40 ± 6.50	79.10	81.57	

**LogitBoost NLC=500 LR=0.01**	Accuracy	69.86 ± 6.60	68.57	71.15
	Precision	71.24 ± 7.84	69.70	72.77
	Recall	67.84 ± 9.43	65.98	69.72
	Specificity	71.88 ± 10.27	69.78	73.80
	F1-score	69.11 ± 7.01	67.73	70.48
	AUC	77.58 ± 6.95	76.17	78.84

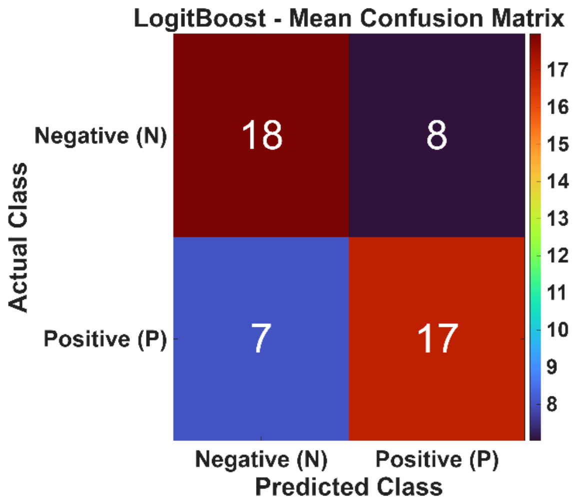

All values are reported as the mean ± standard deviation (SD), calculated over 100 independent iterations, along with the 95% confidence interval (CI). Metrics such as accuracy, precision, recall, specificity, F1-score, and AUC (area under the curve) are expressed as percentages. AUC values were derived from the receiver operating characteristic (ROC) analysis.
