# Supplementary figures and images for: SHAP-Based Identification of Potential Acoustic Biomarkers in Patients with Post-Thyroidectomy Voice Disorder
Source: Diagnostics (Basel). 2025 Aug 18;15(16):2065. doi: 10.3390/diagnostics15162065 (PMC12385800; doi:10.3390/diagnostics15162065)

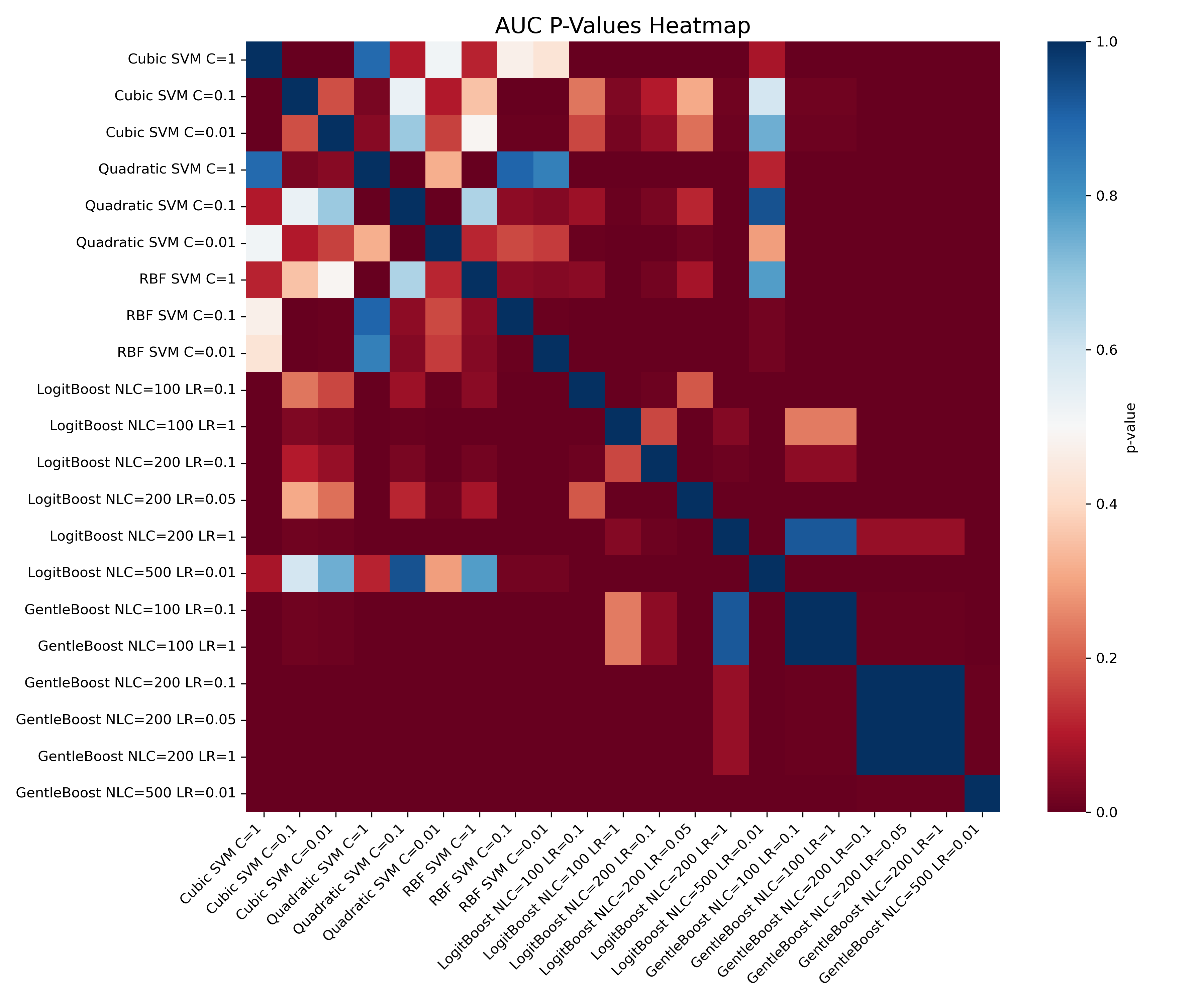

Supplement: Supplementary file 1 [file diagnostics-15-02065-s001.zip › Supplementary_File_10_AUC_P_Values_Heatmap.png]

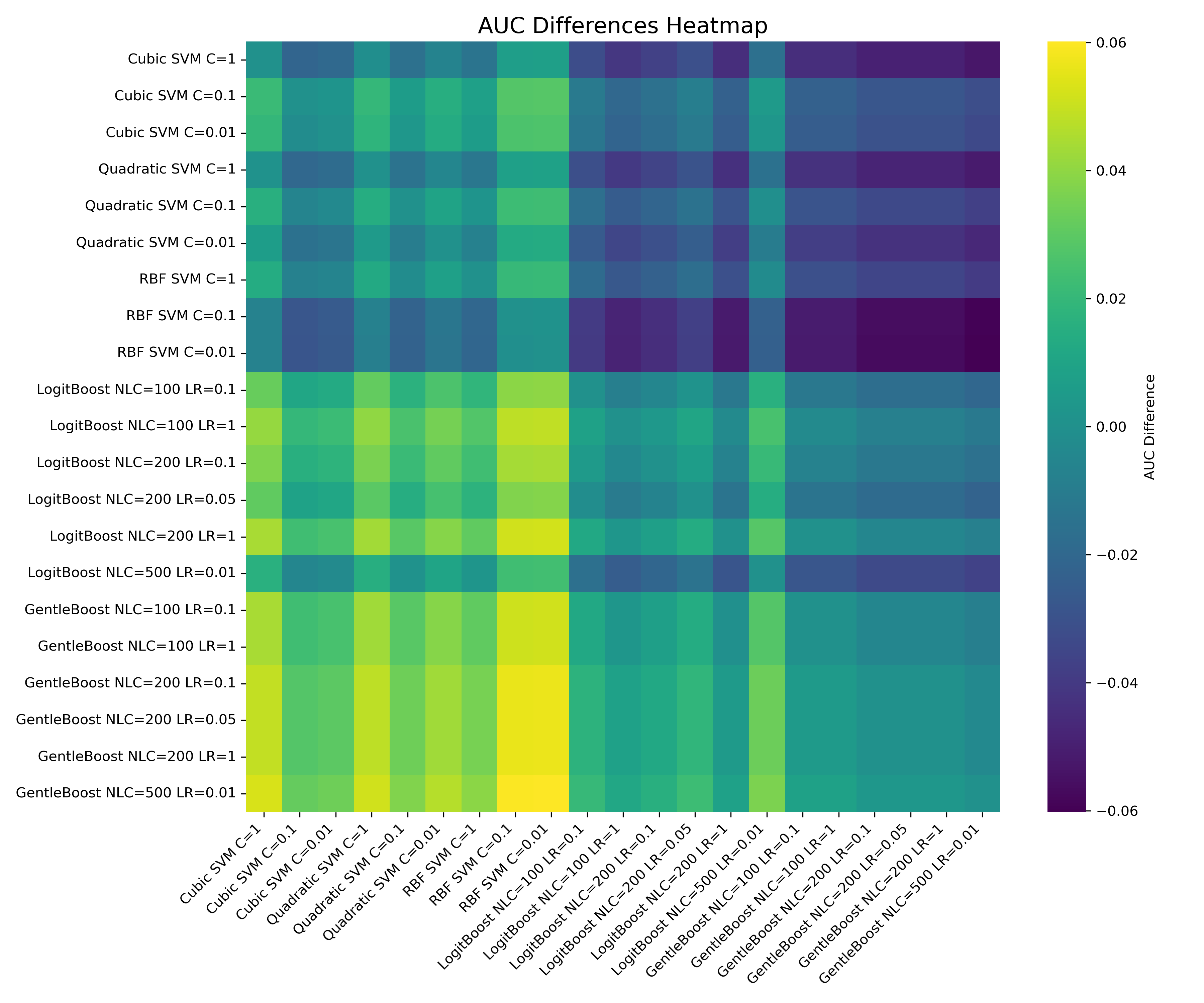

Supplement: Supplementary file 1 [file diagnostics-15-02065-s001.zip › Supplementary_File_11_AUC_Diff_Heatmap.png]

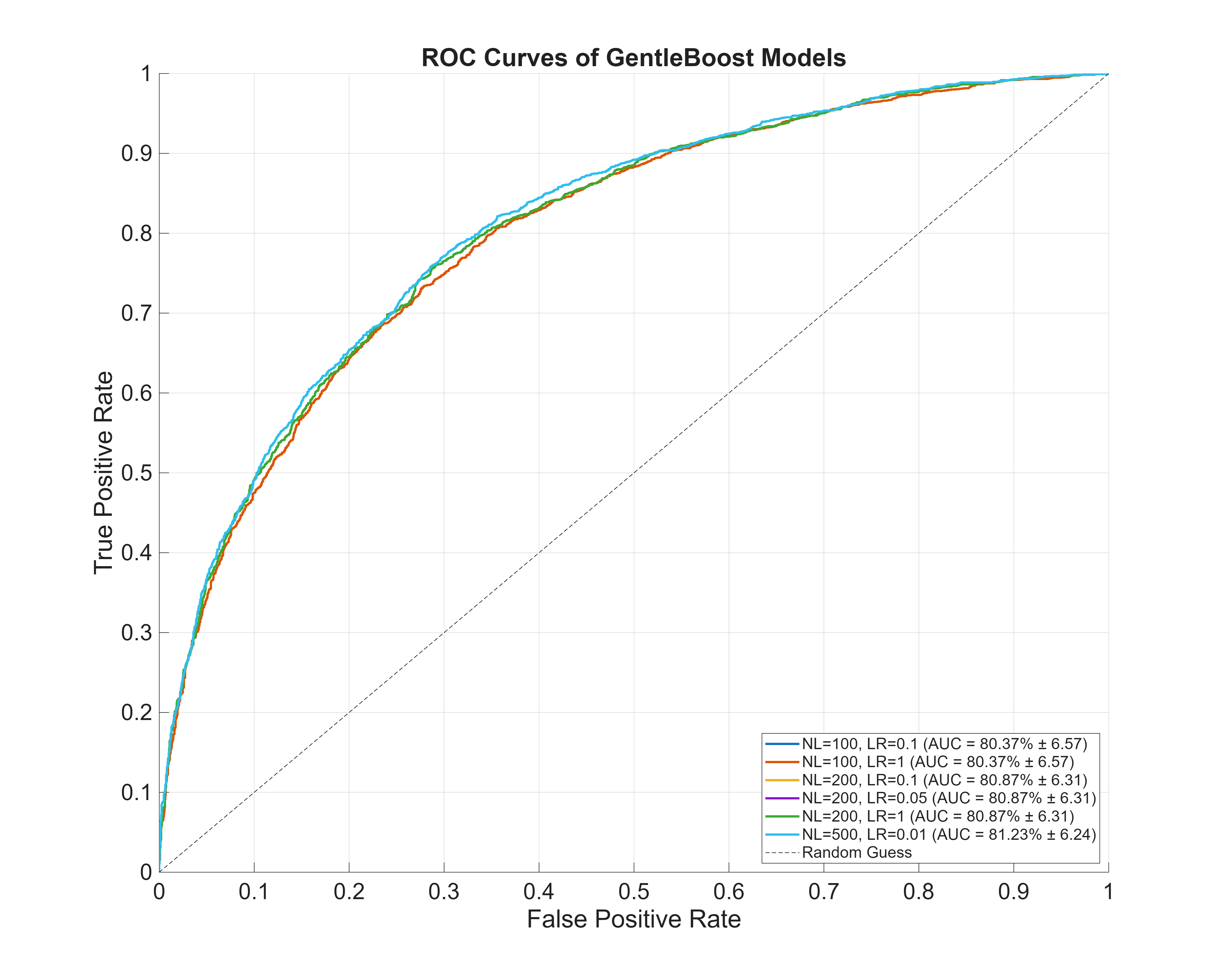

Supplement: Supplementary file 1 [file diagnostics-15-02065-s001.zip › Supplementary_File_12_ROC_GentleBoost_Models.png]

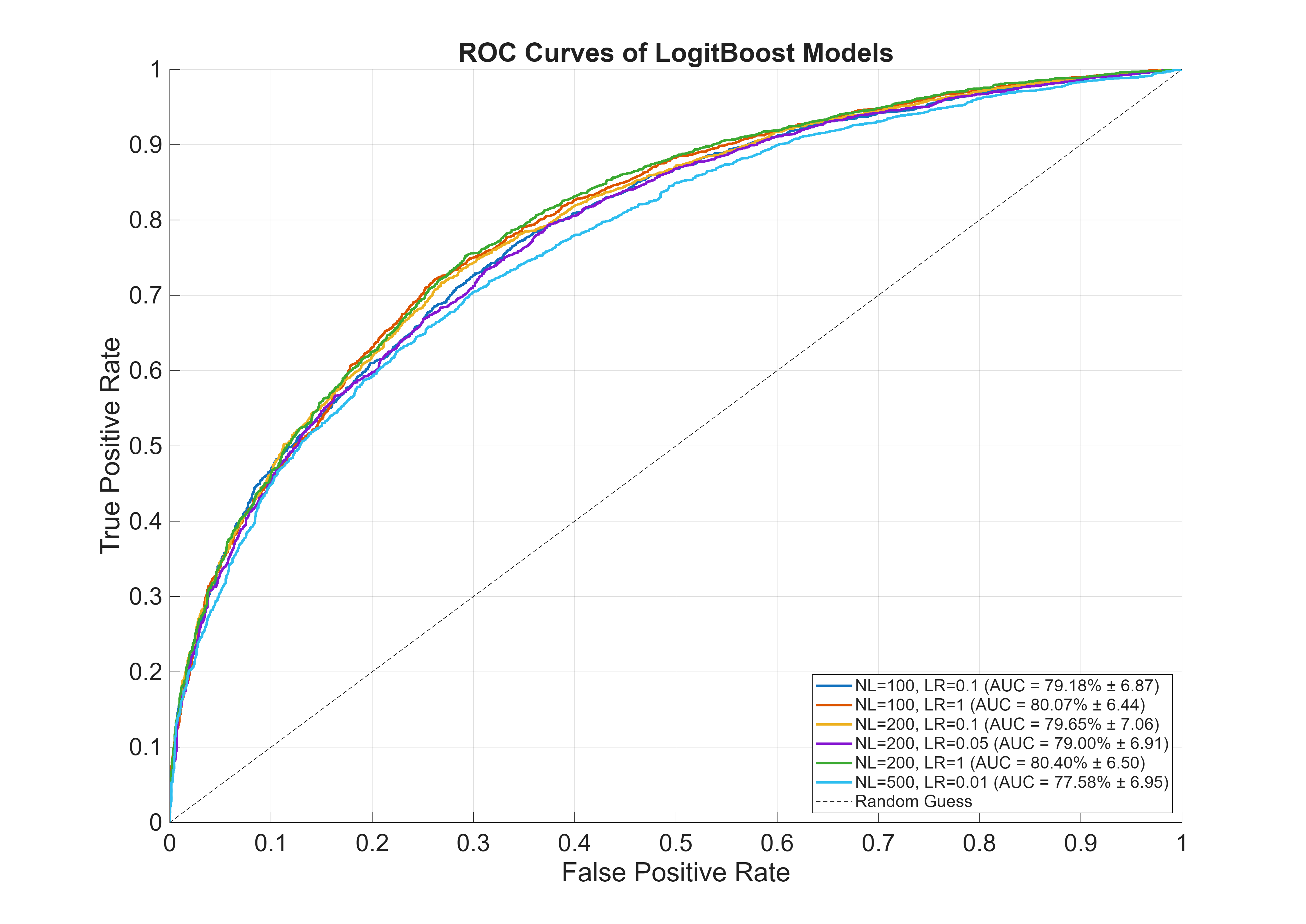

Supplement: Supplementary file 1 [file diagnostics-15-02065-s001.zip › Supplementary_File_13_ROC_LogitBoost_Models.png]

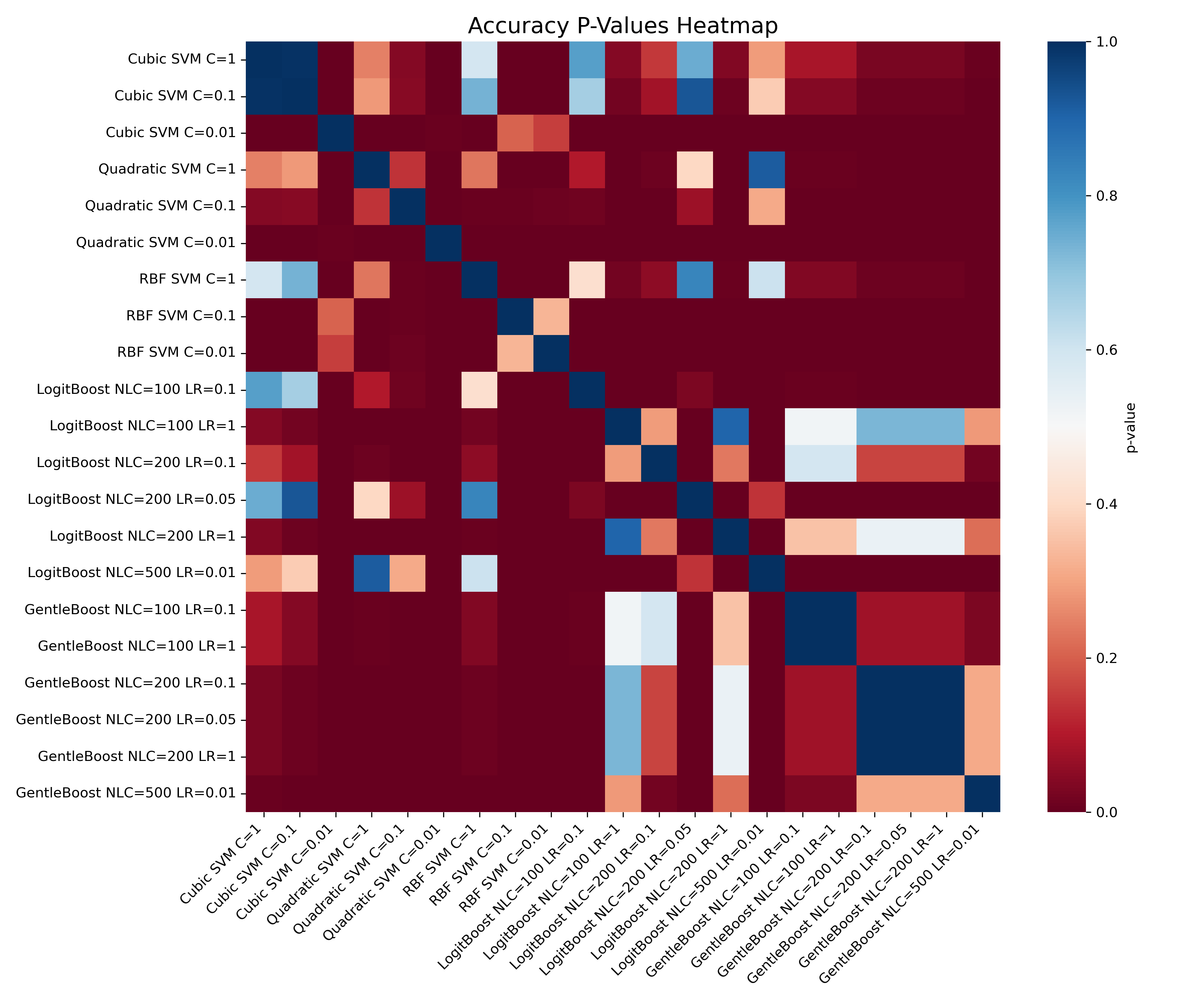

Supplement: Supplementary file 1 [file diagnostics-15-02065-s001.zip › Supplementary_File_15_Accuracy_P_Values_Heatmap.png]

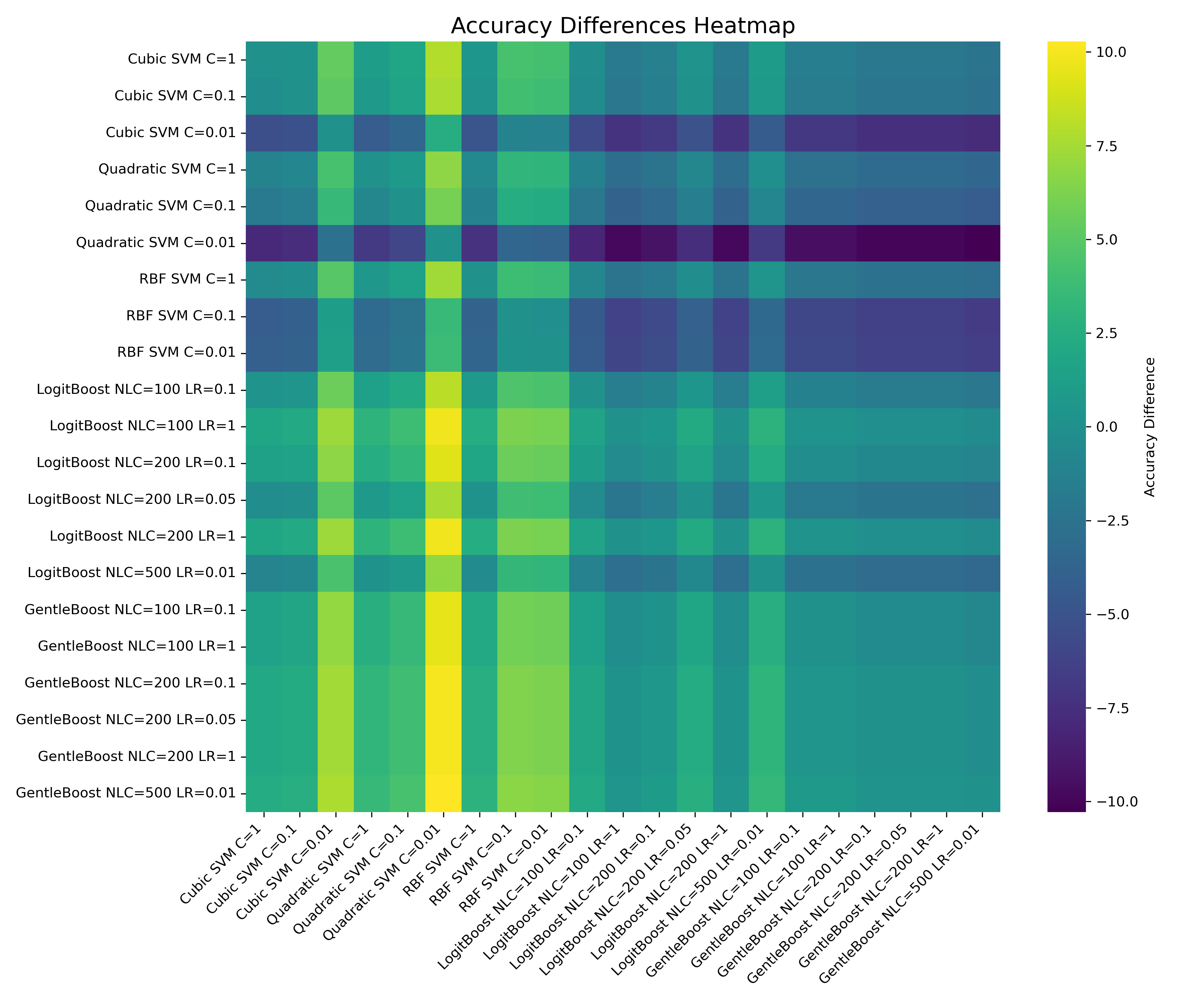

Supplement: Supplementary file 1 [file diagnostics-15-02065-s001.zip › Supplementary_File_16_Accuracy_Diff_Heatmap.png]

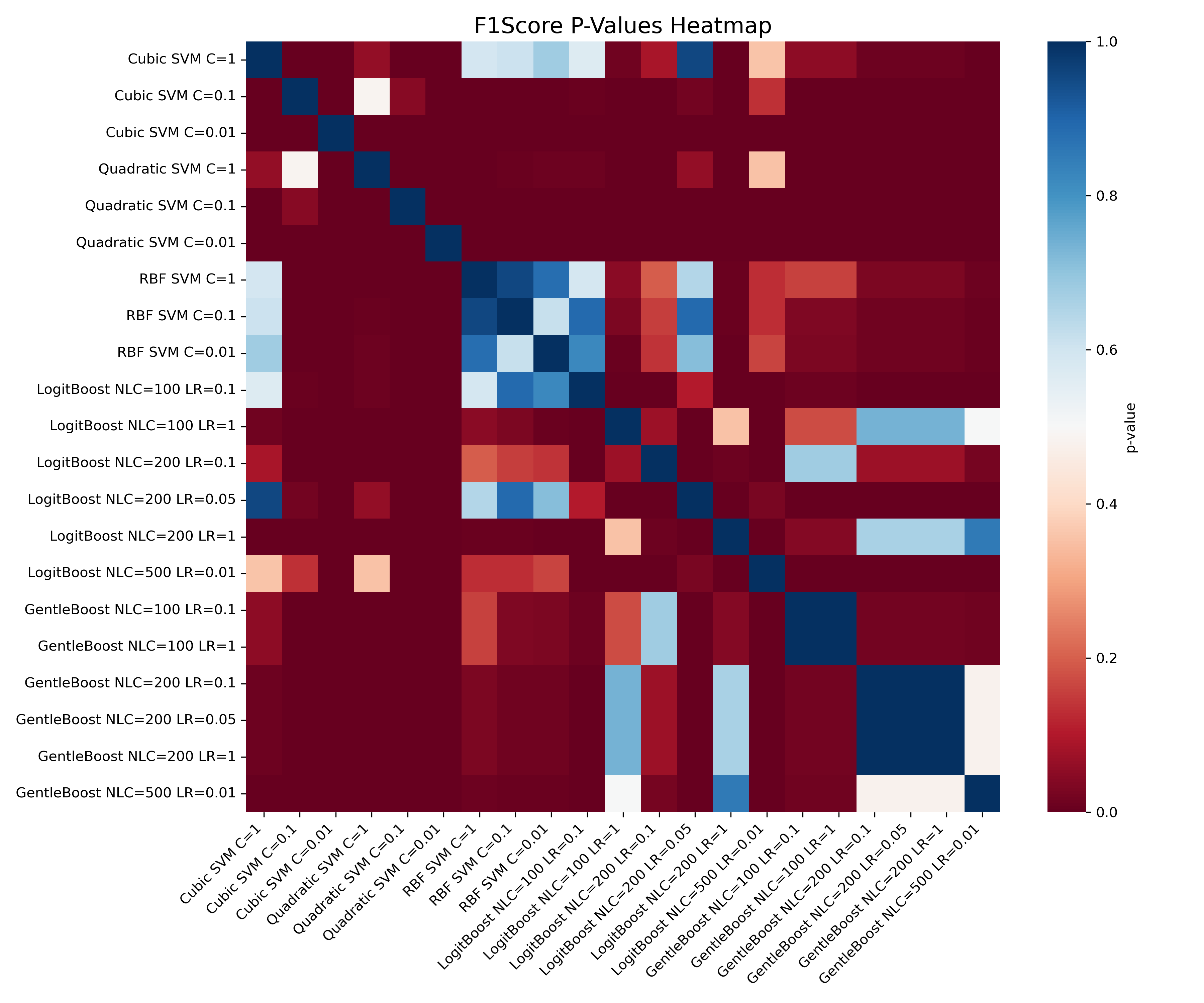

Supplement: Supplementary file 1 [file diagnostics-15-02065-s001.zip › Supplementary_File_18_F1Score_P_Values_Heatmap.png]

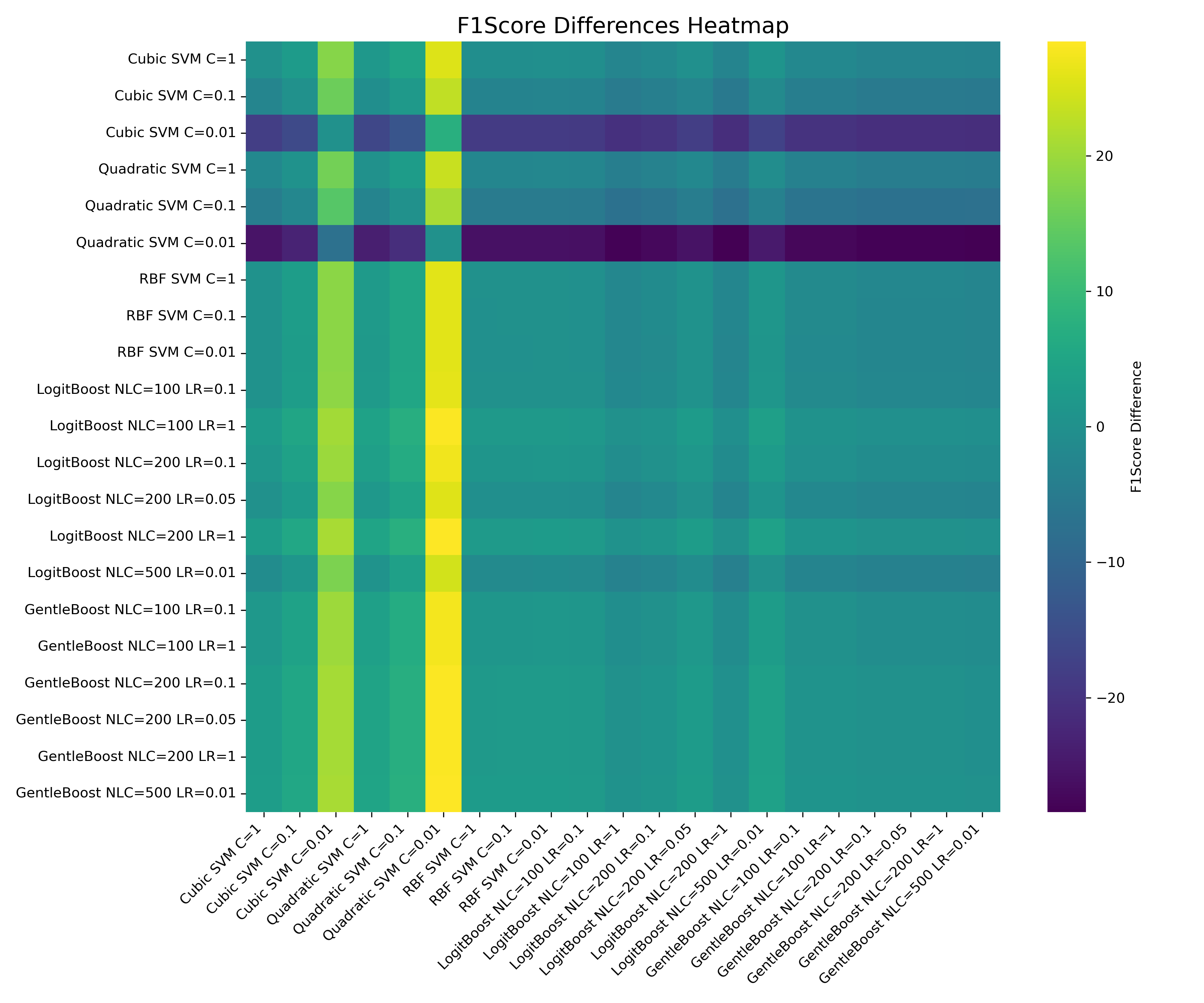

Supplement: Supplementary file 1 [file diagnostics-15-02065-s001.zip › Supplementary_File_19_F1Score_Diff_Heatmap.png]

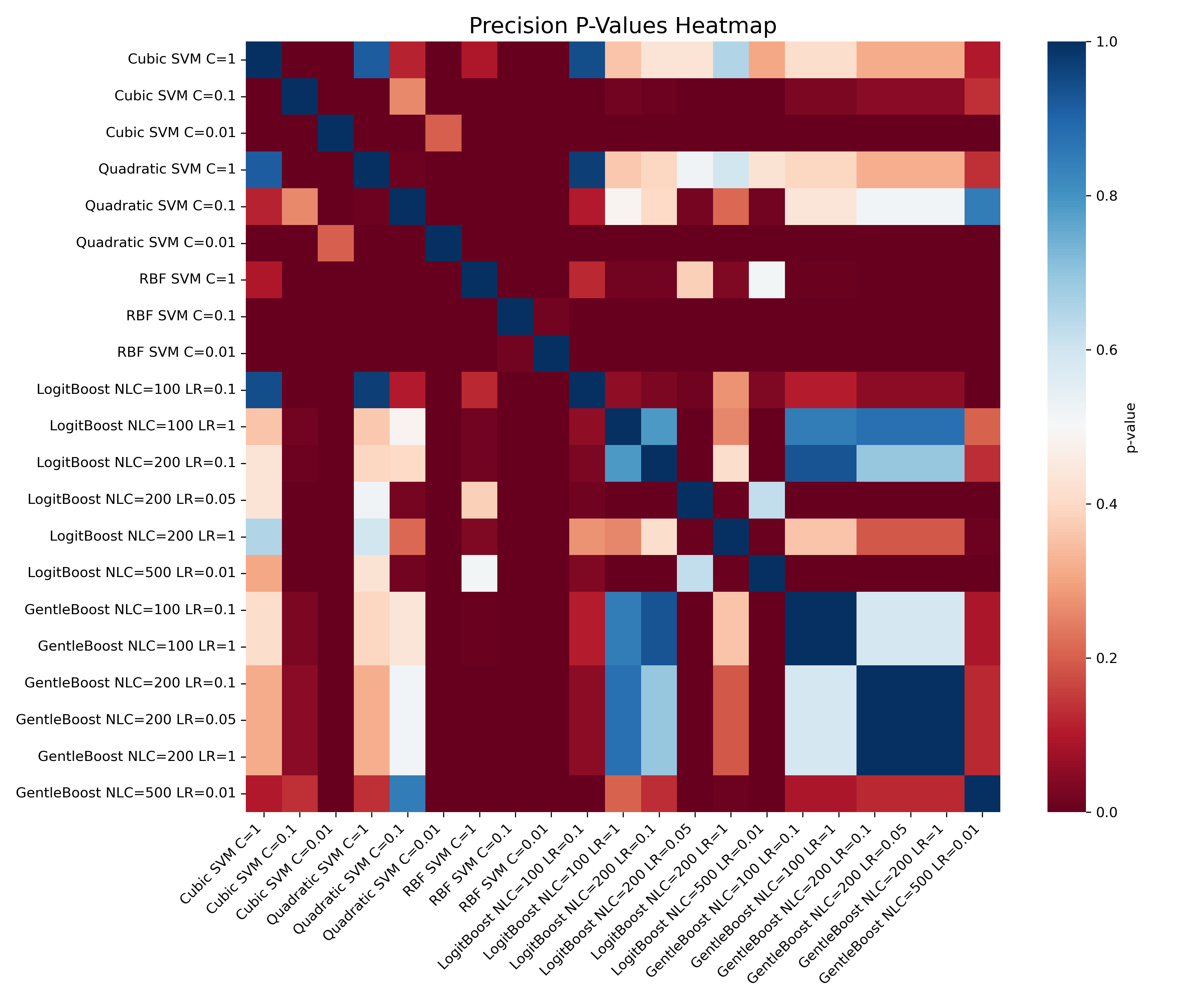

Supplement: Supplementary file 1 [file diagnostics-15-02065-s001.zip › Supplementary_File_21_Precision_P_Values_Heatmap.png]

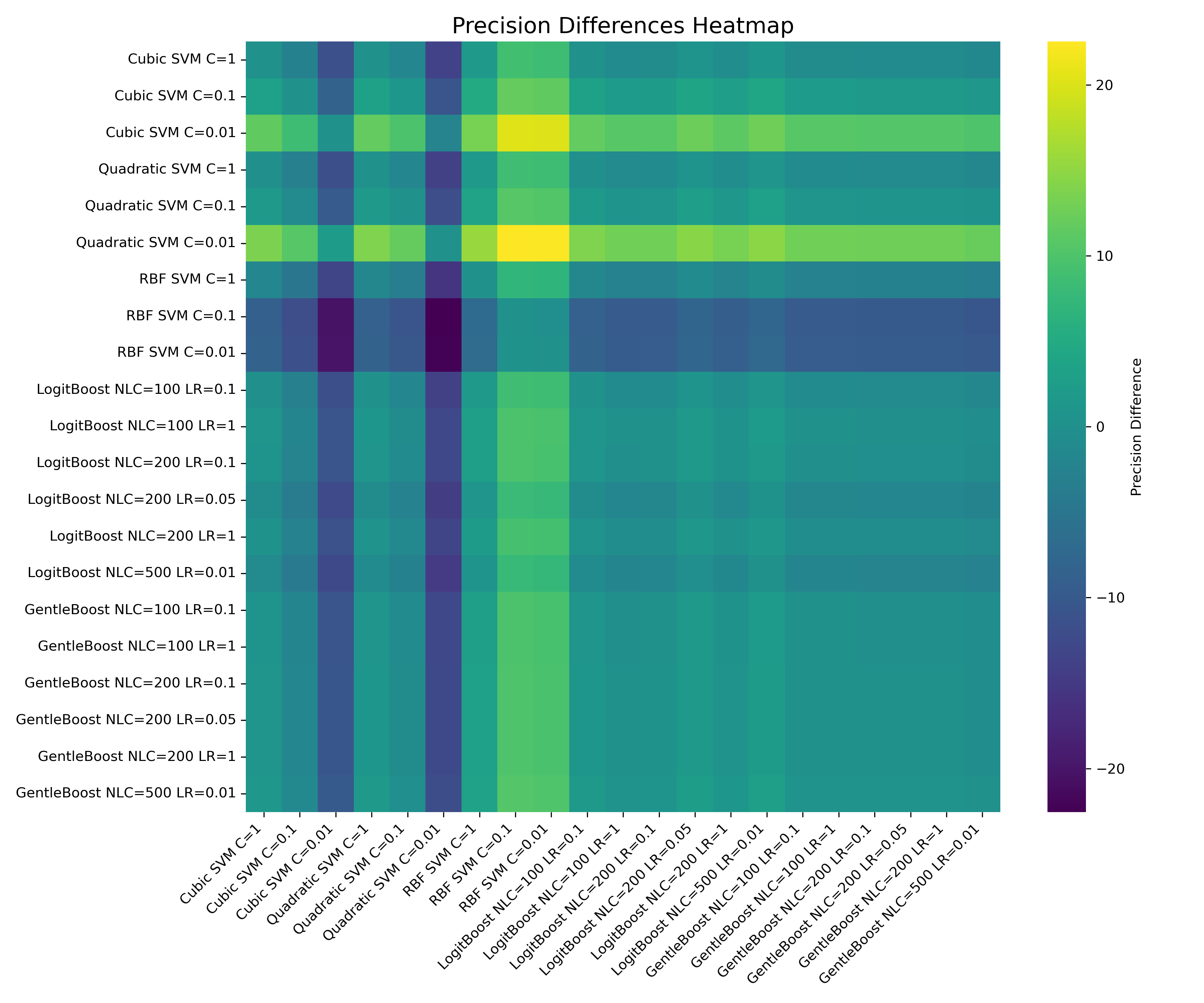

Supplement: Supplementary file 1 [file diagnostics-15-02065-s001.zip › Supplementary_File_22_Precision_Diff_Heatmap.png]

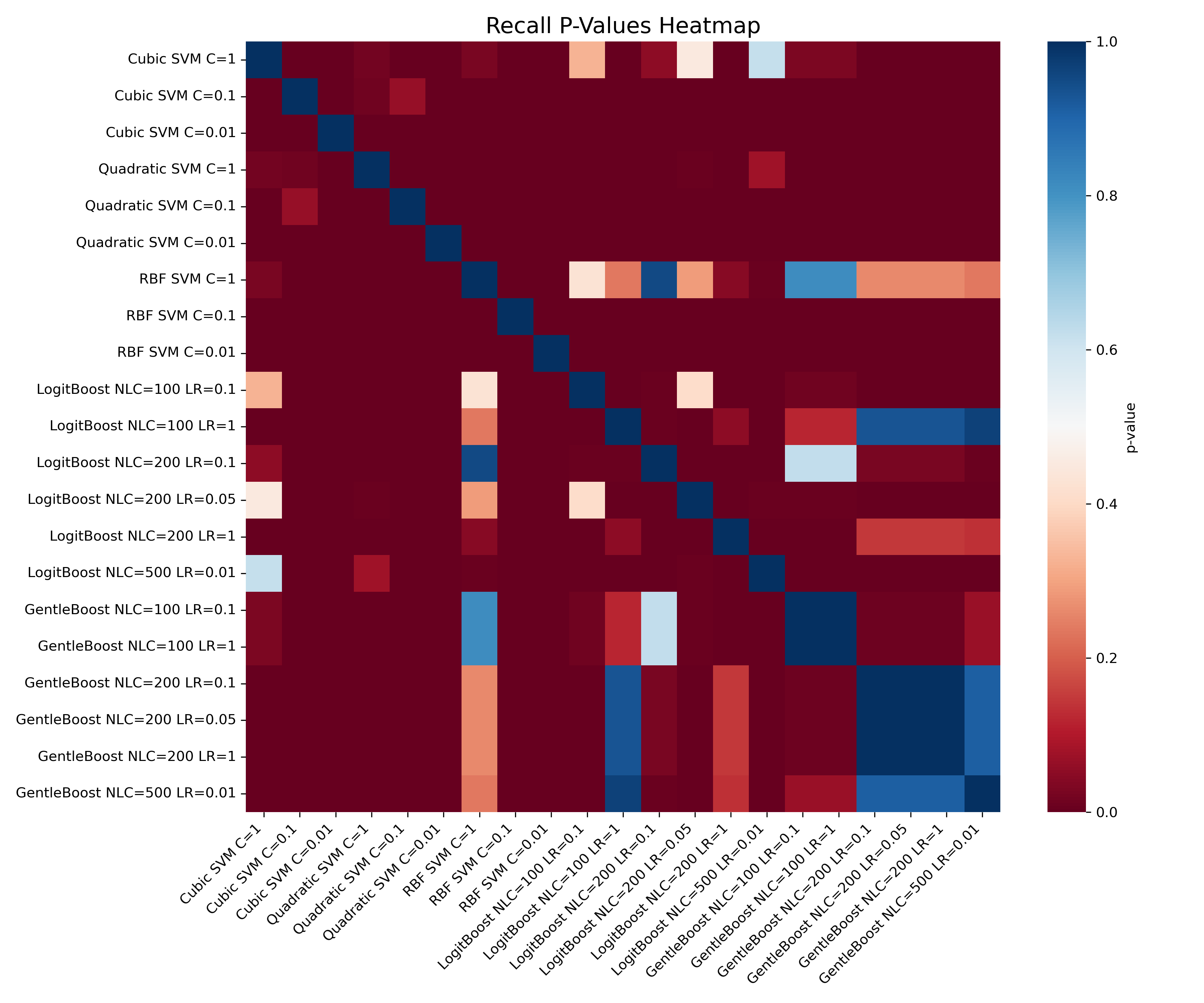

Supplement: Supplementary file 1 [file diagnostics-15-02065-s001.zip › Supplementary_File_24_Recall_P_Values_Heatmap.png]

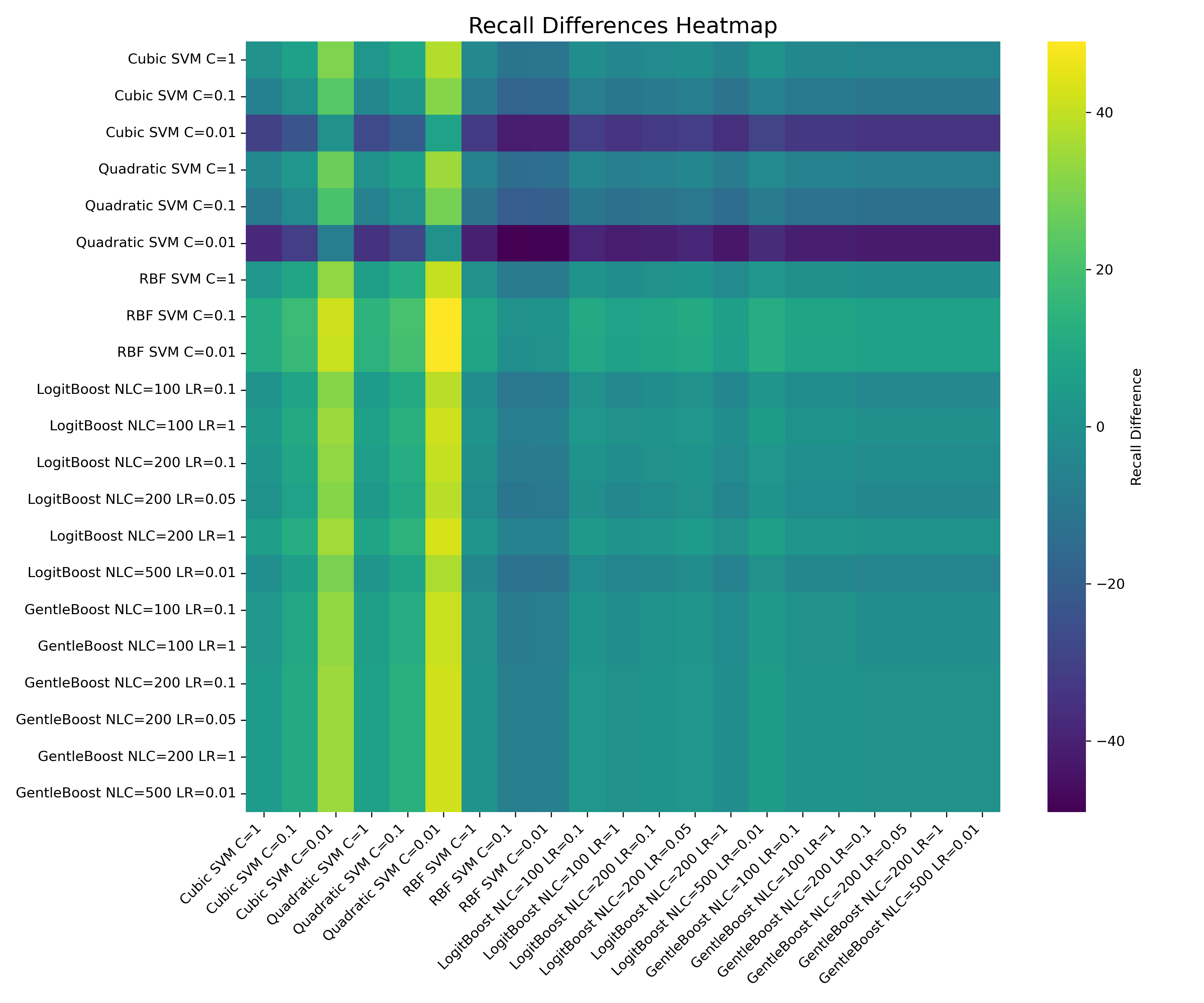

Supplement: Supplementary file 1 [file diagnostics-15-02065-s001.zip › Supplementary_File_25_Recall_Diff_Heatmap.png]

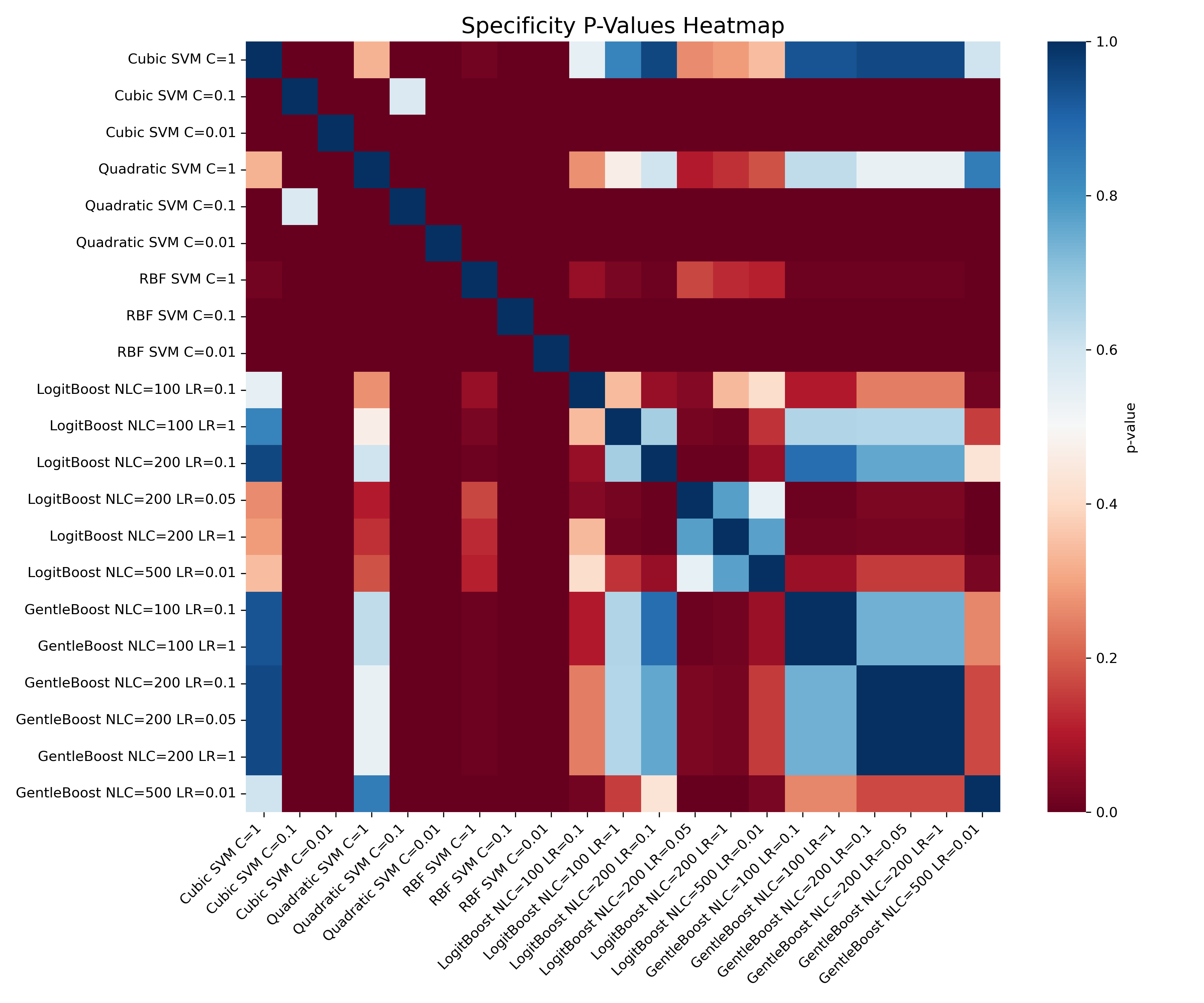

Supplement: Supplementary file 1 [file diagnostics-15-02065-s001.zip › Supplementary_File_27_Specificity_P_Values_Heatmap.png]

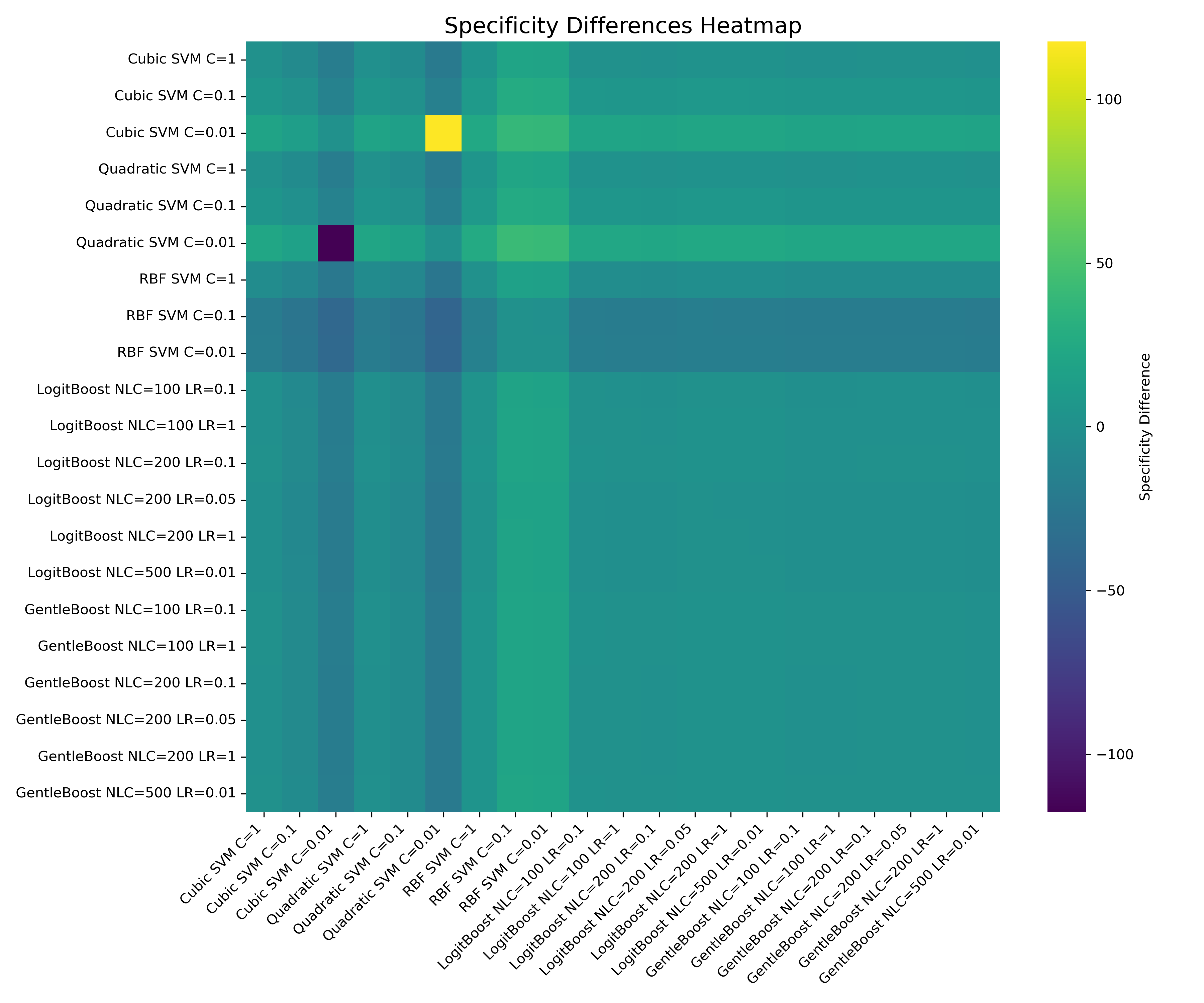

Supplement: Supplementary file 1 [file diagnostics-15-02065-s001.zip › Supplementary_File_28_Specificity_Diff_Heatmap.png]

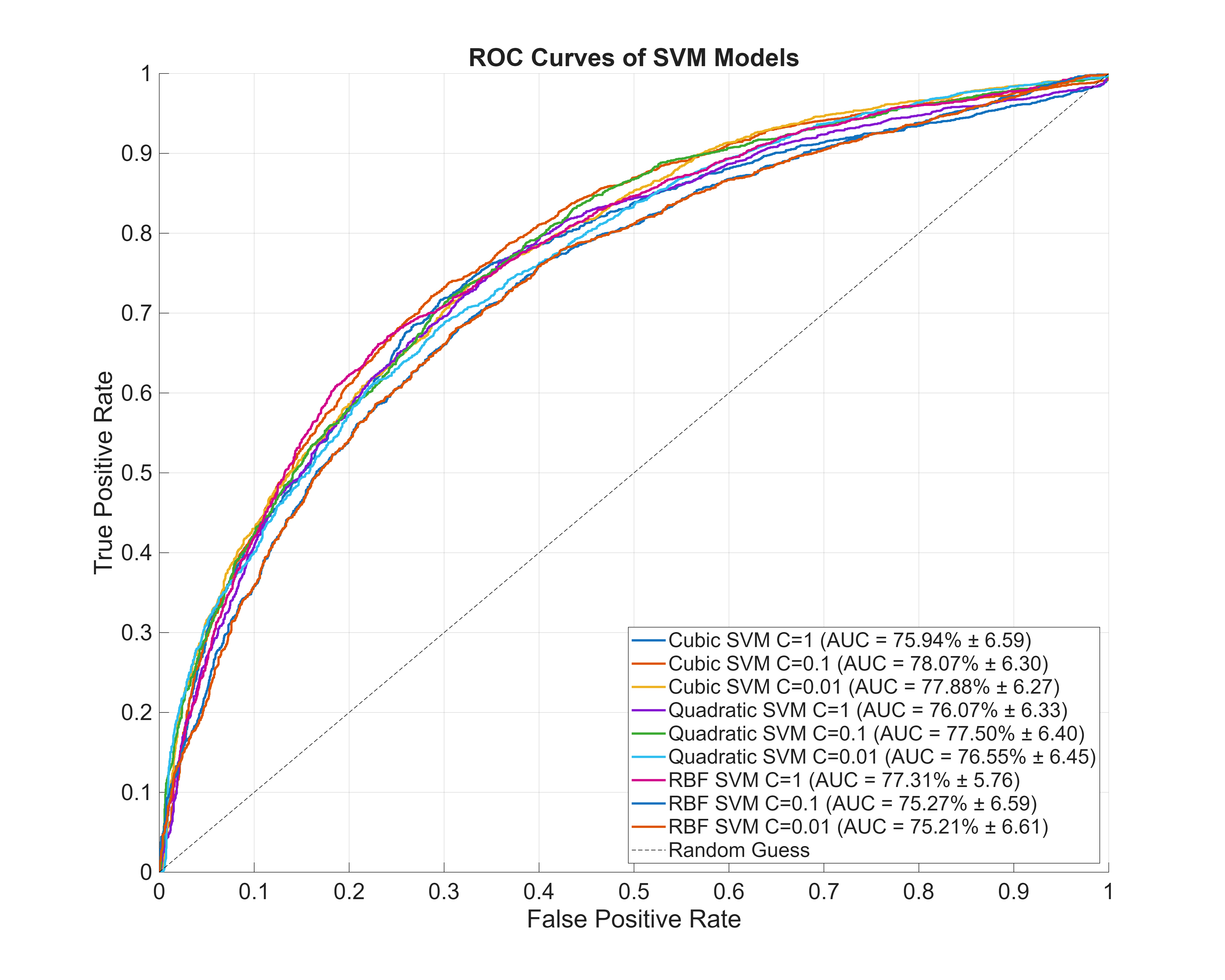

Supplement: Supplementary file 1 [file diagnostics-15-02065-s001.zip › Supplementary_File_5_ROC_SVM_Models.png]

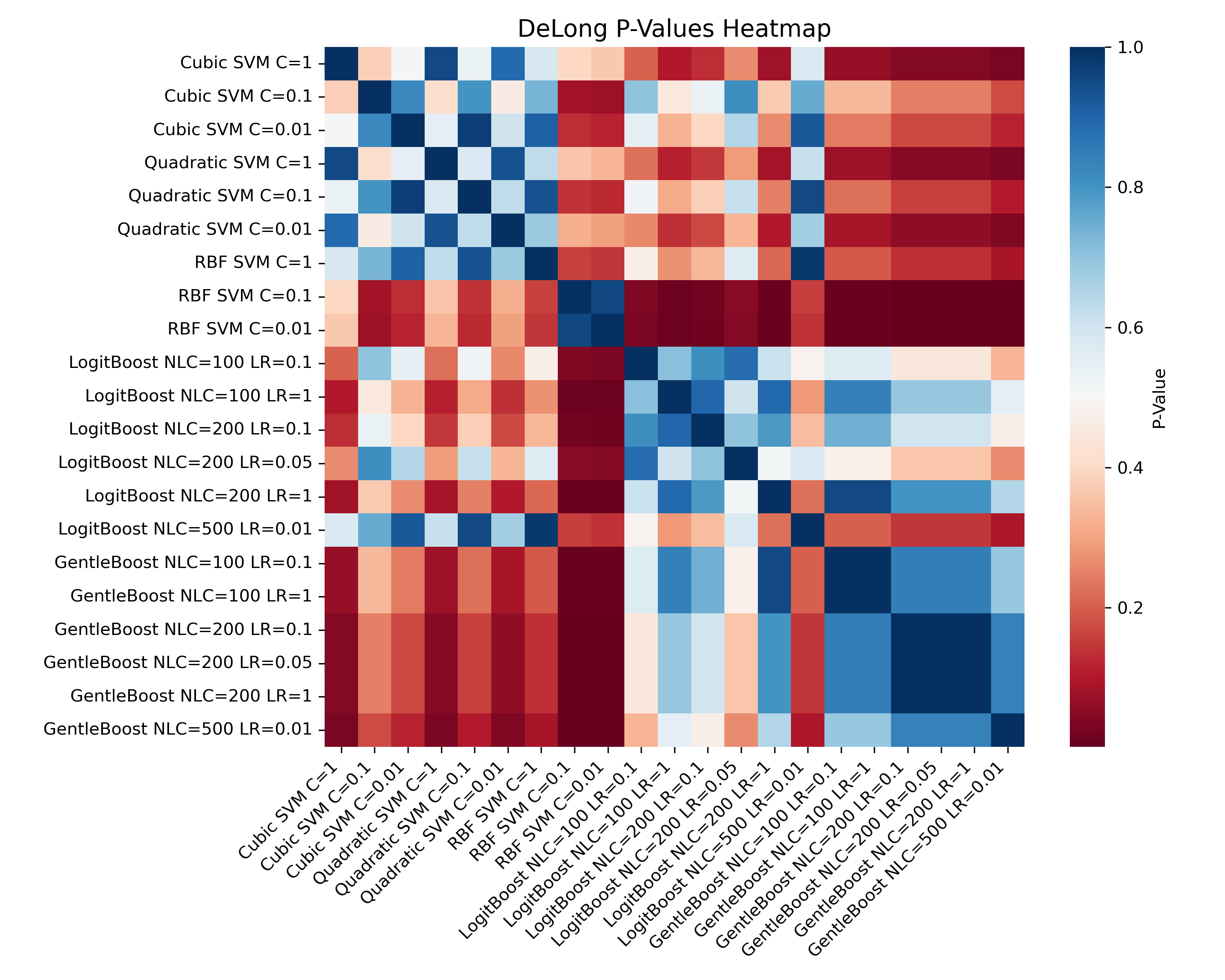

Supplement: Supplementary file 1 [file diagnostics-15-02065-s001.zip › Supplementary_File_7_DeLong_P_Values_Heatmap.png]

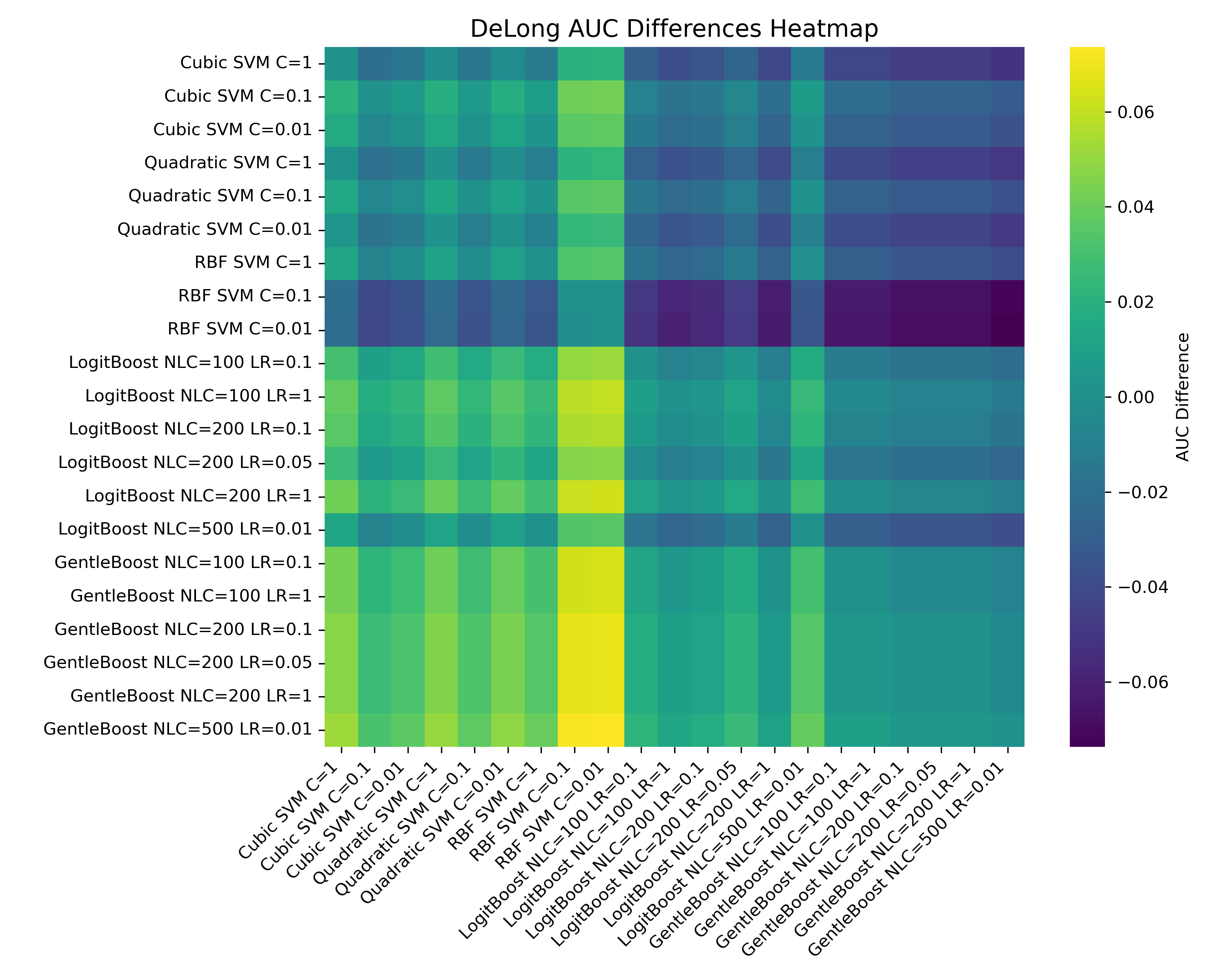

Supplement: Supplementary file 1 [file diagnostics-15-02065-s001.zip › Supplementary_File_8_DeLong_AUC_Differences_Heatmap.png]
